# Attenuating *Listeria monocytogenes* Virulence by Targeting the Regulatory Protein PrfA

**DOI:** 10.1016/j.chembiol.2016.02.013

**Published:** 2016-03-17

**Authors:** James A.D. Good, Christopher Andersson, Sabine Hansen, Jessica Wall, K. Syam Krishnan, Afshan Begum, Christin Grundström, Moritz S. Niemiec, Karolis Vaitkevicius, Erik Chorell, Pernilla Wittung-Stafshede, Uwe H. Sauer, A. Elisabeth Sauer-Eriksson, Fredrik Almqvist, Jörgen Johansson

**Affiliations:** 1 Department of Chemistry, Umeå University, 901 87 Umeå, Sweden; 2 Umeå Centre for Microbial Research (UCMR), Umeå University, 901 87 Umeå, Sweden; 3 Department of Molecular Biology, Umeå University, 901 87 Umeå, Sweden; 4 Molecular Infection Medicine, Sweden (MIMS), Umeå University, 901 87 Umeå, Sweden

## Abstract

The transcriptional activator PrfA, a member of the Crp/Fnr family, controls the expression of some key virulence factors necessary for infection by the human bacterial pathogen *Listeria monocytogenes*. Phenotypic screening identified ring-fused 2-pyridone molecules that at low micromolar concentrations attenuate *L. monocytogenes* cellular uptake by reducing the expression of virulence genes. These inhibitors bind the transcriptional regulator PrfA and decrease its affinity for the consensus DNA-binding site. Structural characterization of this interaction revealed that one of the ring-fused 2-pyridones, compound **1**, binds at two separate sites on the protein: one within a hydrophobic pocket or tunnel, located between the C- and N-terminal domains of PrfA, and the second in the vicinity of the DNA-binding helix-turn-helix motif. At both sites the compound interacts with residues important for PrfA activation and helix-turn-helix formation. Ring-fused 2-pyridones represent a new class of chemical probes for studying virulence in *L. monocytogenes*.

## Highlights

• Inhibitors of *L. monocytogenes* infectivity reduce virulence gene expression
• Binding of inhibitor to the PrfA regulator reduces affinity for its DNA motif
• First crystal structure of a Crp family regulator with an inhibitor
• Provides rationale for screening with Crp family transcriptional regulators

## Graphical Abstract

**Figure:**
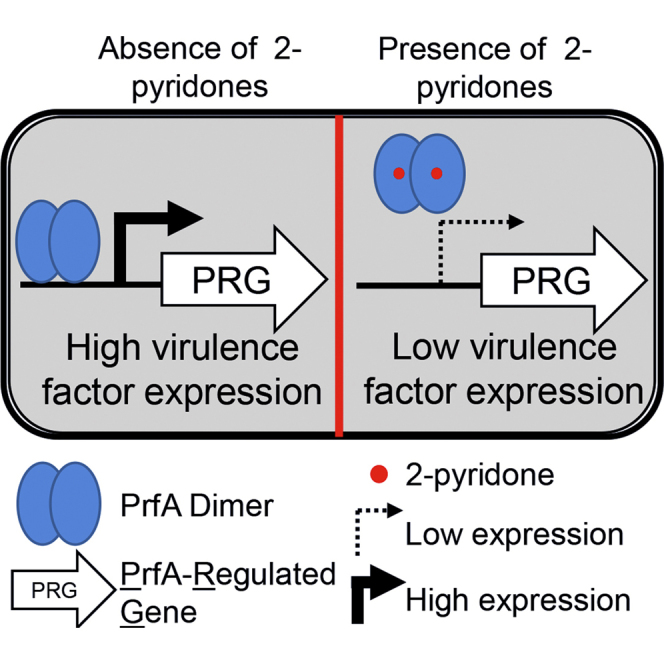

## Teaser

In this study, Good et al. identify ring-fused 2-pyridones which attenuate *Listeria monocytogenes* infectivity and reduce the expression of key virulence genes by binding to the central virulence regulator PrfA.

## Introduction

In light of increasing antibiotic resistance, novel therapies are required to potentiate or succeed our current selection of therapeutic options (Davies and Davies, 2010). An alternative to classical antibiotics are drugs inhibiting the virulence of pathogenic bacteria. The first step in establishing this as a viable and effective therapeutic strategy is to understand how the virulence of pathogenic bacteria can be controlled (Allen et al., 2014, Clatworthy et al., 2007, Rasko and Sperandio, 2010). The Gram-positive bacterium *Listeria monocytogenes* is a saprophyte responsible for the severe disease listeriosis in humans upon ingestion (Freitag et al., 2009, Vázquez-Boland et al., 2001). Its ability to grow at low temperatures, in high-salt and low-oxygen conditions, makes *L. monocytogenes* one of the most problematic foodborne pathogens. Although the incidence rate is low, *L. monocytogenes* is capable of crossing key protective barriers within the body (e.g., intestinal, placental, and blood-brain) and causing severe diseases (e.g., bacteremia, meningitis, and meningoencephalitis) (Drevets and Bronze, 2008). Pregnant, immunocompromised, and other at-risk patients are vulnerable to invasive listeriosis, and the high mortality rates within these subpopulations (∼20%–40%) are a stark demonstration of the clinical difficulty in dealing with these infections (Drevets and Bronze, 2008, Hamon et al., 2006, Jackson et al., 2010, Vázquez-Boland et al., 2001).

The virulence factors governing host invasion and infection by *L. monocytogenes* have been well elucidated (Vázquez-Boland et al., 2001). The bacterium adheres to and enters both phagocytic and non-phagocytic cells, by using specific adhesins (e.g., InlA and InlB), depending on the cell type. With the aid of listeriolysin O (LLO) and a phospholipase (PlcA), the bacterium lyses the phagosome and proceeds to replicate intracellularly (Schnupf and Portnoy, 2007). Once in the cytoplasm, the bacterium uses the ActA protein to recruit the Arp2/3 complex facilitating the formation of an actin comet-tail, by which *L. monocytogenes* is able to move to an adjacent cell without exposing itself to the extracellular environment. ActA has also been shown to play a role during bacterial attachment and uptake into epithelial cells (Garcia-Del Portillo and Pucciarelli, 2012, Suarez et al., 2001). Within the next cell, the bacterium degrades the double-membrane vacuole and perpetuates the infection cycle (Hamon et al., 2006, Portnoy et al., 2002). The expression of the majority of virulence genes required for these processes is regulated by PrfA, a transcriptional activator from the Crp/Fnr family of regulators (Scortti et al., 2007). Members of this family bind as homodimers to consensus DNA sequences found in the promoter region of regulated genes (Won et al., 2009). PrfA positively regulates the expression of the above and other Listerial virulence factors (Freitag et al., 2009, Scortti et al., 2007), and a Δ*prfA* strain is avirulent (Andersson et al., 2015, Chakraborty et al., 1992, Freitag et al., 1993, Gripenland et al., 2014). Structurally, each monomer of PrfA comprises an N-terminal eight-stranded β-barrel domain connected by an α helix linker to a C-terminal α/β domain (Eiting et al., 2005, Vega et al., 2004). The C-terminal region contains the winged helix-turn-helix (HTH) motif responsible for binding to consensus promoter sequences (Eiting et al., 2005, Vega et al., 2004). While most Crp family members require a small molecule cofactor for DNA binding (e.g., cAMP for Crp in *Escherichia coli*) (Won et al., 2009), PrfA is capable of binding to its DNA consensus sequences with low affinity even in the absence of a cofactor (Eiting et al., 2005). Nonetheless, the activity of PrfA is known to increase under inducible conditions, and this has led to the hypothesis that the intercellular mechanism of activation may be regulated by a host-derived or host-regulated cofactor (Freitag et al., 2009, Scortti et al., 2007). Recently, it was suggested that PrfA activation follows a two-step process, where PrfA needs to be in a reduced form for DNA binding, followed by interaction of PrfA with reduced glutathione for transcriptional activity at targets genes (Reniere et al., 2015). Structural evidence toward an allosteric mode of activation for PrfA in vivo has been provided by the crystal structure of the constitutively active PrfA mutant PrfA_G145S_ (Eiting et al., 2005, Ripio et al., 1997, Vega et al., 2004). This amino acid substitution repositions the HTH motif in an ordered and exposed “active” conformation with increased DNA-binding affinity, in contrast to PrfA_WT_ where the HTH motif remains flexible and partially disordered (Eiting et al., 2005).

We previously developed ring-fused 2-pyridone scaffolds which contain a peptidomimetic backbone (Svensson et al., 2001). Originally designed to mimic interactions between subunits in the chaperone-usher pathway responsible for pilus assembly in uropathogenic bacteria, we subsequently developed inhibitors with discrete substitution patterns from this scaffold, which both interact with structures important for *E. coli* adhesion to eukaryotic cells (Cegelski et al., 2009, Emtenäs et al., 2002, Pinkner et al., 2006) and had broader impact on virulence regulation (Greene et al., 2014). We were therefore interested to investigate whether 2-pyridones could affect *L. monocytogenes* virulence-associated phenotypes. In this study, we have conducted a phenotypic screen and identified several ring-fused 2-pyridones that attenuate *L. monocytogenes* uptake into epithelial cells and decrease virulence gene expression. We describe how these inhibitors interact directly with the transcriptional regulator PrfA and weaken its DNA-binding capacity. Furthermore, we provide the first structural detail of a Crp family protein with a bound inhibitor by presenting the crystal structure of PrfA in complex with one 2-pyridone, and propose possible modes of action.

## Results

### Ring-Fused 2-Pyridones Attenuate *L. monocytogenes* Uptake

**Figure 1:**
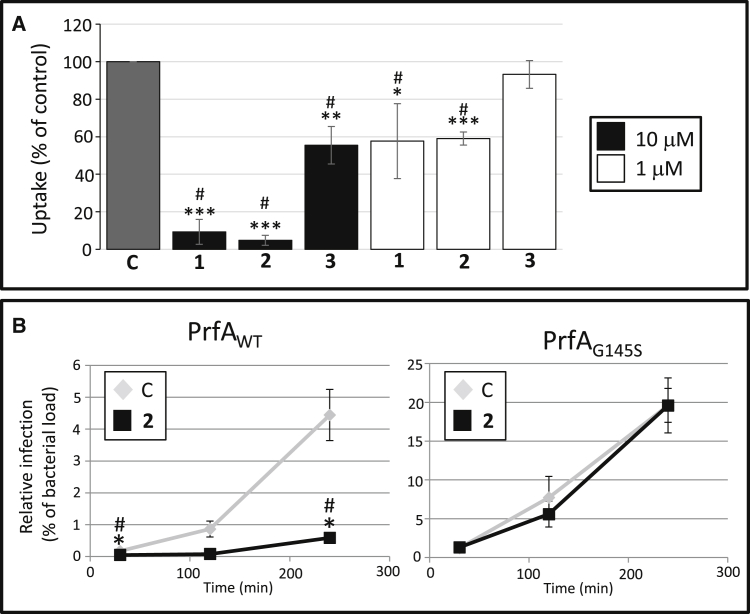
Ring-Fused 2-Pyridones Inhibit *L. monocytogenes* Uptake by Cultured Cells (A) Relative uptake (%) compared with control as determined by viable count measurements. *L. monocytogenes* strain EGDe (WT) treated with compounds **1–3** at the indicated concentrations (10 μM, black bars; 1 μM, white bars) was allowed to infect HeLa cells for 1.5 hr. All samples were correlated to the DMSO control (C, gray bar), which was arbitrarily set at 100%. Error bars show SDs. Significance was tested using Student's t test (two-tailed, significant differences are shown by asterisks; *p < 0.05; **p < 0.01, and ***p < 0.001) and Dunnett's test (significant differences are shown by #). See also Figure S1. (B) Time-course infection dynamics of *L. monocytogenes* harboring PrfA_WT_ or PrfA_G145S_ after treatment with compound **2**. Caco-2 cells were infected with *L. monocytogenes* strains carrying PrfA_WT_ (left panels) or PrfA_G145S_ (right panels) on the chromosome in the presence of DMSO (C) or compound **2** (50 μM). The amount of intracellular bacteria was measured using viable counts at indicated time points post infection and divided by the bacterial load used in the infection (prior to antibiotic treatment) arbitrarily set as % (n = 3). It should be noted that infection of Caco-2 cells with a strain harboring PrfA_G145S_ is more effective than with a strain carrying PrfA_WT_. Error bars show SDs. Significance was tested using Student's t test (two-tailed, significant differences are shown by asterisks; *p < 0.05) and Dunnett's test (significant differences to the control are shown by #).

Using flow cytometry, we performed an infection screen based on HeLa cells infected with GFP-carrying *L. monocytogenes*. The putative virulence-inhibiting ability of ring-fused 2-pyridones from our in-house collection was assessed, and several close analogs that significantly reduced the relative infection at 100 μM were identified (Figures S1A and S1B). At a concentration of 10 μM, two 1-naphthyl derivatives effectively reduced *L. monocytogenes* uptake by HeLa cells by 80%–90% (C10 [**1**] and KSK 67 [**2**]), whereas a related 3-quinoline analog, KSK 29 (**3**), was less effective (Figure S1B). Viable count experiments verified these flow cytometry results, with compounds **1** or **2** reducing the level of *L. monocytogenes* uptake relative to untreated controls at 10 and 1 μM (Figure 1A). Furthermore, when we performed a time-course experiment monitoring the infection dynamics more closely, we observed an inability of *L. monocytogenes* to replicate within Caco-2 cells after treatment with compound **2**, which displayed the largest efficiency in the uptake experiment (Figure 1B, left panel). Although the generation time of *L. monocytogenes* was slightly longer in presence of compounds **1** or **2** at 100 μM compared with the DMSO control (45, 50, and 43 min, respectively), it is unlikely that this would be sufficient to explain the dramatic decrease in infectivity observed with these compounds at 100 μM and at lower concentrations (Figures S1C and 1). The generation time in the presence of compound **3** was unaffected compared with the DMSO control (43 min, Figure S1C).

### Ring-Fused 2-Pyridones Reduce Virulence Factor Expression in *L. monocytogenes*

**Figure 2:**
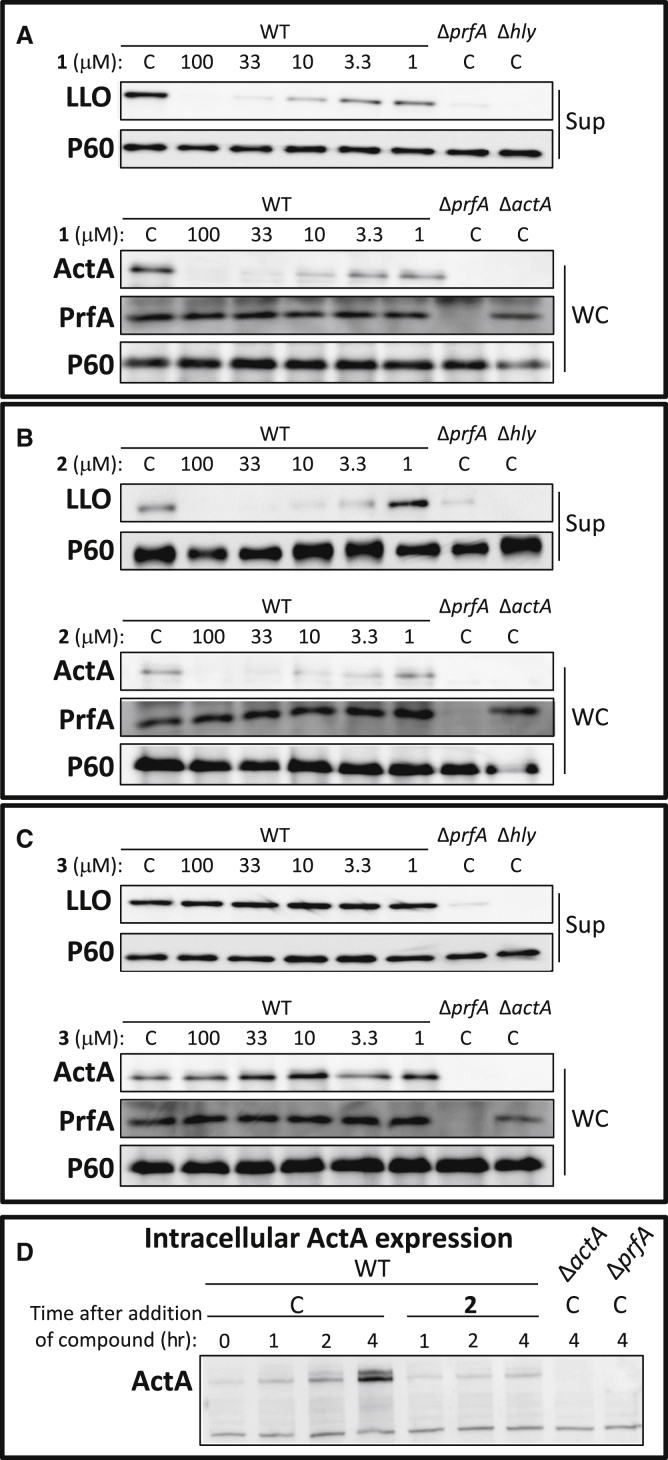
Compounds **1** and **2** Reduce Virulence Factor Expression in *L. monocytogenes* (A–C) Protein extracts were isolated from indicated *L. monocytogenes* strain in the absence (C, equivalent volume of DMSO) or presence of **1** (A), **2** (B), or **3** (C) at the indicated concentration, and the specified proteins (ActA, LLO, PrfA, or P60 (control)) were detected by western blot using specific antibodies. Upper panels show secreted fractions (Sup) of indicated samples; lower panels show whole cells fractions (WC) of indicated samples. The images are a representative of three individual experiments. (D) Compound **2** abrogates intracellular induction of ActA expression. Caco-2 cells were infected with the indicated bacteria and treated 30 min post infection with 100 μM of **2** or the equivalent volume of DMSO (C). Total cellular protein was extracted at indicated time points after addition of compound and subjected to SDS-PAGE. The levels of ActA protein were visualized by western blot using a specific antibody. The images are a representative of three individual experiments.

**Figure 3:**
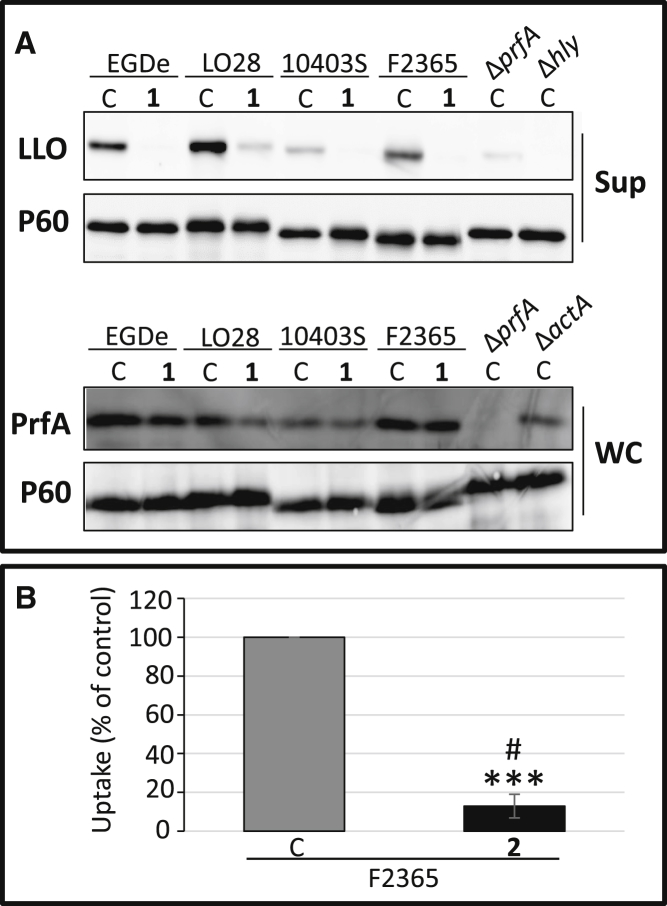
Compounds **1** and **2** Reduce Virulence Factor Expression and Uptake of Multiple Serotypes of *L. monocytogenes* (A) Compound **1** reduces LLO expression in different *L. monocytogenes* strain backgrounds. Protein extracts were isolated from indicated *L. monocytogenes* strain in the absence (C, equivalent volume DMSO) or presence of 100 μM of **1** and the specified proteins (LLO PrfA, or P60 (control)) were detected by western blot using specific antibodies. Upper panels show secreted fractions (Sup) of indicated samples; lower panels show whole-cell fractions (WC) of indicated samples. (B) Compound **2** inhibits the uptake of an *L. monocytogenes* strain of serovar 4b. *L. monocytogenes* strain F2365 was allowed to infect Caco-2 cells for 2 hr with **2** (50 μM). All samples were correlated to the DMSO-treated control (C, gray bar) which was arbitrarily set at 100%. Error bars show SDs. Significance was tested using Student's t test (two-tailed, significant differences are shown by asterisks; ***p < 0.001) and Dunnett's test (significant differences to the control were shown by #).

To understand how the compounds attenuated the infectivity of *L. monocytogenes*, the expression of virulence genes was analyzed in bacteria grown with or without 100 μM of **1**, **2**, or **3**. We initially investigated the expression of the *hly* gene which encodes the hemolysin LLO, and found it was downregulated by **1** or **2** compared with the untreated control, but not affected by compound **3** (Figure S2). Together with most virulence genes in *L. monocytogenes, hly* is positively regulated by the transcriptional activator PrfA (Freitag et al., 2009, Scortti et al., 2007), therefore we examined whether the expression of other PrfA-regulated genes was affected by treatment with compounds **1** and **2**. A reduction in the levels of the virulence genes *hly, actA*, and *plcA* transcripts could be observed after treatment with compounds **1** and **2** (but not **3**), whereas the expression of *inlA* and *inlB* was unaffected (Figure S2), possibly because basal expression of the latter are also controlled by other regulatory factors, such as the stress sigma factor σ^B^ (Stritzker et al., 2005). We next investigated whether the virulence protein levels of the two major virulence factors, LLO and ActA, were affected by compound treatment. Of the 2-pyridones tested, compounds **1** and **2** displayed similar properties. At 100 μM of **1** or **2**, expression of LLO and ActA was abolished, without a concomitant effect on PrfA protein levels or on the expression of the non-PrfA-regulated virulence factor P60 (Figures 2A and 2B). The reduction in LLO and ActA expression following treatment with **1** and **2** was dose dependent, with decreased levels down to ≤3.3 μM (Figures 2A and 2B). In contrast, compound **3** did not reduce LLO or ActA expression even at the highest concentration tested of 100 μM, in agreement with the lack of efficacy of **3** in abrogating *hly* or *actA* gene expression (Figures 2C and S2). Across all concentrations of **1**, **2**, or **3**, PrfA expression remained unchanged, indicating that the virulence attenuation of **1** and **2** was not mediated through reduced PrfA protein levels (Figures 2A–2C). Once a host cell is invaded by *L. monocytogenes*, ActA expression is massively induced in a PrfA-dependent manner (Freitag et al., 2009, Scortti et al., 2007). We therefore analyzed whether ActA expression was affected at different time points when 100 μM of compound **2** was added 30 min post infection. The induction of intracellular ActA levels observed in the DMSO control 4 hr post infection, was weakened in compound-treated cells, although not completely abolished (Figure 2D). This indicates that the effectiveness of the compounds decreased post infection. We next examined whether ring-fused 2-pyridones could also reduce virulence factor expression in other *L. monocytogenes* strain backgrounds or serotypes. Addition of compound **1** effectively reduced LLO expression in *L. monocytogenes* serotypes associated with sporadic cases (10403S [serotype 1/2a] and LO28 [serotype 1/2c]) as well as a serotype associated with epidemic outbreaks (F2365 [serotype 4b]), whereas the levels of PrfA were unaffected in these strains (Figure 3A). In addition, compound **2** effectively inhibited uptake of the F2365 strain into Caco-2 cells (Figure 3B).

### Virulence Regulation by the Mutant PrfA_G145S_ Is Not Affected by Ring-Fused 2-Pyridones

**Figure 4:**
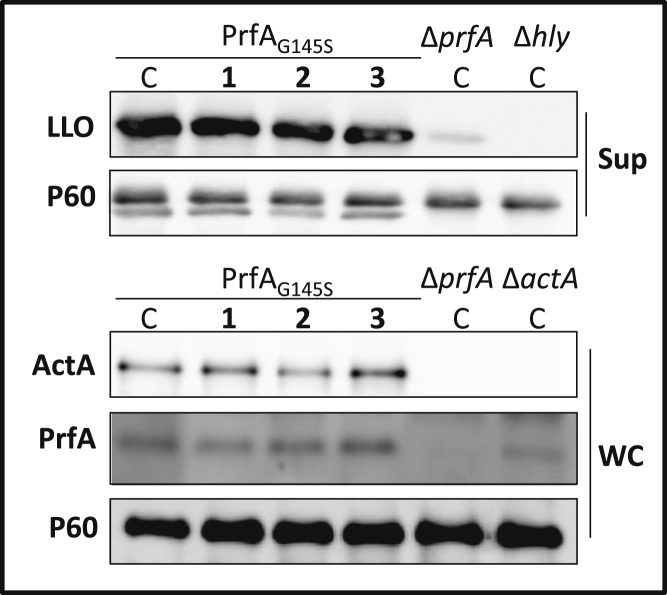
Compounds **1**, **2**, and **3** Cannot Inhibit LLO and ActA Expression in a Strain Expressing a Constitutively active PrfA Protein PrfA_G145S_ The indicated strains were grown with **1–3** at 100 μM or DMSO (C). Upper panels show secreted fractions (Sup) of indicated samples; lower panels show whole-cell fractions (WC) of indicated samples. The levels of LLO, ActA, PrfA, and P60 (control) proteins are visualized by western blot using specific antibodies. The images are a representative of three individual experiments. See also Figure S2.

The reduced levels of the virulence factors LLO and ActA, but not of PrfA (Figures 2A and 2B), suggested that **1** and **2** directly affected the activity of PrfA. Introducing a G145S amino acid substitution in PrfA generates a constitutively active mutant that contains a stabilized HTH DNA-binding motif, as opposed to the structurally undefined HTH present in PrfA_WT_ (Eiting et al., 2005, Ripio et al., 1997). Examining the effect of the compounds on PrfA_G145S_ in comparison with PrfA_WT_ would indicate whether **1** and **2** directly inhibited the PrfA activation process, or if their effects were mediated after formation of the HTH DNA-binding motif. To test this, a *prfA*_G145S_ allele was introduced at its native site on the chromosome, such that PrfA_G145S_ would be produced, and this strain was treated with **1**, **2**, or **3** at 100 μM. In the PrfA_G145S_-expressing strain, virulence gene expression as well as virulence protein levels remained essentially unaltered in the presence of the compounds (Figures 4 and S2). Once again, the levels of PrfA and P60 proteins did not vary considerably after treatment with the different compounds. We next challenged whether this mutation could also overcome the inhibitory effects of these compounds in a time-course cellular infection assay, with the most effective compound from the cellular uptake experiments, compound **2**. While *L. monocytogenes* containing PrfA_WT_ was unable to replicate within Caco-2 cells in presence of **2** at 100 μM (Figure 1B, left panel), a strain expressing PrfA_G145S_ overcame the inhibitory effects and established an infection comparable with the untreated control (Figure 1B, right panel). Collectively, these data strongly indicated that the 2-pyridones **1** and **2** reduced *L. monocytogenes* virulence by attenuating PrfA activity, and furthermore suggested that this process occurred prior to formation of the DNA-binding HTH motif, since the PrfA_G145S_ protein carrying the stabilized HTH motif was not affected by the compounds.

### Ring-Fused 2-Pyridones Directly Bind to PrfA In Vitro

**Figure 5:**
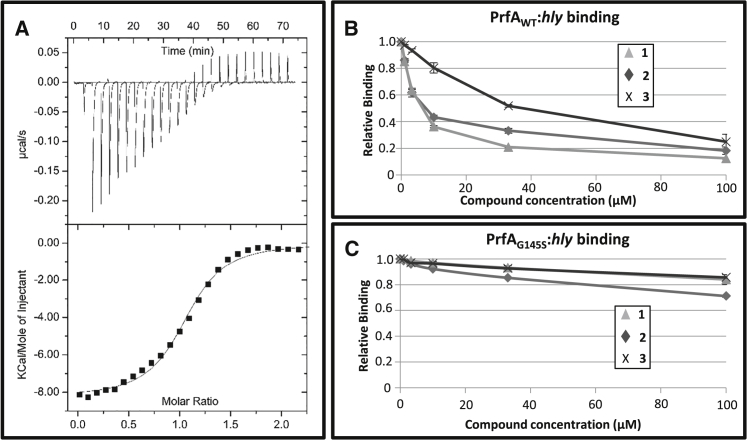
Ring-Fused 2-Pyridones Bind to PrfA and Decrease the Affinity of PrfA_WT_, but Not PrfA_G145S_, for *hly*-DNA (A) ITC titration of **1** to PrfA_WT_. Upper panel, heat pulses versus injections; lower panel, integrated heat signals versus molar ratio of **1** to PrfA_WT_. (B) and (C) SPR plots of relative binding affinity of PrfA_WT_ and PrfA_G145S_ with **1–3** to *hly*-DNA. PrfA_WT_ (B) or PrfA_G145S_ (C) was incubated with the indicated amounts of **1**, **2**, or **3**. The samples were analyzed by SPR and the relative binding capacity plotted. All compounds were compared with DMSO, which was set to 100% (n = 3). Data represents the mean values with SDs.

We next determined whether **1** and **2** directly bound to PrfA via isothermal titration calorimetry (ITC). This in vitro method, with purified PrfA protein, allows measurement of the thermodynamic parameters of binding from the heat development that occurs upon ligand-protein interactions (Ladbury et al., 2010). The data presented in Figure 5A show that binding of **1** to PrfA_WT_ occurs with negative enthalpy in a 1 to 1 stoichiometry per monomer of homodimeric PrfA. From analysis of the integrated heat peaks as a function of ligand-to-protein ratio using a 1 to 1 binding model, a dissociation constant (*K*_D_) value for **1** binding to PrfA of ≈1 μM was determined (Table 1). Compound **2**, and unexpectedly **3**, also exhibited micromolar affinities for PrfA_WT_ and interacted in 1 to 1 stoichiometry (Table 1 and Figure S3). We examined the binding of compounds **1–3** to the constitutively active PrfA mutant PrfA_G145S_ and found that all three ligands bound to PrfA_G145S_ with comparable affinity for **2** and reduced affinity for **1** and **3** (Table 1). These data suggested that the compounds do not bind at the DNA-binding HTH motif, since this region differs in conformation between the mutant (folded) versus the wild-type (WT) (partially disordered) (Eiting et al., 2005).

### Compounds **1** and **2** Inhibit the DNA Binding of PrfA_WT_, but Not PrfA_G145S_

To further characterize the binding of **1–3** with PrfA in vitro, and analyze if such binding affects the DNA-binding properties of PrfA, we performed surface plasmon resonance (SPR) experiments. In these in vitro experiments, the ability of PrfA (WT or G145S), pre-incubated with **1**–**3**, to bind the *hly*-promoter DNA was analyzed. Compounds **1** and **2**, which reduced infectivity and the expression of key virulence factors in the cellular assays, both effectively reduced the binding of PrfA_WT_ to *hly*-DNA at low micromolar levels via SPR (half maximal inhibitory concentration [IC_50_] ≈ 6–7 μM; Figure 5B). The quinolone-containing compound **3**, which had only a slight effect in the cellular assays and did not attenuate virulence factor expression, could only inhibit the binding of PrfA_WT_ to *hly*-DNA at higher concentrations (IC_50_ ≈ 30 μM), indicating it was less effective at preventing the PrfA-*hly* interaction (Figure 5B). Furthermore, all the compounds had minimal impact upon the binding of PrfA_G145S_ to *hly*-DNA at concentrations up to 100 μM (Figure 5C). Taken together, we conclude that while **1–3** bind to the constitutively active mutant PrfA_G145S_, this has no significant impact on its ability to bind DNA.

### Crystal Structure of PrfA_WT_-**1** Complex

**Figure 6:**
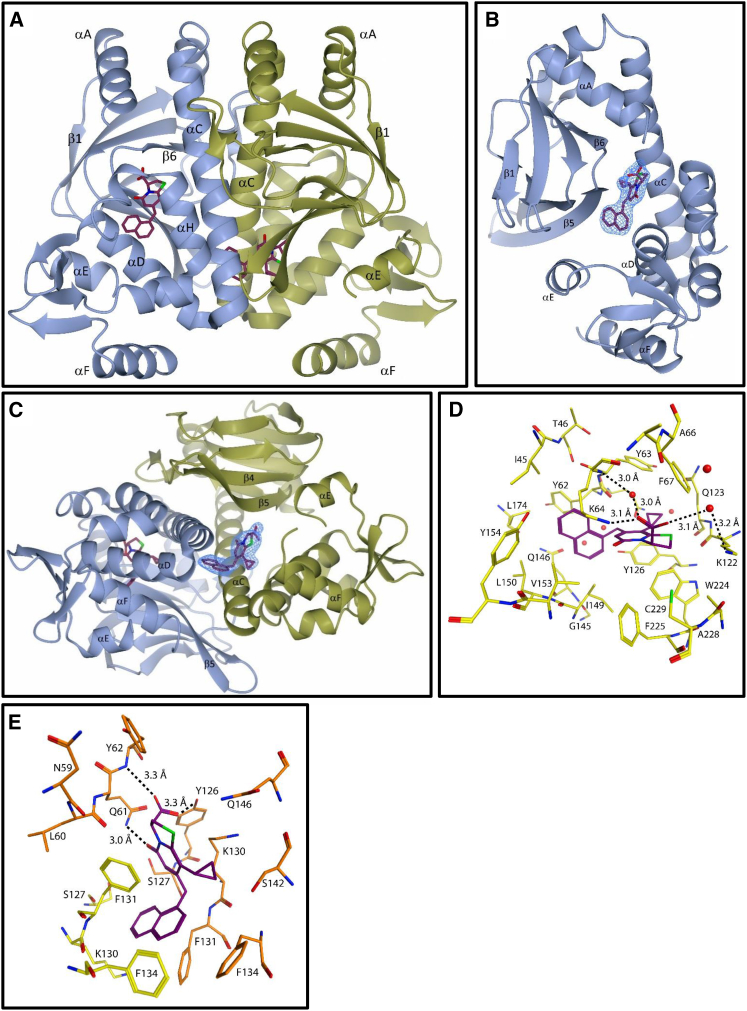
Structure of the PrfA_WT_-**1** Complex (A) The PrfA_WT_ homodimer showing the binding site of **1**. The monomeric units A and B of PrfA_WT_ are colored in blue and gold, respectively. Two ligands bound to each monomer at two different sites, are shown as sticks. (B and C) Quality of the electron density for compound **1** bound (A) to monomer A (site AI) and (C) to monomer B (site BII). The σ^A^-weighted (2m|F_o_| − D|F_c_|) electron density calculated from the refined PrfA-**1** complex is contoured at the root-mean-square deviation value of the map and shown in blue over compound **1** only. (D) and (E) Key local structural features and amino acids in proximity to **1** at site AI (D) and BII (E). The ligand is colored by atom type: purple (C), green (S), blue (N), and red (O) and protein residues are colored by atom type: monomer A (C) yellow and monomer B (C) orange, green (S), blue (N), and red (O). Hydrogen bonds are indicated with dashed lines.

To understand the structural basis for inhibition, we determined the crystal structure of the PrfA_WT_-**1** complex at a resolution of 2.25 Å (Table 2). The PrfA_WT_-**1** complex was co-crystallized in the space group P2_1_ where the asymmetric unit contains one biological dimer: monomers A and B (Figure 6A). Binding of **1** to PrfA_WT_ was confirmed at two sites, AI in monomer A and BII in monomer B, from difference Fourier electron density maps (Figures 6B, 6C, and S4).

The PrfA_WT_-**1** dimer structure is similar to the previously determined structures of PrfA_WT_ (Eiting et al., 2005) (Figures 6A and S5). Each monomer consists of an N-terminal domain (residues 1–108) and a C-terminal DNA-binding domain (residues 138–237) linked by a long α helix (αC, residues 109–137). Both the N- and C-terminal domains constitute an α/β fold. Hydrophobic interactions between symmetry-related αC helices and loops β6-β7 stabilize the dimer interface (Figure 6A). Two α helices in the C-terminal domain, αE (residues 170–178) and αF (residues 183–195) constitute the helices of the typical HTH motif present in many prokaryotic transcription factors. In PrfA_WT_, parts of the first helix and the connecting turn of the PrfA HTH motif were not defined by electron density and have been assumed to be flexible (Eiting et al., 2005). This was also the case for the PrfA_WT_-**1** complex structure determined here.

Compound **1** binds PrfA at two sites referred to as AI and BII. Site AI is found at the so-called interdomain tunnel in monomer A, situated between the N- and C-terminal domains (Figure 6B). The interdomain tunnel is positioned between the C-terminal helices αH and αI and the secondary structural motifs αC, αD, and β5 (Eiting et al., 2005). Compound **1** binds with high occupancy at site AI, but no ligand bound to the tunnel in monomer B (i.e., to the BI site). At binding site AI the carboxylate is in close proximity with the charged ɛ-amino groups of Lys64 and Lys122, indicating the potential for both electrostatic interactions and a network of hydrogen bonding, which in the case of Lys122 are mediated through the bulk solvent (Figure 6D). The naphthyl ring occupies a hydrophobic part of the tunnel bounded by the peptide backbones of Tyr62, Gln146, Ile149, and Leu150, and the cyclopropyl motif is surrounded between the peptide backbone of Gln123 and the phenyl rings of Tyr63, Phe67, and Tyr126, respectively. We reason that the peptidomimetic bicyclic 2-pyridone backbone provides the key recognition motif for binding to PrfA at the AI site, with the carboxylate forming electrostatic interactions with residues known to affect the activation of PrfA (Lys64 and Lys122) (Xayarath et al., 2011), and the naphthyl motif providing a lipophilic anchor into the interdomain tunnel. The second high-occupancy binding site for compound **1**, i.e., the BII site, is found at a hydrophobic pocket in the vicinity of the HTH motif in monomer B (Figure 6C). No ligand bound to the corresponding site in monomer A (i.e., to the AII site). The BII site is positioned in a pocket formed by the αC and αD helices and β5 strand from monomer B and the C-terminal end of the αC helix from monomer A (Figure 6C). At site BII, the carboxylate group of compound **1** forms hydrogen bonds to the main chain nitrogen atom of B-Tyr62, and to the hydroxyl group of B-Tyr126 and the 2-pyridone carbonyl hydrogen bonds with the side chain amino group B-Gln61. The naphthyl group of compound **1** at site BII is sandwiched between the side chains of four phenylalanines: Phe131 and Phe134 from both monomers in the dimer (Figure 6E). The charged lysine of B-130 is also sufficiently close (∼4.8 Å) for electrostatic interactions with the carboxylate group to affect the free energy of binding.

## Discussion

In this work, we identified ring-fused 2-pyridone inhibitors by phenotypic screening which effectively attenuated *L. monocytogenes* uptake into cultured cells at low micromolar concentrations. Compounds **1** and **2**, which were investigated in most detail, exhibited similar reductions in virulence and infectivity across all assays. A structurally discrete third analog, **3**, consistently failed to attenuate uptake although still binding PrfA in vitro, which may be attributable to lower intrinsic efficacy (Figure 5B) and less optimal physicochemical properties than **1** and **2**. Collectively, the results indicate that **1** and **2** act by binding to and preventing the activation of PrfA, the central transcriptional regulator of virulence in *L. monocytogenes*. The ring-fused 2-pyridone molecules bind to purified PrfA with *K*_D_ values in the low micromolar range (Table 1 and Figure 5). Consequently, this lowers the affinity of the protein for its DNA consensus sequences within the promoter regions of virulence genes (Figure 5B). This reduced DNA-binding affinity in turn abolishes the expression of key virulence factors, which reduces uptake in epithelial cells (Figures 1, 2, and 3; Figures S1 and S2).

Our functional and structural studies support a model where **1** and related inhibitors act either by preventing the structural rearrangements needed for activation of PrfA through stabilization of the HTH motif, or alternatively by preventing the concerted reorganization of the monomers that move the DNA-binding domains into positions compatible with DNA binding (Levy et al., 2008, Schultz et al., 1991). Previous structural studies on PrfA and the constitutively active PrfA_G145S_ mutant revealed that rearrangements at the dimer interface enable the stabilization of the HTH motif in PrfA_G145S_ into a structurally defined conformation with increased affinity to DNA (Eiting et al., 2005). Comparison between the two structures suggested that for DNA binding to occur, helix αC needs to straighten leading to rearrangements of β4-β5. Phe134, positioned on αC, flips over to fill the void created between helices αC and αD. Furthermore, Tyr62 positioned at β5 moves to fill the same void and to bring the side chains of Ile57 and Asn59 into direct contact with the now structurally defined first helix in the HTH motif (Eiting et al., 2005). Compound **1** binds to site AI, a hydrophobic interdomain tunnel previously suggested to constitute a plausible cofactor binding site (Eiting et al., 2005) and to site BII, a hydrophobic pocket in the vicinity of the HTH motif (Figures 6, S6A, and S6B). We reason that inhibitor binding at AI could prevent conformational changes of the PrfA protein, and thus block the inducible activation of PrfA. In support, the structural data demonstrate electrostatic interactions between residues Lys64 and Lys122 with the carboxylate moiety of inhibitor **1**, and van der Waal contacts between the aliphatic backbone of Tyr62 and the naphthyl ring of **1** (Figure 6D). Both Lys64 and Lys122 have previously been implicated as being involved in PrfA activity: K64Q or K122Q mutants exhibited reduced ActA expression, weakened capability to bind *hly*-DNA, and attenuated virulence (Xayarath et al., 2011). At site BII compound **1** binds in the vicinity of the flexible HTH motif (Figures S6B and S6C). At this site the naphthyl group of compound **1** sterically prevents restructuring of residues Phe131 and Phe134 into positions needed for formation of an HTH motif compatible with DNA binding (Figure S6D) (Levy et al., 2008, Schultz et al., 1991). One interesting observation from the in vitro experiments was the binding of **1–3** to both PrfA_WT_ and PrfA_G145S_ determined by ITC, despite the lack of efficacy against strains harboring the constitutively active G145S mutant (Table 1). Since the PrfA_G145S_ mutant that has structurally defined HTH motifs can still bind to its consensus DNA when in complex with **1** (Figure 5C), one possibility is that compound **1** only binds at the AI site in the PrfA_G145S_ mutant. Binding of **1** in the tunnel only of PrfA_G145S_ needs, however, to be structurally verified in future, and the similar binding efficacies of **1–3** in vitro suggest that additional complexities may emerge in the in vivo mechanism of PrfA inhibition.

It has previously been shown that ActA is important for bacterial uptake into epithelial cells including Caco-2 and Hela (Suarez et al., 2001). ActA has also been suggested to promote the initial contact between the bacteria and the cell by binding Heparan sulfate proteoglycan receptor, thereby allowing a stable contact between InlA or InlB with E-cadherin and Met, respectively (Garcia-Del Portillo and Pucciarelli, 2012). Thus, our results showing a decreased uptake of bacteria in the presence of compounds **1** and **2** is consistent with a reduced expression of ActA (Figures 1 and 2). Furthermore, while compounds **1** and **2** were effective at attenuating virulence when added prior to infection, only high concentrations of **2** reduced ActA expression post infection (Figure 2D, 100 versus 10 μM in Figure 1A). A reduced accessibility of the compounds for PrfA after bacterial internalization or increased compound turn-over may have contributed to this, or the compounds may act more effectively at the step prior to PrfA activation (Figure 2D). The mechanism of intracellular PrfA activation has been postulated to involve a small molecule cofactor (Deshayes et al., 2012) that induces the ordering of the HTH motif evident in the PrfA_G145S_ structure, thus enabling high-affinity interactions with consensus DNA sequences (Eiting et al., 2005). Recently it was shown that PrfA is more capable of initiating virulence gene expression if it is in a reduced state and bound to reduced glutathione (Reniere et al., 2015). Post infection, the inhibitory compounds are likely in competition with a cofactor, and PrfA activation may already have occurred, giving rise to a stable HTH motif. Consistent with this scenario is the reduced efficacy of compounds post infection (Figure 2D) and loss of efficacy against strains bearing the constitutively active PrfA_G145S_ (Figure 4). In addition, both PrfA_WT_- and PrfA_G145S_-expressing strains were able to replicate efficiently in J774.1 macrophage-like cells after treatment with **2** (Figure S7), in line with the finding that maximal PrfA activity is not required for *L. monocytogenes* replication in bone marrow-derived macrophages (Reniere et al., 2015). Although our body of evidence clearly shows that the 2-pyridones interact with and inactivate PrfA, we do not completely rule out the possibility that they might also affect additional components contributing to *L. monocytogenes* virulence.

Other Crp/Fnr family members, such as Crp in *E. coli*, utilize cAMP binding for activation (Won et al., 2009). While the cAMP-binding site is topologically conserved within the N-terminal domain of PrfA, the residues involved in cAMP binding are not (Eiting et al., 2005). The crystal structure of the PrfA_WT_-**1** complex shows that **1** does not bind at the conserved Crp cAMP-binding site (Figure S5B), nor to a solvent accessible cavity in the N-terminus of PrfA, recently proposed as a potential cofactor binding site (Deshayes et al., 2012). The described inhibitors are therefore unlikely to compete with a cofactor binding at those sites. The interdomain tunnel where **1** binds at site AI has previously been proposed as a possible effector site (Eiting et al., 2005), and our findings provide additional support for the importance of this region within an allosteric model of activation for PrfA. Further studies can clarify the relationship between glutathione as a substrate for activation and the discovered inhibitors.

Compound **1** came from a collection of molecules designed to block pilus assembly in *E. coli* through disrupting the chaperone-usher pathway (Emtenäs et al., 2002, Svensson et al., 2001). When used at a concentration of 3.6 mM, **1** blocked pilus biogenesis in *E. coli*, which raises the question of whether such effects may have been mediated through inhibition of Crp (Pinkner et al., 2006). The broad applicability of compounds based on **1** and **2** to inhibit other Crp/Fnr family members requires further investigation; however, several factors point against the favorability of these compounds functioning as broad spectrum Crp/Fnr family inhibitors. At concentrations much higher than applied here, **1** had no effect in *E. coli* on the Crp-regulated pili expression (at 800 μM) (Cegelski et al., 2009, Chorell et al., 2010). At the protein level, PrfA differs structurally from Crp by the presence of three extra C-terminal helices (αG-αI) at the AI site (Eiting et al., 2005) and the BII site with its HTH motif is too compact to allow binding of **1** in Crp. Finally, at the bacterial level, the Gram-negative pathogen *E. coli* and Gram-positive *L. monocytogenes* are topologically distinct, which can have a profound impact on uptake and accessibility for small molecule inhibitors. The present work, however, provides a compelling proof-of-concept for investigating small molecule inhibitors of Crp family members as a means to disrupt the virulence of pathogenic bacteria.

Antivirulence drugs could provide a powerful, and potentially complementary treatment option to conventional antibiotics. Inhibitors of virulence in pathogenic bacteria, rather than inhibitors of viability, are now emerging, but only a few examples have been reported where the key regulator of virulence has been directly inactivated and none have provided structural details of the regulator-compound interaction (Garrity-Ryan et al., 2010, Hung et al., 2005, Shakhnovich et al., 2007, Sun et al., 2011, Yang et al., 2013). In the present work, we have identified a class of ring-fused 2-pyridones which attenuate *L. monocytogenes* virulence through inhibiting PrfA activation. These inhibitors represent excellent tool compounds for studying the virulence of this potentially fatal pathogen. Furthermore, the elucidated crystal structure of PrfA in complex with compound **1** enables improved ligand design, and provides a compelling rationale for the further investigation of Crp family members with small molecule inhibitors. Translating this research into a clinical application requires more effective inhibitors and in vivo proof-of-concept, but these findings represent the first steps in this process of identifying new therapeutic opportunities to treat infectious diseases.

## Significance

**With increasing antibiotic resistance, new therapeutic strategies are needed to control bacterial infections, and virulence inhibition may afford opportunities for effective control. In this article, we have identified ring-fused 2-pyridones that attenuate the virulence of the Gram-positive bacterial pathogen *Listeria monocytogenes* by decreasing expression of central virulence genes. We identify the virulence regulator PrfA as the target of the compounds, and show how these inhibitors interact directly with PrfA to reduce its DNA-binding capacity. The identified inhibitors are a new set of tool compounds for improving our understanding of *L. monocytogenes* virulence. We present the first crystal structure of a Crp/Fnr family member in complex with an inhibitor, and this affords new opportunities for designing ligands targeting this widespread family of bacterial transcriptional regulators.**

## Experimental Procedures

### Chemistry

All tested compounds were of ≥95% purity as determined by liquid chromatography-mass spectrometry. Compound **1** was synthesized as described previously (Chorell et al., 2010). Compounds **2–5** were synthesized by Suzuki-Miyaura cross-coupling with the chloromethyl ester **6** and subsequently hydrolyzed (Figure S8) (Chorell et al., 2010, Sellstedt et al., 2012). Detailed procedures and characterization are provided in the Supplemental Experimental Procedures.

### Cell Lines, Bacterial Strains, and Growth Conditions

*L. monocytogenes* strains (EGDe, Mackaness, 1964; EGDe Δ*actA*, Tiensuu et al., 2013; EGDe Δ*prfA*, Toledo-Arana et al., 2009; EGDe Δ*hly*, Tiensuu et al., 2013; LO28, Vicente et al., 1989; 10403S, Myers et al., 1993; and F2365, Nelson et al., 2004) were grown in brain heart infusion (BHI) medium (Fluka) at 37°C with aeration (180 rpm), unless otherwise stated. For growth rate determination, samples were withdrawn every 30 min and optical density at 600 nm (OD_600_) was measured. When required, erythromycin was used at a final concentration of 10 μg/ml. *E. coli* (DH5α; Bethesda Research Laboratories, 1986) was grown in Luria-Bertani medium at 37°C with agitation, unless otherwise stated. Antibiotics were, where appropriate, supplemented to final concentrations of 34 μg/ml for chloramphenicol and 100 μg/ml for kanamycin. HeLa cells were maintained in RPMI 1640 medium, whereas Caco-2 and J774 cells were maintained in DMEM, both supplemented with GlutaMAX Supplement (Gibco, Life Technologies) and 10% fetal bovine serum (Gibco, Life Technologies) at 37°C in a 5% CO_2_ atmosphere.

### Infection

*L. monocytogenes* EGDe, grown to an OD_600_ of 1.0, was pelleted, washed, and diluted in RPMI. DMSO or compound dissolved in DMSO was then added to the bacteria, resulting in a final concentration of 0.5% DMSO (v/v). The bacterial suspensions were added to the mammalian cells at a multiplicity of infection of approximately 10 and incubated for 1 hr at 37°C, before the cells were washed in PBS and incubated for 1.5 hr in cell medium supplemented with 50 μg/ml gentamycin. The cells were washed in PBS and subsequently lysed with PBS supplemented with 1% Triton X-100. The amount of intracellular bacteria was quantified by viable count of the cell lysate. For flow cytometry: after infection as above, the cells were washed in PBS and incubated for 7 hr in RPMI supplemented with 50 μg/ml gentamycin and subsequently analyzed using the Guava easyCyte (Millipore) flow cytometer. For intracellular protein levels, see Supplemental Experimental Procedures.

### Western Blot

Overnight cultures of *L. monocytogenes* were diluted to an OD_600_ of 0.025 in BHI supplemented with DMSO, or the compounds dissolved in DMSO, resulting in a final concentration of 0.1% DMSO (v/v). The cultures were grown at 37°C to an OD_600_ of 1.0 before centrifugation (4,500 rpm, 10 min, 4°C). The supernatants and bacterial pellets were collected and subjected to SDS-PAGE and western blotting using specific antibodies. A detailed procedure is provided in the Supplemental Experimental Procedures.

### Surface Plasmon Resonance

SPR experiments was conducted in a Biacore X100 (GE Healthcare), and performed mainly as described previously (Deshayes et al., 2012). In brief, biotinylated oligonucleotides (Promo40plcA-P14/Promo40plcAREV) containing the PrfA box of the *plcA* promoter were immobilized on one flow cell of an SA Chip (GE Healthcare). Assays were performed at 37°C. Protein samples were diluted in HBS-EP+ (GE Healthcare) to a final concentration of 200 nM, and compounds at different concentrations were added, resulting in a final DMSO concentration of 0.5%. The samples were injected with a flow rate of 10 μl/min. HBS-EP+ was used as running buffer. Measurements were done with a contact time of 200 s and a dissociation time of 120 s. This was followed by regeneration solution (0.1% SDS, 3 mM EDTA) with a contact time of 60 s. A flow cell without any immobilized DNA was used for reference subtraction. Each run was repeated three times.

### Isothermal Titration Calorimetry

ITC experiments were performed using a MicroCal AutoiTC200 (GE Healthcare). In a typical run, 25 automated injections of 1.65 μl with 170-s breaks between injections were made at 25°C with 600 rpm stirring speed on low feedback mode. The protein concentrations in the sample cell were varied between 25 and 100 μM, while the compound concentration in the syringe was varied from 300 to 800 μM. The buffer for both protein and compound solutions was the same as in the final step of the purification. The compounds were dissolved in DMSO, and then diluted with buffer. DMSO content in the final compound solutions did not exceed 0.5% (v/v). Data integration, fitting, and evaluation were performed using the software Origin 7 with the ITC200 plugin provided by MicroCal/GE Healthcare.

### Protein:Compound Crystallization and Data Collection

PrfA in complex with **1** was co-crystallized by the hanging-drop vapor-diffusion technique at 18°C. Crystals (0.1 × 0.4 × 0.02 mm^3^) grew in 5 days when the protein solution (3.5 mg/ml PrfA, 0.75 mM **1**, 200 mM sodium chloride, 20 mM NaP [pH 6.5]) was mixed with an equal volume of mother liquor containing 20% PEG-4000, 16% isopropanol, and 100 mM sodium citrate (pH 5.5). Before data collection, the crystals were transferred to a cryo-protectant solution including 16% (v/v) glycerol in the precipitant solution. The crystals were flash-cooled to 100 K using a Cryostream 700 cooler (Oxford Cryosystems) and stored in liquid nitrogen. Diffraction data were collected at 100 K at the ESRF beamline ID29 (X-ray wavelength = 0.914 Å). The structure was solved with molecular-replacement methods. Data collection and refinement statistics are shown in Table 2. Details of the structure determination are provided in the Supplemental Information. The atomic coordinates and structure factors have been deposited (PDB: 5F1R) in the Research Collaboratory for Structural Bioinformatics (RCSB), Rutgers University, New Brunswick, NJ (http://www.rcsb.org/).

## Author Contributions

J.J., F.A., E.S.A., C.A., S.B., S.H., J.G., and P.W.S. wrote the manuscript. K.S.K., E.C., and J.G. synthesized and characterized the molecules. A.B., C.G., U.H.S., and E.S.E. determined the crystal structure and interpreted the data with J.J., F.A., and J.G. C.A., S.H., and J.W. performed western blot analysis and cell-infection experiments. J.W. and K.V. performed northern blot experiments. S.H. determined the hemolytic activity and growth rate. K.V. performed preliminary experiments. M.S.N. performed ITC and analyzed the data with P.W.S. C.A. performed and analyzed the SPR experiments. F.A. and J.J. conceived and initiated the project. All authors read and edited the manuscript.

## Figures and Tables

**Table 1:** Binding Parameters of **1**–**3** to PrfA_WT_ and PrfA_G145S_ as Determined by ITC

Protein	Compound	*K*_D_ (M)	Δ*H*_A_ (kcal/mol)	Δ*S*_A_ (cal/mol·K)
PrfA_WT_	**1**	1.0 (±0.2) × 10^−6^	−8.4 ± 0.1	−0.8 ± 0.1
**2**	6.7 × 10^−6^	−6.5	2.8
**3**	1.6 (±0.2) × 10^−6^	−8.4 ± 0.2	−1.5 ± 0.9
PrfA_G145S_	**1**	7.75 × 10^−6^	−6.2	2.6
**2**	5.4 × 10^−6^	−4.1	10.4
**3**	5.0 (±1.8) × 10^−6^	−6.2 ± 1.0	3.4 ± 4.1

Δ*H*_A_, enthalpy of association and Δ*S*_A_, entropy of association. Determined at 293 K. In all cases, 1 to 1 stoichiometry per PrfA monomer was observed. Errors refer to independent repetitions (2–3) of experiments. Fitting errors not shown. See also Figures 5 and S3.

**Table 2:** Data Collection and Refinement Statistics

	PrfA_WT_-**1**

**Data Collection**	

Space group	P21
Cell dimensions	
a, b, c (Å)	56.34, 80.97, 62.34
α, β, γ (°)	90.00, 112.54, 90.00
Resolution (Å)	57.58–2.25 (2.33–2.25)^a^
*R*_merge_	0.058 (0.985)
*R*_pim_	0.041 (0.698)
Wilson B factor (Å^2^)	60.3
<I/σI>	12.1 (1.6)
Completeness (%)	99.2 (97.9)
Redundancy	5.6 (5.5)
CC1/2^b^	2.25 Å

**Refinement**	

Resolution (Å)	57.58–2.25 (2.33–2.25)
Number of reflections	24,400 (2,375)
*R*_work_/*R*_free_	0.203/0.254 (0.370/0.400)
Number of atoms	
Protein	3,738
Ligand	54
Water	22
B factors (Å^2^)	
Protein	
Monomer A	86.6
Monomer B	83.6
Ligands	
**1** (AI)	65.5
**1** (BII)	70.1
Water	61.2
Root-mean-square deviation	
Bond lengths (Å)	0.016
Bond angles (°)	1.40
Ramachandran (%)	
Favored	98.0
Outliers	0.0
Clash score	3.21

a Data collected from one crystal.

b Suggested resolution cut-off (AIMLESS, see Supplemental Experimental Procedures). Values in parentheses are for highest-resolution shell.

## Supplemental Information

Document S1. Supplemental Experimental Procedures and Figures S1–S8

Document S2. Article plus Supplemental Information
