# Supplementary material for: Attenuating Listeria monocytogenes Virulence by Targeting the Regulatory Protein PrfA
Source: Cell Chem Biol. 2016 Mar 17;23(3):404–14. doi: 10.1016/j.chembiol.2016.02.013 (PMC4802734; doi:10.1016/j.chembiol.2016.02.013)
Supplement: Document S1. Supplemental Experimental Procedures and Figures S1–S8 [file mmc1.pdf]

Cell Chemical Biology, Volume *23*

Supplemental Information

Attenuating *Listeria monocytogenes* Virulence by Targeting the Regulatory Protein PrfA

James A.D. Good, Christopher Andersson, Sabine Hansen, Jessica Wall, K. Syam Krishnan, Afshan Begum, Christin Grundström, Moritz S. Niemiec, Karolis Vaitkevicius, Erik Chorell, Pernilla Wittung-Stafshede, Uwe H. Sauer, A. Elisabeth Sauer-Eriksson, Fredrik Almqvist, and Jörgen Johansson

Supplemental Information for:

Attenuating *Listeria monocytogenes* virulence by targeting the regulatory protein PrfA

James A. D. Good[a,b,1], Christopher Andersson[b,c,d,1], Sabine Hansen[b,c,d,1], Jessica Wall[b,c,d,1], K. Syam Krishnan[a,b,1], Afshan Begum[a,b], Christin Grundström[a,b], Moritz S. Niemiec[a], Karolis Vaitkevicius[b,c,d], Erik Chorell[a,b], Pernilla Wittung-Stafshede[a], Uwe H. Sauer[a,b], A. Elisabeth Sauer–Eriksson[a,b,2], Fredrik Almqvist[a,b,2] and Jörgen Johansson[b,c,d,2]

[a]Department of Chemistry, [b]Umeå Centre for Microbial Research (UCMR), [c]Department of Molecular Biology, [d]Molecular Infection Medicine, Sweden (MIMS),
Umeå University, 901 87 Umeå, Sweden.

[1]These authors contributed equally

[2]Corresponding authors: Jörgen Johansson: +46907852535, E-mail: jorgen.johansson@umu.se; Fredrik Almqvist: +46907866925, E-mail: Fredrik.almqvist@umu.se; Elisabeth Sauer-Eriksson: +46907865923, E-mail: Elisabeth.sauer-eriksson@umu.se

Present address: K.S.K: Department of Chemistry, Mannam Memorial NSS College, Kottiyam, Kollam, Kerala, India-691571; P.W.S.: Department of Biology and Bioengineering, Chalmers University of Technology, 41296 Gothenburg, Sweden.

Figure S1

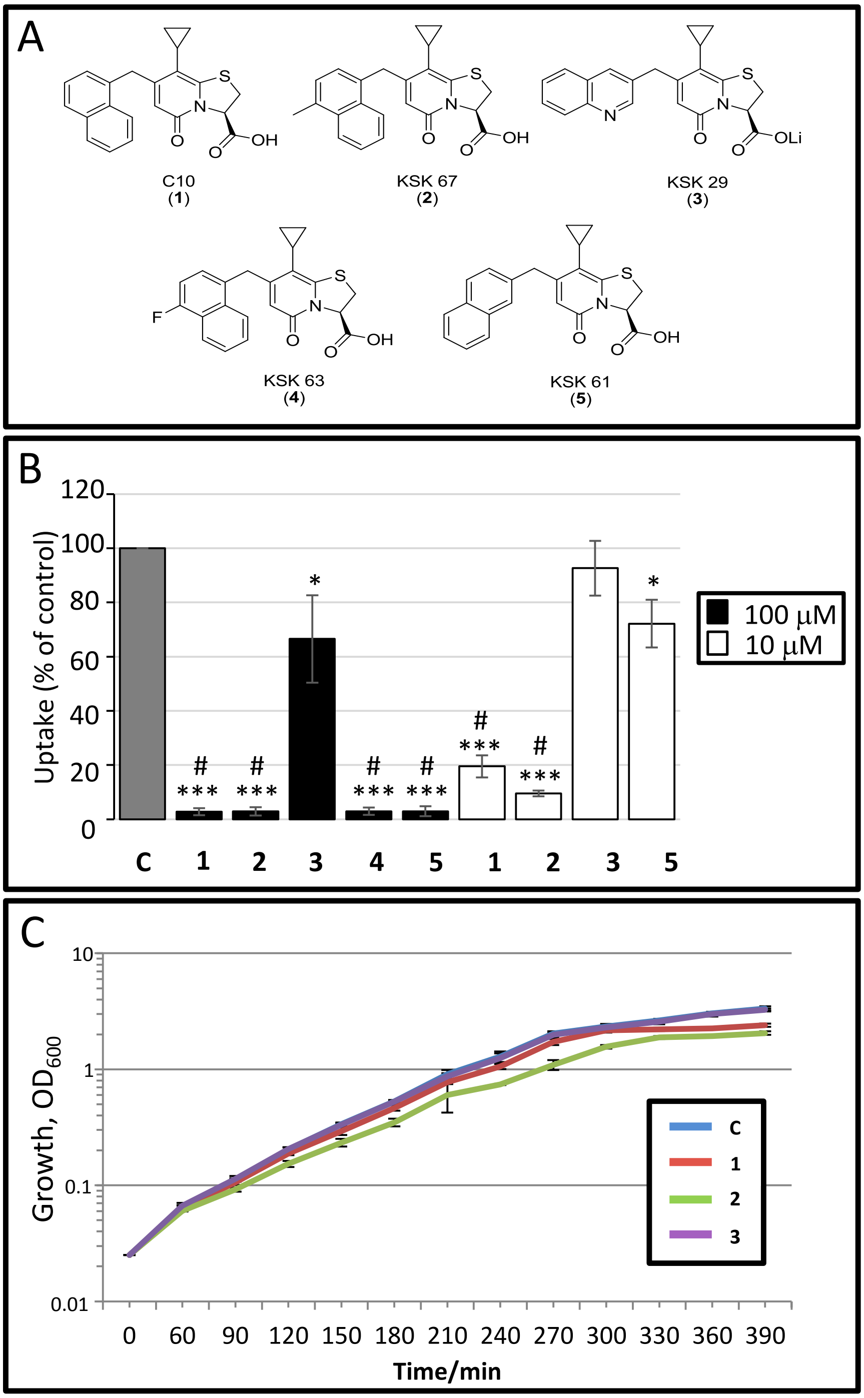

Figure S1. Infection inhibition and effect on growth by ring-fused 2-pyridines related to Figure 1. (A) Structures of bicyclic 2-pyridones used in this study. (B) Effect of bicyclic 2-pyridones on *L. monocytogenes* infectivity. GFP-expressing *L. monocytogenes* treated with compound at the indicated concentrations (100 µM, black bars; 10 µM, white bars) was allowed to infect HeLa cells for 7 hours, before analysis with a flow cytometer. The relative infection is calculated compared to the control (C; DMSO treated *L. monocytogenes*) (100%, grey bars). Values represent the mean ± standard deviation. n = 3. Significance was tested using Student's t-test (two-tailed - significant differences are shown by asterisks; * p<0.05 and *** p<0.001) and Dunnett's test (significant differences to the control are shown by #). (C) Growth of *L. monocytogenes* EGDe in brain heart infusion (BHI) medium + 0.1% DMSO (C - solvent); BHI + 100 µM **1**; **2** or **3**, respectively. Growth (n=4) was plotted as growth (OD_{600}) versus time (minutes). Error bars show standard deviation. The difference in growth between strains treated with compounds **1** or **2** compared with DMSO is statistically significant according to Student's t-test.

Figure S2

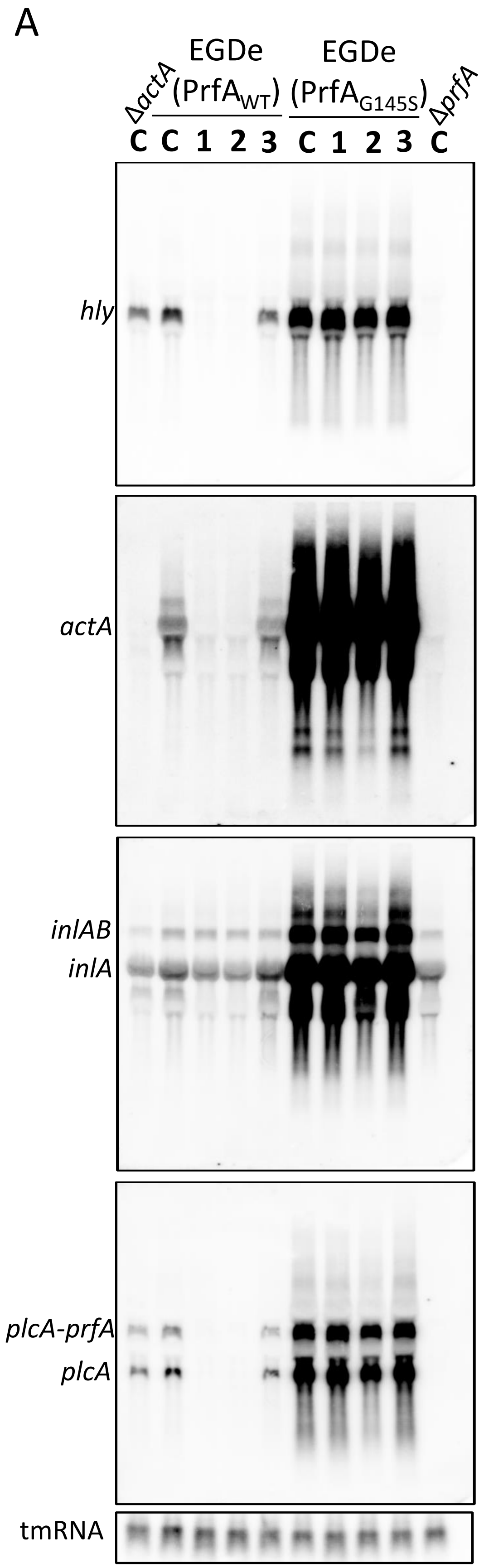

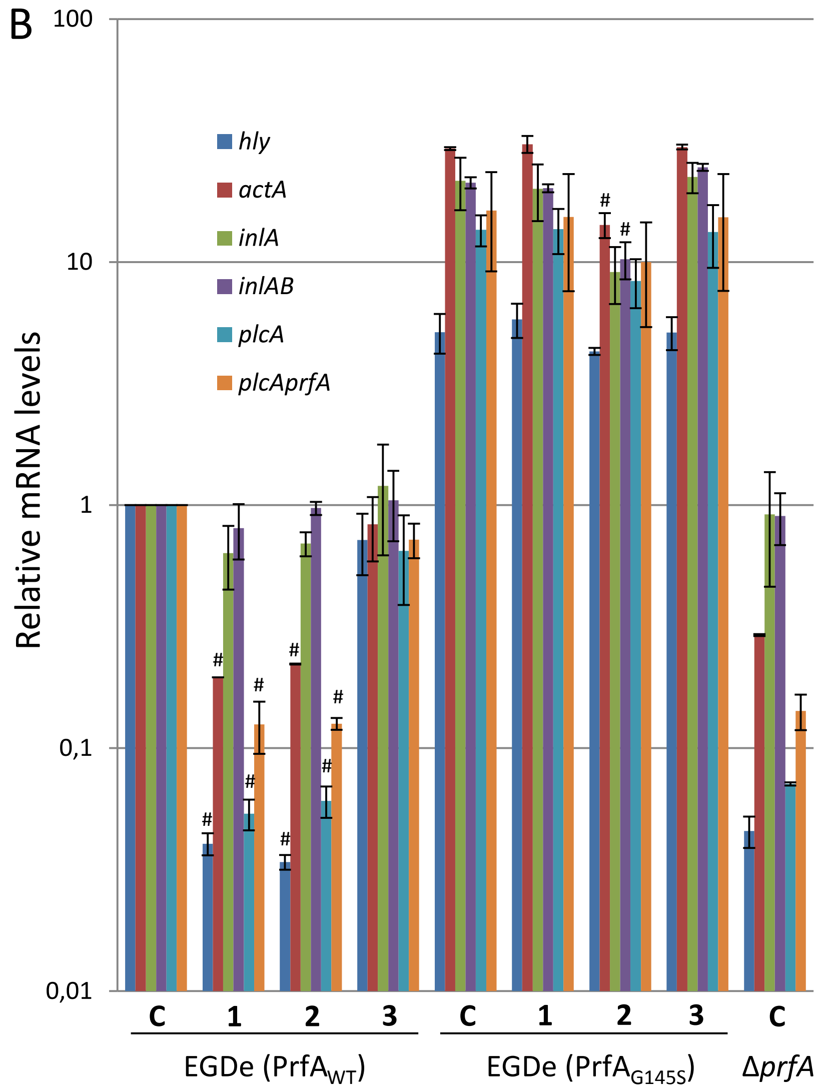

Figure S2. Effect of compounds on *L. monocytogenes* virulence gene expression related to Figure 2. (A) Total RNA was isolated from the indicated strains (Δ*actA*; EGDe (PrfA$_{WT}$); EGDe (PrfA$_{G145S}$) and Δ*prfA*, respectively) grown in BHI medium supplemented with DMSO (C) or compounds **1, 2** or **3** at 100 µM. Northern blot analysis was performed and membranes were hybridized with *hly*, *actA*, *inlA*, *plcA* or tmRNA specific DNA probes. Specific products are indicated at the left of the blots. (B) Relative levels of specific mRNA products identified in (a). All values were divided by the value of the wild-type strain (EGDe (PrfA$_{WT}$)) treated with DMSO, which was arbitrarily set to 1.0. Error bars show standard deviation. Significance was tested using Dunnett's test (significant differences to the control are shown by #). Values for samples from EGDe (PrfA$_{WT}$) treated strains were compared with the value of sample (EGDe (PrfA$_{WT}$) treated with DMSO, whereas samples from EGDe (PrfA$_{G145S}$) treated strains were compared with the value of sample (EGDe (PrfA$_{G145S}$) treated with DMSO.

Figure S3

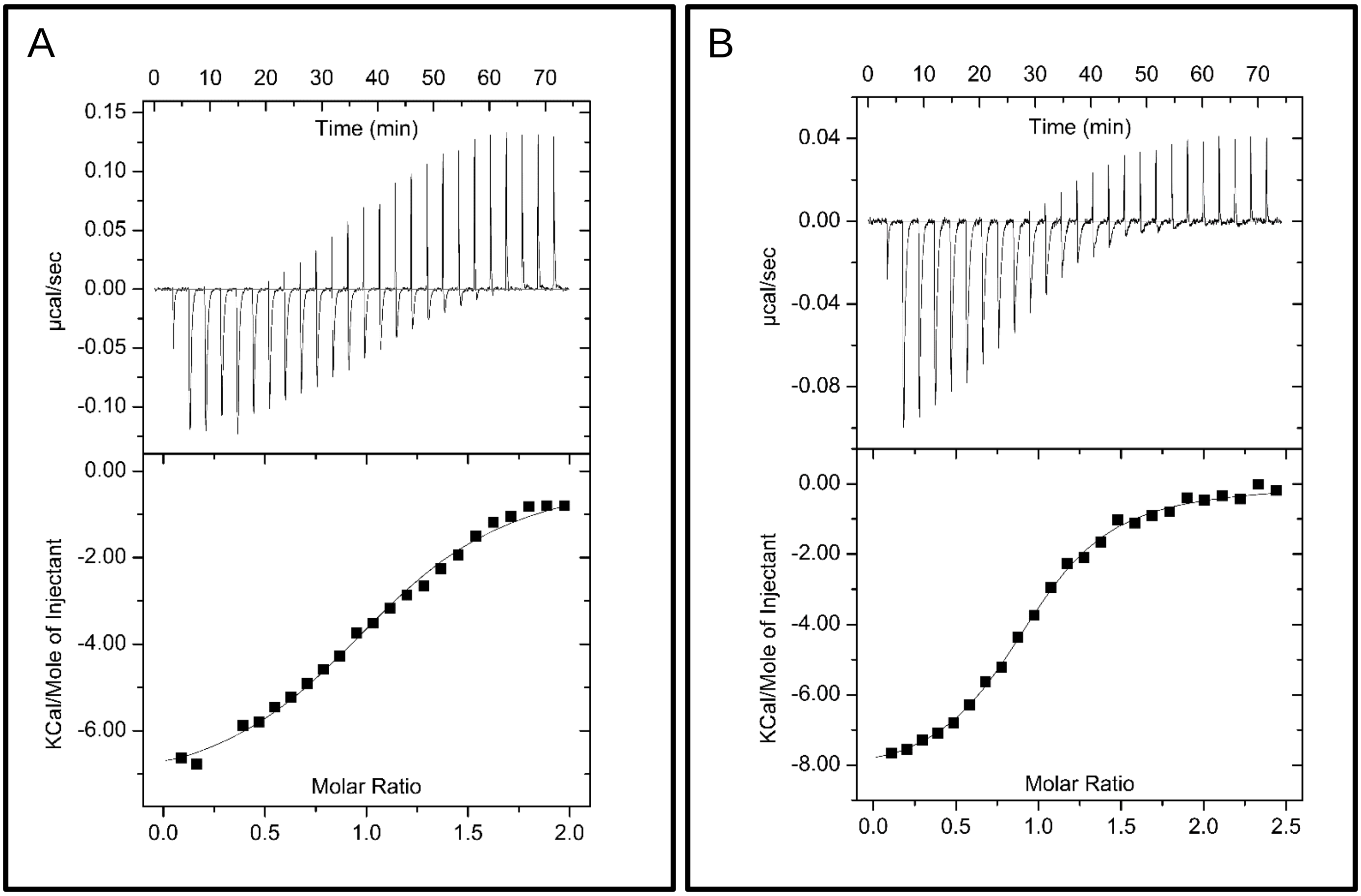

Figure S3. Isothermal titration calorimetry of compounds 2 (A) and 3 (B) to PrfA$_{WT}$, related to Figure 5. In each case small volumes of compound were injected to a solution of PrfA in the cell. The upper parts of the graphs show the measured heat after every injection, while the lower parts show integrated heat, normalized by amount and after background subtraction, as a function of molar ratio of compound to PrfA. Least-square fitting yields thermodynamic parameters that are given in Table 1.

Figure S4

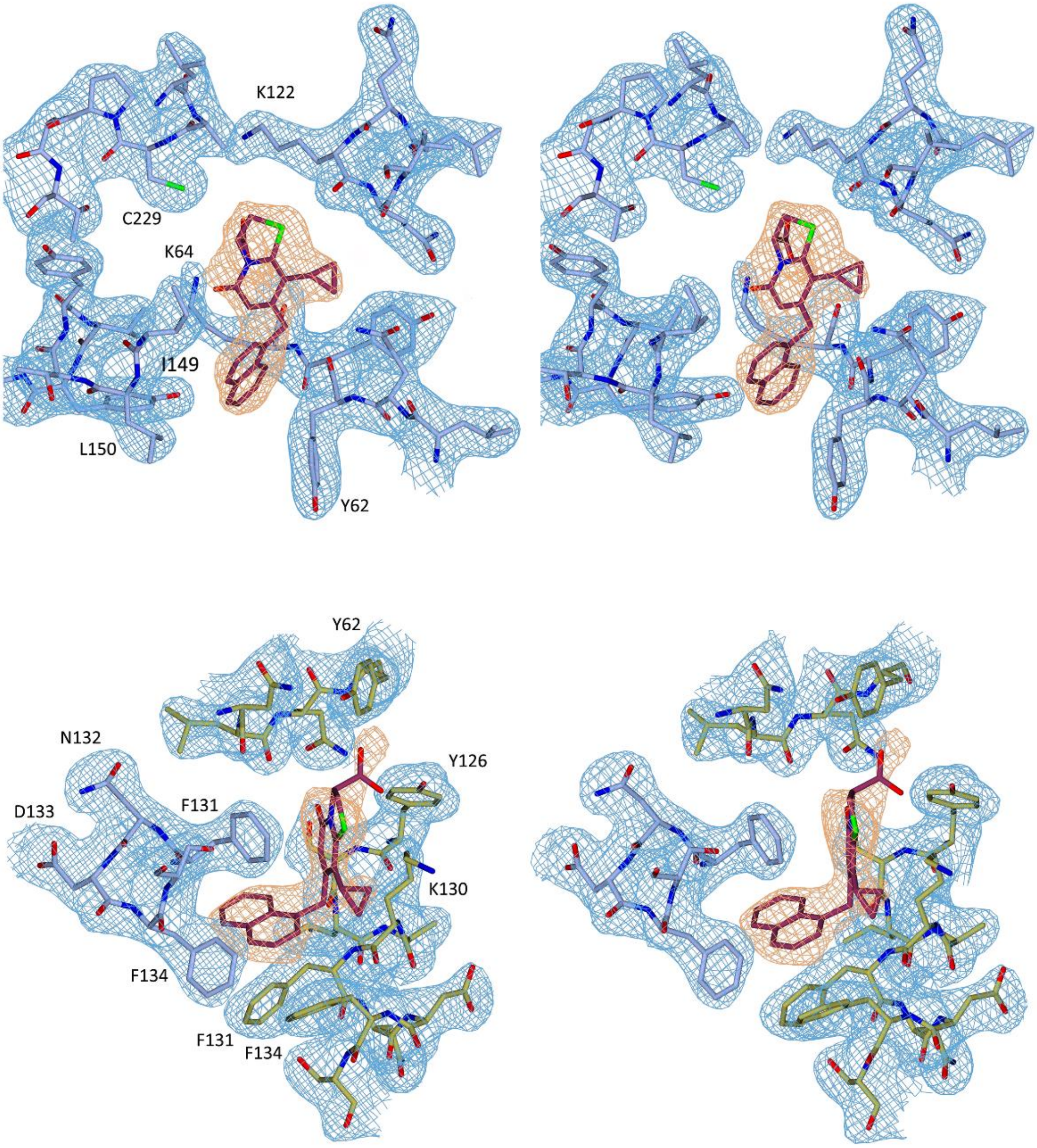

Figure S4. Validation of ligand binding, related to Figure 6. Related to Experimental Procedures. Upper panel: Stereo image showing the electron density quality of **1** bound to monomer A at site AI of the PrfA dimer. Lower panel: Stereo image showing the electron density quality of **1** bound to monomer B at site BII of the PrfA dimer. The σ^A-weighted (2m|Fo|-D|Fc|) electron density map calculated with phases from the refined structure is contoured at one root-mean-square deviation (RMSD) value of the map, and shown in blue. The σ^A-weighted (m|Fo|-D|Fc|) electron density, contoured at 3 times the RMSD value is shown in orange. To avoid model bias compound **1** was excluded from the coordinate file that was subjected to one round of refinement before calculation of the |Fo|-|Fc| electron density map. Shown is the electron density covering **1** and selected residues of the binding sites including amino acids A-Leu60-Lys64, A-Leu120-Val124, A-Ile149-Gly155 and A-Leu227-Thr232 at site AI and amino acids A-Phe131-Ser135, B-Asn59-Tyr62, and B-Tyr126-Ser135 at site BII. In both binding sites compound 1 is well defined in the non-biased (m|Fo|-D|Fc|) electron density map.

Figure S5

A

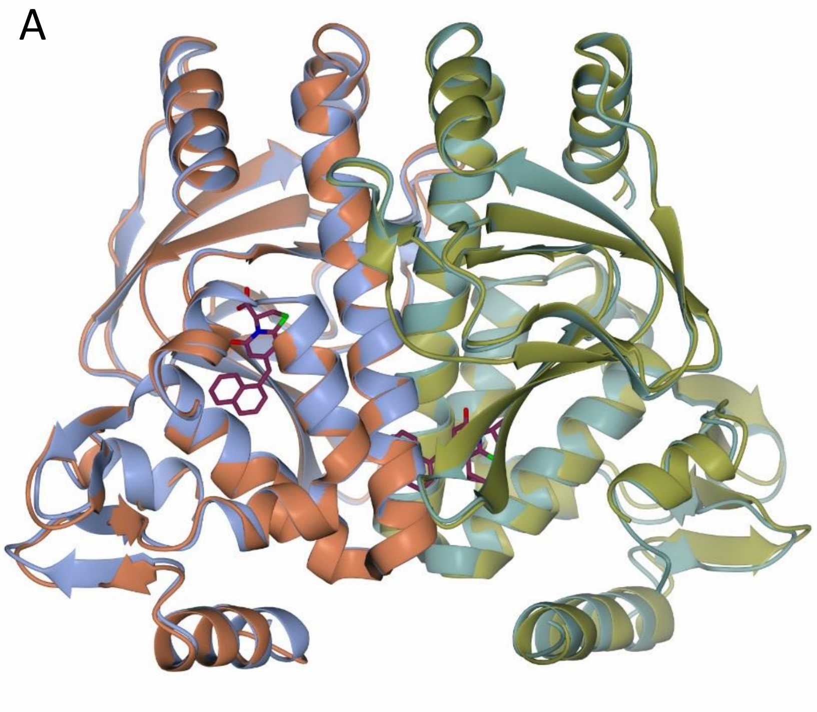

Figure S5. PrfA$_{WT}$ and PrfA$_{WT}$:1 complex structures are similar and compound 1 and cAMP occupy different binding sites, related to Figure 6. (A) Superposition of monomer A of the PrfA$_{WT}$:**1** complex structure (monomer A, blue; monomer B, gold) onto monomer A of the PrfA$_{WT}$ structure (monomer A, orange; monomer B, sea-green, PDB entry 2beo, (Eiting et al., 2005)) shows that their 3D structures are similar. Superimposition of the two structures results in an RMSD of 0.9-1.0 Å. This demonstrates that binding of **1** to PrfA$_{WT}$ has a negligible effect on the overall structure of the dimer. The ligands are colored by atom type: purple (C), green (S), blue (N) and red (O). (B) Superposition of the PrfA:1 (blue and green) and Crp:cAMP complexes (gray, pdb-code 1g6n, (Passner et al., 2000)) based on monomer A only. The closest distances between cAMP and compound 1 at sites AI and BII are ~4 Å and ~5 Å, respectively.

B

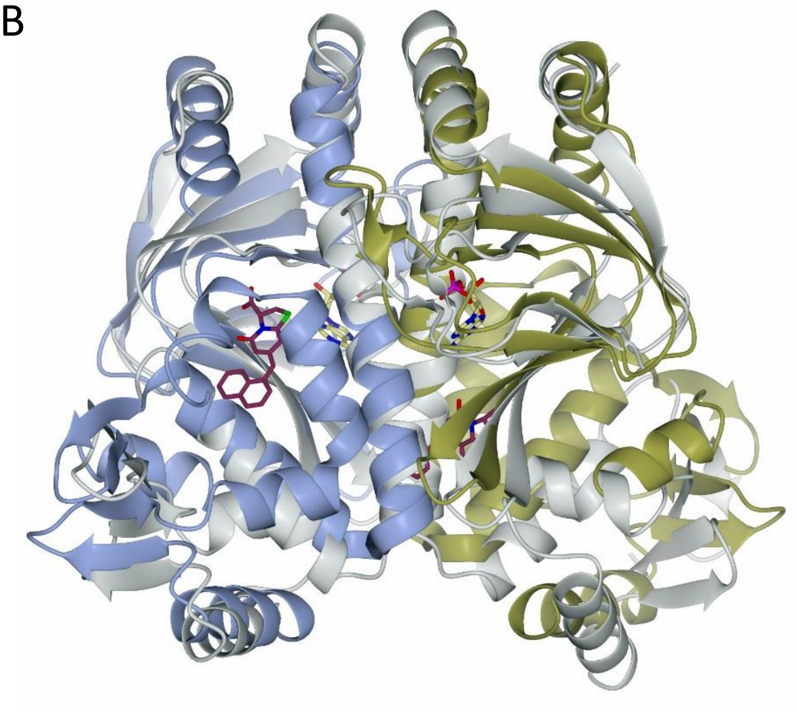

Figure S6

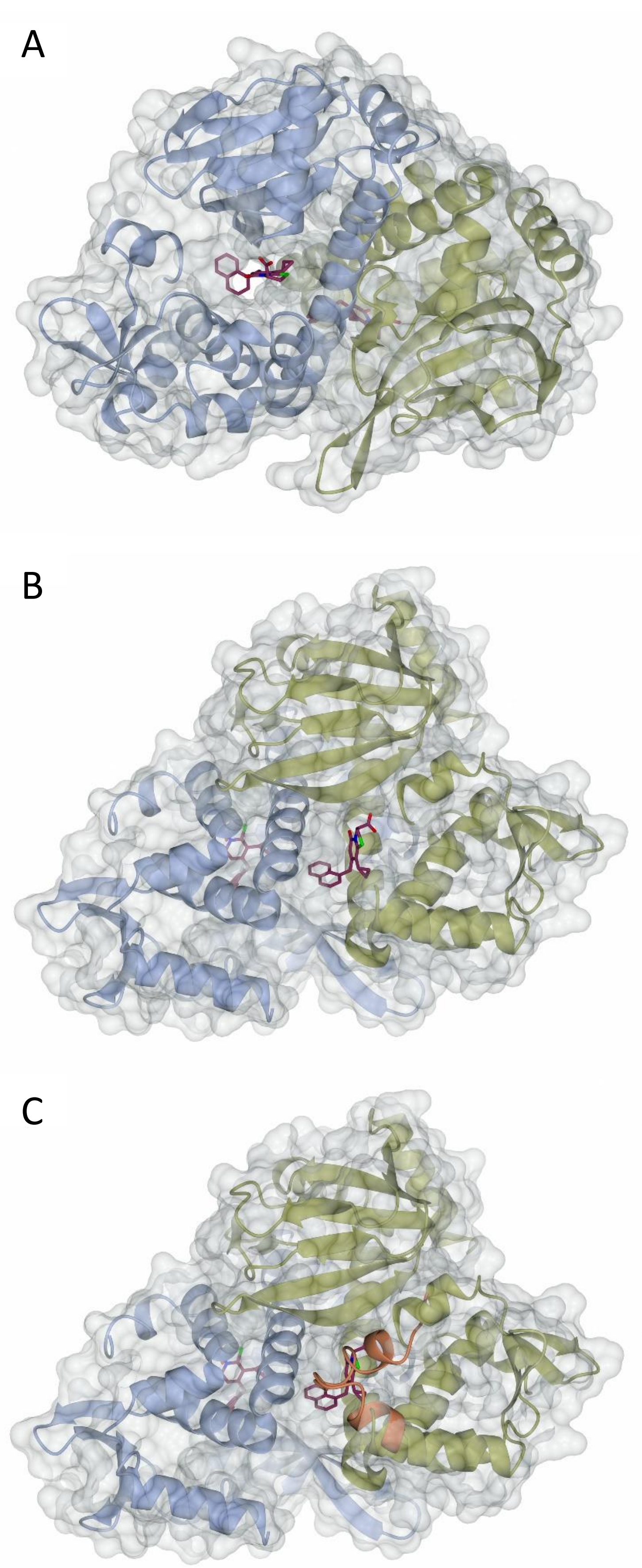

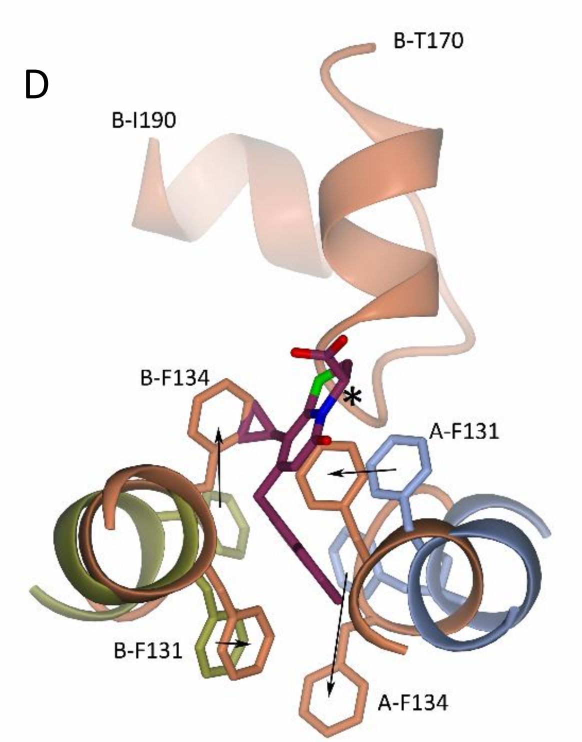

Figure S6. Compound 1 binds at two areas of PrfA, related to Figure 6. (A) Binding within the interdomain tunnel in between helices αC, αD, αH, αI and β-strand β5 (site AI). (B) Binding of **1** at a pocket in between helices αC, αD, and β5 and in vicinity of the in the wild-type structure not folded HTH motif (site BII). (C) The same figure as in b including the HTH motif (residues 168-190, orange ribbon) from the superimposed $PrfA_{G145S}$ structure (pdb code 2bgc). (D) Close-up view of the BII site. Superimposed residues from the $PrfA_{G145S}$ structure are shown in orange. The naphthyl group of compound 1 is sandwiched between residues Phe131 and Phe134 from both monomers. This position of the naphthyl group prevents conformational changes needed for HTH formation. Furthermore, residues in the turn of the HTH motif, highlighted here with *, are positioned in close vicinity, 2.5-3.0 Å, from the 2-pyridone ring.

Figure S7

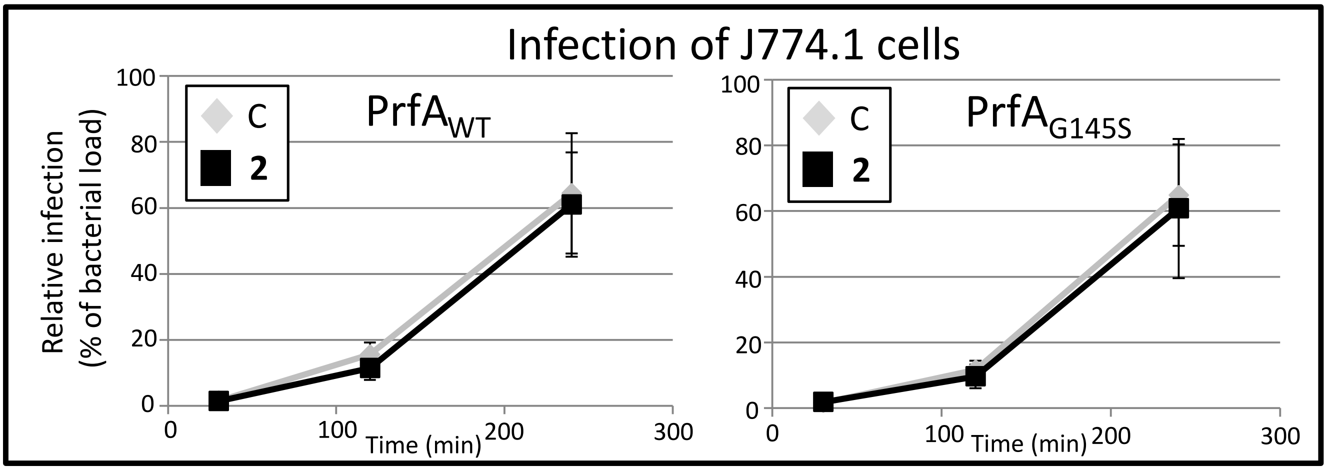

Figure S7. Time-course infection dynamics of *L. monocytogenes* harboring PrfA$_{WT}$ or PrfA$_{G145}$ after treatment with compound 2, related to Figure 1. J774.1 cells were infected with *L. monocytogenes* strains carrying PrfA$_{WT}$ (left panel) or PrfA$_{G145S}$ (right panel) in the presence of DMSO (C) or compound **2** (50 µM). The amount of intracellular bacteria were measured using viable count at indicated time-points post infection (p.i.) and divided by the bacterial load used in the infection (prior to antibiotic treatment) and set as %. n=3. Significance was tested using Student's t-test and Dunnett's test (No difference was significant.)

Figure S8

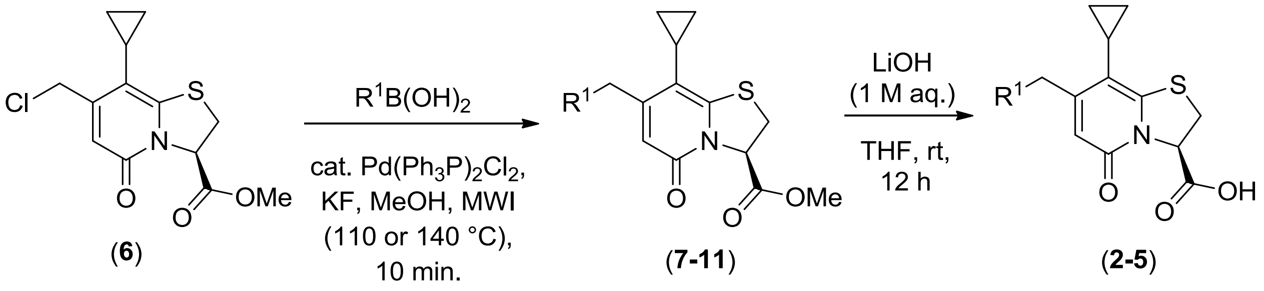

Figure S8. Synthesis of R-7 substituted thiazolino 2-pyridone derivatives, related to Figure 1.

Supplemental Experimental Procedures

Chemistry

General

All reagents and solvents were used as received from commercial suppliers. TLC was performed on aluminum backed silica gel plates (median pore size 60 Å) and detected with UV light at 254 nm. Column chromatography was performed using silica gel with average particle diameter 50 µM (range 40-65 µM, pore diameter 53 Å) and eluents are given in brackets. Optical rotation was measured with a polarimeter at 25 °C at 589 nm. IR spectra were recorded on a spectrometer equipped with an ATR device. ^{1}H and ^{13}C NMR spectra were recorded on a 400 MHz spectrometer at 298 K and calibrated by using the residual peak of the solvent as the internal standard ($CDCl_3$: δ_H = 7.26 ppm; δ_C = 77.16 ppm; DMSO-d_6: δ_H = 2.50 ppm; δ_C = 39.50 ppm). HRMS was performed by using a mass spectrometer with ESI-TOF (ESI+); sodium formate was used as the calibration chemical. HPLC purifications were performed on a system equipped with a 250 × 21.5 mm Nucleodur® C18 HTEC (particle size 5 µM) column using a flow rate of 20 mL/min and detection at 220 nm. Compound **1** was synthesized as described previously (Chorell et al., 2010).

General procedure for Suzuki-Miyaura cross-couplings

The chloromethyl derivative **6** (1.0 eq.), boronic acid (2.5 eq.), $Pd(Ph_3P)_2Cl_2$ (0.1 eq.) and KF (2.5 eq.) were dissolved in MeOH (10 mL) and heated by microwave irradiation (MWI) at 110 or 140 °C for 10 min. The reaction mixture was diluted with saturated aqueous $NaHCO_3$ and extracted with ethyl acetate. The organic layer was successively washed with water and brine,

dried (Na_2SO_4) and concentrated under reduced pressure. Purification by flash chromatography (SiO_2; EtOAc/heptane; 30-100%) afforded the cross-coupled product.

General methyl ester hydrolysis procedure

Methyl carboxylates **7-10** were hydrolysed by adaption of a previously reported procedure (Chorell et al., 2010). LiOH (1 M aqueous) was added drop wise to a stirred solution of methyl ester substituted 2-pyridone in THF (30 mL/mmol) at 0 °C. The solution was allowed to attain room temperature and was stirred for 12 h. The mixture was diluted with CH_2Cl_2 and acidified with aqueous HCl (1 M) to *circa* pH 1. The separated organic layer was dried (Na_2SO_4), and concentrated under reduced pressure. The residue was triturated with diethyl ether (3×) and lyophilized (H_2O:MeCN; ~ 8:2) to afford the carboxylic acid.

Methyl (3*R*)-8-cyclopropyl-7-[(4-methylnaphthalen-1-yl)methyl]-5-oxo-2,3-dihydro-5*H*-[1,3]thiazolo[3,2-*a*]pyridine-3-carboxylate (7). Following the general procedure with **6** (138 mg, 0.46 mmol) and 4-methyl-1-naphthaleneboronic acid (128 mg, 0.69 mmol) and MWI at 140 °C gave **7** (153 mg, 82%) as a white solid. ^{1}H NMR ($CDCl_3$, 400 MHz) δ = 8.01 (d, 1H, *J* = 7.6 Hz), 7.77 (d, 1H, *J* = 7.9 Hz), 7.59-7.52 (m, 2H), 7.33 (d, 1H, *J* = 7.2 Hz), 7.25 (d, 1H, *J* = 7.1 Hz), 6.00 (s, 1H), 5.54 (dd, 2H, J = 2.2, 8.6 Hz), 4.42 (d, 1H, *J* = 17.2 Hz), 4.32 (d, 1H, *J* = 17.2 Hz), 3.94 (s, 3H), 3.62 (dd, 2H, J = 8.6, 11.6), 3.50 (dd, 1H, *J* = 2.2, 11.6 Hz), 2.71 (s, 3H), 1.45 -1.35 (m, 1H), 0.97-0.87 (m, 2H), 0.70-0.61 (m, 2H); ^{13}C NMR ($CDCl_3$, 100 MHz) δ = 170.6, 160.1, 156.9, 148.0, 133.7, 133.1, 133.0, 132.2, 127.6, 126.4, 126.3, 126.2, 125.4, 125.1, 113.5, 112.6, 62.8, 55.3, 39.2, 31.5, 20.3, 11.3, 8.0, 7.7. HRMS (ESI+) (*m/z*): $[M]^+$ calcd. for $C_{24}H_{24}NO_3S^+$, 406.1471; found, 406.1460.

Methyl (3*R*)-8-cyclopropyl-5-oxo-7-(quinolin-3-ylmethyl)-2,3-dihydro-5*H*-[1,3]thiazolo[3,2-*a*]pyridine-3-carboxylate (8). The chloromethyl derivative **6** (175 mg, 0.58 mmol), 3-quinolineboronic acid pinacol ester (298 mg, 1.17 mmol), $PdCl_2(PPh_3)_2$ (21 mg, 0.03 mmol) and KF (68 mg, 1.17 mmol) were dissolved in anhydrous MeOH/MeCN (3.5 mL; 1:5) and heated by MWI at 110 °C for 10 min. The reaction mixture was quenched with saturated aqueous $NaHCO_3$ solution (10 mL) and extracted with EtOAc (3 × 10 mL). The combined organic extracts were washed successively with saturated aqueous $NaHCO_3$ solution, water (2×) and brine (10 mL each), dried (Na_2SO_4) and concentrated under reduced pressure. Purification by flash chromatography (SiO_2; MeOH/CH_2Cl_2; 0-20%) afforded the product as a pale yellow solid (174 mg, 76%). ^{1}H NMR ($CDCl_3$, 400 MHz) δ = 8.79 (d, 1H, *J* = 2.0 Hz), 8.11 (s, 1H), 8.02 (d, 1H, *J* = 8.0 Hz), 7.98 (d, 1H, *J* = 8.2 Hz), 7.72 (t, 1 H, *J* = 7.4 Hz), 7.58 (t, 1H, *J* = 7.4 Hz), 6.01 (s, 1H), 5.55-5.50 (m, 1H), 4.18 (d, 1H, *J* = 15.6 Hz), 4.12 (d, 1H, *J* = 15.6 Hz), 3.52-3.47 (m, 2H), 1.43-1.33 (m, 1H), 0.95-0.87 (m, 2H), 0.63-0.51 (m, 2H). ^{13}C NMR ($CDCl_3$, 100 MHz) δ = 170.3, 160.9, 154.3, 152.8, 149.9, 146.8, 135.6, 132.9, 129.9, 129.3, 128.2, 128.1, 127.2, 115.2, 111.9, 66.6, 56.1, 39.1, 31.6, 11.4, 8.4, 7.9. HRMS (ESI+) (*m/z*): $[M]^+$ calcd. for $C_{22}H_{21}N_2O_3S^+$, 393.1267; found, 393.1260.

Methyl (3*R*)-8-cyclopropyl-7-[(4-fluoronaphthalen-1-yl)methyl]-5-oxo-2,3-dihydro-5*H*-[1,3]thiazolo[3,2-*a*]pyridine-3-carboxylate (9). Following the general procedure, **6** (540 mg, 1.8 mmol, MWI at 140 °C) gave **9** (540 mg, 73%) as pale yellow solid. ^{1}H NMR ($CDCl_3$, 400 MHz) δ = 8.12-8.09 (m, 1H), 7.94-7.91 (m, 1H), 7.68-7.64 (m, 2H), 7.39-7.34 (m, 2H), 5.99 (s, 1H), 5.56 (dd, 1H, *J* = 2.6, 9.2 Hz), 4.44 (d, 1H, *J* = 17.6 Hz), 4.34 (d, 1H, *J* = 17.6 Hz), 3.95 (s, 3H), 3.74 (dd, 1H, *J* = 9.2, 11.6 Hz), 3.53 (dd, 1H, *J* = 2.6, 11.6 Hz), 1.49 -1.41 (m, 1H),

0.92-0.87 (m, 2H), 0.74-0.64 (m, 2H). ^{13}C NMR ($CDCl_3$, 100 MHz) δ = 169.9, 159.8, 157.3 (d, J_{CF} = 248 Hz), 155.9, 148.3, 132.8 (d, J_{CF} = 5.2 Hz), 130.9 (d, J_{CF} = 4 Hz), 127.8, 127.4, 126.5, 124.6, 123.0 (d, J_{CF} = 16.3 Hz), 120.5 (d, J_{CF} = 5 Hz), 113.6, 111.9, 109.2 (d, J_{CF} = 20 Hz), 63.3, 55.9, 39.0, 31.9, 10.7, 7.8, 7.3. HRMS (ESI+) (*m/z*): $[M]^+$ calcd. for $C_{23}H_{21}FNO_3S^+$, 410.1221; found, 410.1204.

Methyl (3*R*)-8-cyclopropyl-7-(naphthalen-2-ylmethyl)-5-oxo-2,3-dihydro-5*H*-[1,3]thiazolo[3,2-*a*]pyridine-3-carboxylate (10). Following the general procedure, **6** (360 mg, 1.2 mmol, MWI at 110 °C) gave **10** (358 mg, 76%) as pale yellow solid. ^{1}H NMR ($CDCl_3$, 400 MHz) δ = 8.11-8.08 (m, 1H), 7.98-7.95 (m, 1H), 7.69-7.65 (m, 2H), 7.53 (s, 1H), 7.37-7.32 (m, 2H), 6.06 (s, 1H), 5.56 (dd, 1H, *J* = 2.2, 9.2 Hz), 4.44 (d, 1H, *J* = 17.4 Hz), 4.33 (d, 1H, *J* = 17.4 Hz), 3.89 (s, 3H), 3.61 (dd, 1H, *J* = 9.2, 11.6 Hz), 3.46 (dd, 1H, *J* = 2.2, 11.6 Hz), 1.46 -1.37 (m, 1H), 0.93-0.80 (m, 2H), 0.77-0.59 (m, 2H). ^{13}C NMR ($CDCl_3$, 100 MHz) δ = 168.7, 161.3, 157.5, 157.0, 148.4, 133.7, 133.0, 132.1, 129.1, 128.8, 127.5, 127.1, 119.2, 115.7, 113.4, 107.8, 62.7, 55.3, 39.3, 31.9, 11.2, 7.9, 7.7. HRMS (ESI+) (*m/z*): $[M]^+$ calcd. for $C_{23}H_{22}NO_3S^+$, 392.1315; found, 392.1304.

(3*R*)-8-Cyclopropyl-7-(4-methyl-naphthalen-1-ylmethyl)-5-oxo-2,3-dihydro-5*H*-thiazolo[3,2-*a*]pyridine-3-carboxylic acid (2). Following the general procedure for hydrolysis with **7** (45 mg, 0.11 mmol) gave **2** (33 mg, 76%) as a white solid. $[\alpha]_D$ **=** -16 (*c* = 0.5, DMSO). IR (υ cm^{-1}) (neat) 1725, 1619, 1487, 1391, 1169. ^{1}H NMR (DMSO-d_6, 400 MHz) δ = 8.05 (d, 1H, *J* = 7.6 Hz), 7.86 (d, 1H, *J* = 8.0 Hz), 7.58-7.50 (m, 2H), 7.33 (d, 1H, *J* = 7.2 Hz), 7.25 (d, 1H, *J* = 7.2 Hz), 5.21-5.19 (m, 2H), 4.43 (d, 1H, *J* = 17.2 Hz), 4.33 (d, 1H, *J* = 17.2 Hz), 3.68-3.62 (m, 1H), 3.50 (d, 1H, *J* = 11.2 Hz), 2.66 (s, 3H), 1.72 -1.67 (m, 1H), 0.93-

0.85 (m, 2H), 0.74-0.70 (m, 1H), 0.65-0.60 (m, 1H). ^{13}C NMR (DMSO-d_6, 100 MHz) δ = 170.1, 160.4, 157.1, 148.4, 133.7, 133.1, 133.0, 132.1, 127.8, 126.8, 126.5, 126.2, 125.3, 125.1, 113.9, 112.4, 62.8, 35.8, 31.7, 19.6, 11.2, 7.9, 7.7. HRMS (ESI+) *m/z*: $[M+Na]^+$ calcd. for $C_{23}H_{21}NO_3S$, 414.1140; found, 414.1126.

Lithium (3*R*)-8-cyclopropyl-7-(isoquinolin-3-ylmethyl)-5-oxo-2,3-dihydro-5*H*-[1,3]thiazolo[3,2-*a*]pyridine-3-carboxylate (3). Aqueous LiOH (0.1 M, 0.71 mL, 0.071 mmol) was added drop wise to a stirred solution of methyl ester **8** (28 mg, 0.071 mmol) in THF (3 mL) at 0 °C. The solution was allowed to attain room temperature and stirred for 12 h, concentrated and purified by HPLC [mobile phase: MeCN/H_2O, (10-60 % for 30 min); t_R = 11.98 min]. Subsequent lypholization of the collected fractions (H_2O:MeCN; ~ 8:2) gave the lithium carboxylate **3** (20 mg, 73 %) as a pale yellow solid. $[\alpha]_D$ = -3 (*c* = 0.1, MeOH). IR (υ cm^{-1}) (neat) 1624, 1557, 1487, 1397, 1291, 1175. ^{1}H NMR (DMSO-d_6, 400 MHz) δ = 8.81 (d, 1H, *J* = 2.0 Hz), 8.15 (s, 1H), 8.00 (d, 1H, *J* = 8.4 Hz), 7.94 (d, 1H, *J* = 8.0 Hz), 7.72 (t, 1 H, *J* = 7.4 Hz), 7.58 (t, 1H, *J* = 7.4 Hz), 5.70 (s, 1H), 5.05-5.02 (m, 1H), 4.19 (d, 1H, *J* = 15.6 Hz), 4.13 (d, 1H, *J* = 15.6 Hz), 3.52 (d, 2H, *J* = 4.8 Hz), 1.40 -1.33 (m, 1H), 0.95-0.88 (m, 1H), 0.83-0.78 (m, 1H), 0.63-0.51 (m, 2H). ^{13}C NMR (DMSO-d_6, 100 MHz) δ = 169.1, 160.8, 154.3, 152.6, 149.9, 146.9, 135.4, 132.7, 129.5, 129.1, 128.2, 128.1, 127.2, 114.7, 111.2, 66.1, 35.9, 33.6, 11.3, 8.3, 7.8. HRMS (ESI+) (*m/z*): $[M-Li]^+$ calcd. for $C_{21}H_{19}N_2O_3S^+$, 379.1111; found, 379.1141.

(3*R*)-8-Cyclopropyl-7-(4-fluoro-naphthalen-1-ylmethyl)-5-oxo-2,3-dihydro-5*H*-thiazolo[3,2-*a*]pyridine-3-carboxylic acid (4). Following the general procedure for hydrolysis with **9** (250 mg, 0.61 mmol) gave **4** (202 mg, 84%) as a white solid. $[\alpha]_D$ = -5.8 (*c*

= 0.5, $CHCl_3$:MeOH (9:1)). IR (υ cm^{-1}) (neat) 1726, 1618, 1487, 1423, 1392, 1201, 1171. ^{1}H NMR (DMSO-d_6, 400 MHz) δ = 8.13-8.10 (m, 1H), 7.95-7.92 (m, 1H), 7.67-7.64 (m, 2H), 7.37-7.31 (m, 2H), 5.25 (d, 1H, *J* = 8.8 Hz), 5.23 (s, 1H), 4.46 (d, 1H, *J* = 17.6 Hz), 4.37 (d, 1H, *J* = 17.6 Hz), 3.72 (dd, 1H, *J* = 9.2, 11.6 Hz), 3.51 (d, 1H, *J* = 11.6 Hz), 1.74 -1.67 (m, 1H), 0.93-0.86 (m, 2H), 0.77-0.71 (m, 1H), 0.66-0.60 (m, 1H). ^{13}C NMR (DMSO-d_6, 100 MHz) δ = 169.7, 159.9, 157.1 (d, J_{CF} = 248 Hz), 155.6, 148.3, 132.7 (d, J_{CF} = 5 Hz), 130.9 (d, J_{CF} = 4 Hz), 127.5, 127.3, 126.5, 124.5, 123.1 (d, J_{CF} = 16 Hz), 120.4 (d, J_{CF} = 5 Hz), 113.3, 111.5, 109.2 (d, J_{CF} = 20 Hz), 63.3, 34.8, 31.7, 10.7, 7.4, 7.1. HRMS (ESI+) *m/z*: $[M+Na]^+$ calcd. for $C_{22}H_{18}FNO_3S$, 418.0889; found, 418.0870.

(3*R*)-8-cyclopropyl-7-(naphthalen-2-ylmethyl)-5-oxo-2,3-dihydro-5*H*-[1,3]thiazolo[3,2-*a*]pyridine-3-carboxylic acid (5). Following the general procedure for hydrolysis with **10** (60 mg, 0.15 mmol) gave **5** (53 mg, 92%) as a white solid. $[\alpha]_D$ **=** -17.5 (*c* = 0.2, DMSO). IR (υ cm^{-1}) (neat) = 1702, 1616, 1486, 1174. ^{1}H NMR (DMSO, 400 MHz) δ = 8.13-8.10 (m, 1H), 7.95-7.92 (m, 1H), 7.67-7.64 (m, 2H), 7.54 (s, 1H), 7.37-7.31 (m, 2H), 5.25 (d, 1H, *J* = 8.8 Hz), 5.23 (s, 1H), 4.46 (d, 1H, *J* = 17.6 Hz), 4.37 (d, 1H, *J* = 17.6 Hz), 3.72 (dd, 1H, *J* = 9.2, 11.6 Hz), 3.51 (d, 1H, *J* = 11.6 Hz), 1.74 -1.67 (m, 1H), 0.93-0.86 (m, 2H), 0.66-0.60 (m, 2H); ^{13}C NMR (DMSO, 100 MHz) δ = 169.7, 160.1, 157.0, 156.6, 148.5, 133.6, 133.1, 129.1, 128.7, 127.3, 126.8, 118.8, 114.3, 111.8, 107.8, 62.5, 55.4, 38.3, 31.6, 10.9, 7.9, 7.8. HRMS (ESI+) (*m/z*): $[M+H]^+$ calcd. for $C_{22}H_{20}NO_3S^+$, 378.1158; found, 378.1169.

Molecular Biology and Biochemistry

Site directed mutagenesis for PrfA G145S substitution and allelic replacement

10 ng of plasmid pLis35 (Sheehan et al., 1996) was amplified by PCR with a Phusion DNA polymerase (Thermo Scientific) using primers PrfA-G145S-F and PrfA-G145-R (Eiting et al., 2005) and digested overnight with 10U DpnI (Thermo Scientific) according to manufacturer's instructions. *E. coli* strain DH5α was transformed with a resulting reaction mix, transformants selected for plasmid DNA purification. Correct sequence of *prfA* gene carrying the G145S substitution was verified by sequencing the insert in the resulting plasmid pKVA609. The *prfA* G145S allele DNA fragment was excised from pKVA609 with PstI endonuclease, blunted using Mung Bean Nuclease (New England Biolabs) and ligated into SmaI site of the pMAD vector to construct the allelic replacement plasmid pKVA973. The *prfA* allele replacement with the *prfA** was performed using pKVA973 plasmid in *L. monocytogenes* strains EGDe and Δ*lmo0866* as described previously (Arnaud et al., 2004).

Protein Purification

pET28a (*prfA*)(Sheehan et al., 1996) was introduced into *E. coli* BL21(DE3) by heat-shocking. Transformants were grown in LB supplemented with kanamycin and chloramphenicol. At an OD_{600} of 0.8 the PrfA expression was induced by isopropyl β-D-1-thiogalactopyranoside (IPTG) for 3 h, and the cells were subsequently lysed by sonication. PrfA was extracted by Ni-NTA Superflow FF (Qiagen), followed by thrombin (GE-Healthcare) cleavage and dialyzed against buffer (200 mM NaCl, 10 mM Tris pH 7.5, 1 mM dithiothreitol (DTT)) at 4°C overnight. The thrombin cleaved protein had an N-terminal tail of residues GSHMASMTGGQQMGRGS followed by residues Glu7-N237. The protein was purified by MonoS 5/5 ion-exchange (GE-Healthcare). The column was eluted with a lineal gradient of 200-1000 mM NaCl, and the pure protein eluted at ~250 mM NaCl, 10 mM Tris pH 7.5, 1 mM DTT. The peak fractions of $PrfA_{WT}$ were pooled and concentrated using a Centriprep-10 centrifugal concentrator (Millipore) to a final concentration of 3.5 mg ml^{-1}.

Western blot

Overnight cultures of *L. monocytogenes* were diluted to an OD_{600} of 0.025 in BHI supplemented with DMSO, or the ring-fused 2-pyridone compounds dissolved in DMSO, resulting in a final concentration of 0.1% DMSO. The cultures were grown at 37 °C to an OD_{600} of 1.0. The supernatants and bacterial pellets were collected for protein precipitation and treated as follows:

LLO and P60 Western blotting detection: 1 ml of the culture supernatant was filtered through 0.22 µm PVDF filter (Millipore) and subjected to trichloroacetic acid (TCA) precipitation. 1/100 vol 2% sodium deoxycholate (Sigma Aldrich) was added to the samples and incubated at RT for 10 min. One fourth volume of ice cold 50% TCA was added to the samples and incubated on ice for 1 h. The samples were spun down (10 min, 18,000 rcf) and the precipitate washed in 80% ice cold acetone. The dried protein pellets were suspended in 1 × sample buffer (Laemmli, 1970) and subjected to SDS-PAGE and Western blotting using rabbit anti-LLO (#ab43018, Abcam) and HRP conjugated goat-anti rabbit secondary antibodies (#as09602, Agrisera) or anti-P60 (BioSite P6017) and HRP conjugated rabbit- anti mouse secondary antibodies (Dako P0260).

ActA, PrfA and P60 Western blotting detection: The cultures were added an equal volume of 1:1 EtOH:Ac and frozen at -20°C o/n. Subsequently the samples were centrifuged and the bacterial pellet lysed in lysisbuffer (20mM Tris pH 8.0, 50mM EDTA pH 8.0, 20% sucrose) added lysozyme and DNase. The samples were heated at 37°C for 1 hour and subjected to SDS-PAGE and Western blotting using anti-ActA and anti-PrfA R79IS4b (kindly provided by Pascale Cossart, Institute Pasteur, Paris, France) and HRP conjugated secondary antibodies (#as09602, Agrisera) or anti-p60 (BioSite P6017) and HRP conjugated rabbit- anti mouse secondary antibodies (Dako P0260).

ActA western blotting detection for intracellular bacteria: The collected bacterial pellets were resuspended in 100µl 1x sample buffer (Laemmli, 1970) and lysed by incubation at 98 °C for 20 min. 10µL of each sample was subjected to SDS-PAG and Western blotting using anti-ActA and HRP conjugated secondary antibodies (#as09602, Agrisera).

Antibody production

Production of antibodies against ActA was conducted by Agrisera. The antibodies were raised in rabbits, against a peptide mix consisting of Y21T(Kocks et al., 1993) and (NH_2-) CKDAGKWVRDKIDENPE (-$CONH_2$). The rabbits were immunized four times over a four month period, the first with FCA and the subsequent with FIA. The antisera was recovered 2 weeks after the last immunization.

Infection for determination of intracellular ActA levels

Caco-2 cells were seeded into 24 well plates (Corning #356408) and allowed to grow until confluent. Indicated bacterial strains were grown to OD_{600} = 1, added to CaCo-2 cells at a MOI of 10, centrifuged for 10 min at 800 g and allowed to infect for 30 minutes. After washing, 1ml DMEM supplemented with 100µM of **2** and 50µg/ml of gentamicin was added. For extraction of intracellular bacteria, the cells were first washed in PBS, and then lysed using PBS supplemented with 1% Triton X-100. The cell lysate was transferred to Eppendorf tubes, and centrifuged at 20238 g for 1 min. The supernatant was removed and the pellets stored at -20 °C before western blot analysis.

Hemolytic activity assay

Goat erythrocytes (Agrisera) were diluted to 10% in PBS pH 7.4. Overnight cultures of *L. monocytogenes* were diluted to an OD_{600} of 0.005 in BHI adjusted to pH 5.5 with HCl, supplemented with 0.3 M NaCl and DMSO or compounds dissolved in DMSO, resulting in a final concentration of 0.1% DMSO. The cultures were subsequently grown at 37 °C to an OD_{600} of 1.0. The supernatants were filtered through 0.22 µm PVDF filters (Millipore) and aliquoted in 96 well plates in triplicate. 10% erythrocyte solution was subsequently added to a final concentration of 5%. The samples were stored at 37 °C for 3 h, spun down and 100 µl of the supernatant measured at an optical density at 541 nm using a TECAN Infinite M200 plate reader. Samples were done with four replicates.

Northern blot

Northern blotting was performed as described previously (Tiensuu et al., 2013). In brief, 20 µg of RNA was separated on an agarose gel (1.2% agarose, 1 × HEPES buffer (20 mM HEPES, 5 mM NaAc, 1 mM EDTA, adjusted to pH 7), 7.3% formaldehyde). The gel was run in 1× HEPES buffer at 100 V for 4 h and the RNA was transferred to a Hybond–N membrane (Amersham/GE-lifesciences) by capillary transfer in 20 × SSC buffer. The membranes were cross-linked by UV-light, pre-hybridized at 60 °C in Rapid hyb buffer (Amersham/GE-lifesciences) for about 2 h and then hybridized with DNA probes at 60 °C overnight. Membranes were washed (0.5% SDS, 2 × SSC, room temperature for 15 min followed by 0.5% SDS, 0.1 × SSC 60 °C for 15 min), exposed in a phosphorimager cassette and developed using the Typhoon FLA9500 Scanner (HE Lifesciences). The probes were created by amplifying genomic *L. monocytogenes* EGDe DNA by PCR using primers BM1/BM2 for *hly*; actA-qF/actA-qR for *actA*; inlA-U/inlD for *inlA* and *inlAB*; plcA-U/plcA-D for *plcA* and *plcA-prfA* and tmRNA-U/tmRNA-D for

tmRNA. Sequences of primers are found below. Probes were subsequently labeled with α-32P dATP (PerkinElmer) using Megaprime DNA labelling system (Amersham/GE-lifesciences), according to the manufacturer's instructions.

Surface plasmon resonance (SPR)

SPR experiments was conducted in a Biacore X100 (Biacore/GE Healthcare), and performed mainly as described previously (Deshayes et al., 2012). In brief, biotinylated oligonucleotides (Promo40plcA-P14/Promo40plcAREV), containing the PrfA box of the *plcA* promoter, were immobilized on 1 flow-cell of a SA Chip (Biacore/GE-Healthcare). Assays were performed at 37 °C. Protein samples were diluted in HBS-EP+ (Biacore/GE-Healthcare) to a final concentration of 200 nM, and compounds at different concentrations were added, resulting in a final DMSO concentration of 0.5%. The samples were injected with a flow rate of 10 μL/min. HBS-EP+ was used as running buffer. Measurements were done with a contact time of 200 s, and a dissociation time of 120 s. This was followed by regeneration solution (0.1% SDS, 3 mM EDTA) with a contact time of 60 s. The flow-cell without any immobilized DNA was used for reference subtraction. Each run was repeated three times.

Isothermal titration calorimetry (ITC)

ITC experiments were performed using a MicroCal AutoiTC200. In a typical run, 25 automated injections of 1.65 µl with 170 s breaks in between injections were made at 25 °C with 600 rpm stirring speed on low feedback mode. The protein concentrations in the cell were varied between 25 and 100 µM while the compound concentration in the syringe was varied from 300 to 800 µM. The buffer for both protein and compound solutions was the same as in the final

step of the purification. The compounds were dissolved in DMSO, and then diluted with buffer. DMSO content in the final compound solutions did not exceed 0.5% (*v/v*). Data integration, fitting and evaluation were performed using the software Origin™ 7 with the ITC200 plugin provided by MicroCal/GE Healthcare.

Crystallography

Crystallization

Using 5' NcoI and 3' Acc651 restriction sites, the PrfA gene was cloned into a modified pET24d plasmid containing an upstream, in frame coding region for a 6-His tag and a Tobacco etch virus (TEV) protease cleavage site. The construct was transformed into the expression strain *E. coli* BL21 (DE3) pLysS (Novagen). The PrfA protein was expressed in *E. coli* as described above (Protein purification). Following cell lysis, the protein was extracted from the soluble fraction by Ni-NTA Superflow FF (Qiagen), followed by TEV protease cleavage and was dialyzed at 4°C overnight against buffer (200 mM NaCl, 20 mM NaP (NaH_2PO_4+Na_2HPO_4) buffer pH 7.1, and 1 mM dithiothreitol (DTT)). The TEV cleaved protein contains two non-native N-terminal residues GA followed by the native residues M1-N237. The protein was purified by Ni-NTA Superflow FF (Qiagen) followed by MonoS 10/10 ion-exchange (GE-Healthcare) and Superdex 75 16/60 size exclusion chromatography (GE-Healthcare). The MonoS column was eluted with a linear salt gradient between 100-1000 mM NaCl. The pure protein eluted at about 200 mM NaCl, 20 mM NaP, pH 6.5. Size exclusion chromatography was performed with the same buffer (200 mM NaCl and 20 mM NaP buffer pH 6.5). The peak fractions of $PrfA_{WT}$ were pooled and concentrated using a Centriprep-10 centrifugal concentrator (Millipore) to a final concentration of 3.5 mg ml^{-1}.

PrfA was co-crystallized with complex **1** (5 mol excess) using the hanging-drop vapor-diffusion technique at 18 °C. Crystals (0.1 × 0.4 × 0.04 mm^3) grew in 5 days after 1 μl of the protein solution (3.5 mg ml^{-1} PrfA, 200 mM NaCl, 20 mM NaP buffer, pH 6.5) was mixed with an equal volume of precipitant solution containing 20% PEG-4000, 16% isopropanol, 100 mM Na citrate pH 5.5 and allowed to equilibrate over a 1 ml solution of the precipitant in a Linbro plate (Hampton Research). Before data collection, the crystals were transferred to a cryo-protectant solution including 16 % v/v glycerol in the precipitant solution. The crystals were flash-cooled to 100 K in a cryo-stream (Oxford CryoSystems) and stored in liquid nitrogen. Diffraction data at 100 K were collected at the ESRF beamline ID29 (λ = 0.914 Å). Data collection statistics are shown in Table 2.

Phasing and refinement

Crystals of the *L. monocytogenes* PrfA-**1** complex belong to space group $P2_1$ and contain two protein chains, i.e. one biological dimer, in the asymmetric unit (dimer AB). The data set, collected from a single crystal, was processed with XDS (Kabsch, 1993) and scaled using AIMLESS from the CCP4 software suite (Bailey, 1994). The X-ray model of *L. monocytogenes* PrfA (PDB entry 2beo (Eiting et al., 2005) and X-ray intensity data from 57.0 to 2.25 Å resolution were used in molecular replacement searches with the program PHASER (Bailey, 1994; Mccoy et al., 2007) to recover the phase information. Positions of **1**, not present in the search model, were clearly visible in electron density maps and could be placed by LigandFit at site AI and BII. CC for ligand placements were 0.83 and 0.76, respectively. There is positive electron density at site BI, i.e. the symmetry-related site to AI but in monomer B, however, compound **1** could not be modelled at this site. Map inspection and manual model building was performed with COOT (Emsley and Cowtan, 2004; Emsley et al., 2010). Refinement was performed at 2.25 Å resolution with PHENIX (Adams et al., 2010; Terwilliger, 2004). Five

percent of the observed structure factors were not included in the refinement and were instead used for validation by free *R*-factor calculations. The refined structure comprises residues 2–237 of both monomers, two **1** molecules, and 22 water molecules that are well-defined in the electron density. 98% of all residues were in Ramachandran favored region, and 0% were Ramachandran outliers. Not modeled in the structure are the flexible residues 176-182 (similar to the 2beo structure). Weak electron density is also observed for one loop regions including residues 201-206 in monomer A (B-values > 200 $Å^2$). Overall the C-terminal domain is flexible with high B-values (>100 $Å^2$) for residues in the vicinity of the HTH motif (residues 155-175 and 183-214). Based on all main-chain atoms, the root-mean-square (RMS) deviations are ~0.9-1.0 Å comparing individually monomers A-B in PrfA with the reference model (PDB code 2beo, monomer A). The R_{work} and R_{free} of the final model are 20.3% and 25.4%, respectively. Refinement statistics are summarized in Table 2. Fig. 6a-c and Supplemental Figures S4 – S6 were prepared with CCP4mg (McNicholas et al., 2011). Fig. 6d-e was prepared with MOE (Chemical Computing Group Inc., 2011).

Oligonucleotides used in this work:

Oligonucleotide	Sequence
hly-U	GAAGCAAAGGATGCATCTGC
hly-D	CCATCTTTGTAACCTTTTCTTGG
actA-qF	AGCCCGGTTCCTTCGTTAAG
actA-gR	GGATTACTGGTAGGCTCGGC
inlA-U	GCAATATTAGTATTTGGCAGCG
inlA-D	CTAGATCTGTTTGTGAGACCG
plcA-U	TTTTTTACTTTCCCA

plcA-D	TGTTTTGCTCGTCTT
tmRNA-U	CGGCACTTAATATCTACGAGC
tmRNA-D	CCTCGTTATCAACGTCAAAGCC
PrfA-G145S-F	GGAAGCTTGGCTCTATTTGCTCTCAACTTTTAATCCTGACC
PrfA-G145S-D	GTCAGGATTAAAAGTTGAGAGCAAATAGAGCCAAGCTTCC

Supplemental References

Adams, P.D., Afonine, P.V., Bunkoczi, G., Chen, V.B., Davis, I.W., Echols, N., Headd, J.J., Hung, L.W., Kapral, G.J., Grosse-Kunstleve, R.W., *et al.* (2010). PHENIX: a comprehensive Python-based system for macromolecular structure solution. Acta Crystallogr D *66*, 213-221.

Arnaud, M., Chastanet, A., and Debarbouille, M. (2004). New vector for efficient allelic replacement in naturally nontransformable, low-GC-content, gram-positive bacteria. Applied and environmental microbiology *70*, 6887-6891.

Bailey, S. (1994). The Ccp4 Suite - Programs for Protein Crystallography. Acta Crystallogr D *50*, 760-763.

Chemical Computing Group Inc., S.S.W., Suite #910, Montreal, QC, Canada, H3A 2R7 (2011). Molecular Operating Environment. MOE.

Chorell, E., Pinkner, J.S., Phan, G., Edvinsson, S., Buelens, F., Remaut, H., Waksman, G., Hultgren, S.J., and Almqvist, F. (2010). Design and Synthesis of C-2 Substituted Thiazolo and Dihydrothiazolo Ring-Fused 2-Pyridones: Pilicides with Increased Antivirulence Activity. Journal of Medicinal Chemistry *53*, 5690-5695.

Deshayes, C., Bielecka, M.K., Cain, R.J., Scortti, M., de las Heras, A., Pietras, Z., Luisi, B.F., Nunez Miguel, R., and Vazquez-Boland, J.A. (2012). Allosteric mutants show that PrfA activation is dispensable for vacuole escape but required for efficient spread and Listeria survival in vivo. Mol Microbiol *85*, 461-477.

Eiting, M., Hageluken, G., Schubert, W.D., and Heinz, D.W. (2005). The mutation G145S in PrfA, a key virulence regulator of Listeria monocytogenes, increases DNA-binding affinity by stabilizing the HTH motif. Molecular microbiology *56*, 433-446.

Emsley, P., and Cowtan, K. (2004). Coot: model-building tools for molecular graphics. Acta Crystallogr D *60*, 2126-2132.

Emsley, P., Lohkamp, B., Scott, W.G., and Cowtan, K. (2010). Features and development of Coot. Acta Crystallogr D *66*, 486-501.

Kabsch, W. (1993). Automatic Processing of Rotation Diffraction Data from Crystals of Initially Unknown Symmetry and Cell Constants. J Appl Crystallogr *26*, 795-800.

Kocks, C., Hellio, R., Gounon, P., Ohayon, H., and Cossart, P. (1993). Polarized distribution of Listeria monocytogenes surface protein ActA at the site of directional actin assembly. J Cell Sci *105 (Pt 3)*, 699-710.

Laemmli, U.K. (1970). Cleavage of structural proteins during the assembly of the head of bacteriophage T4. Nature *227*, 680-685.

Mccoy, A.J., Grosse-Kunstleve, R.W., Adams, P.D., Winn, M.D., Storoni, L.C., and Read, R.J. (2007). Phaser crystallographic software. J Appl Crystallogr *40*, 658-674.

McNicholas, S., Potterton, E., Wilson, K.S., and Noble, M.E.M. (2011). Presenting your structures: the CCP4mg molecular-graphics software. Acta Crystallogr D *67*, 386-394.

Passner, J.M., Schultz, S.C., and Steitz, T.A. (2000). Modeling the cAMP-induced Allosteric Transition Using the Crystal Structure of CAP-cAMP at 2.1 Å Resolution. Journal of Molecular Biology *304*, 847-859.

Sheehan, B., Klarsfeld, A., Ebright, R., and Cossart, P. (1996). A single substitution in the putative helix-turn-helix motif of the pleiotropic activator PrfA attenuates Listeria monocytogenes virulence. Molecular microbiology *20*, 785-797.

Terwilliger, T. (2004). SOLVE and RESOLVE: automated structure solution, density modification, and model building. J Synchrotron Radiat *11*, 49-52.

Tiensuu, T., Andersson, C., Ryden, P., and Johansson, J. (2013). Cycles of light and dark co-ordinate reversible colony differentiation in *Listeria monocytogenes*. Molecular microbiology *87*, 909-924.
