# Supplementary material for: Attenuating Listeria monocytogenes Virulence by Targeting the Regulatory Protein PrfA
Source: Cell Chem Biol. 2016 Mar 17;23(3):404–14. doi: 10.1016/j.chembiol.2016.02.013 (PMC4802734; doi:10.1016/j.chembiol.2016.02.013)
Supplement: Document S2. Article plus Supplemental Information [file mmc2.pdf]

Article

Cell Chemical Biology

Attenuating *Listeria monocytogenes* Virulence by Targeting the Regulatory Protein PrfA

Graphical Abstract

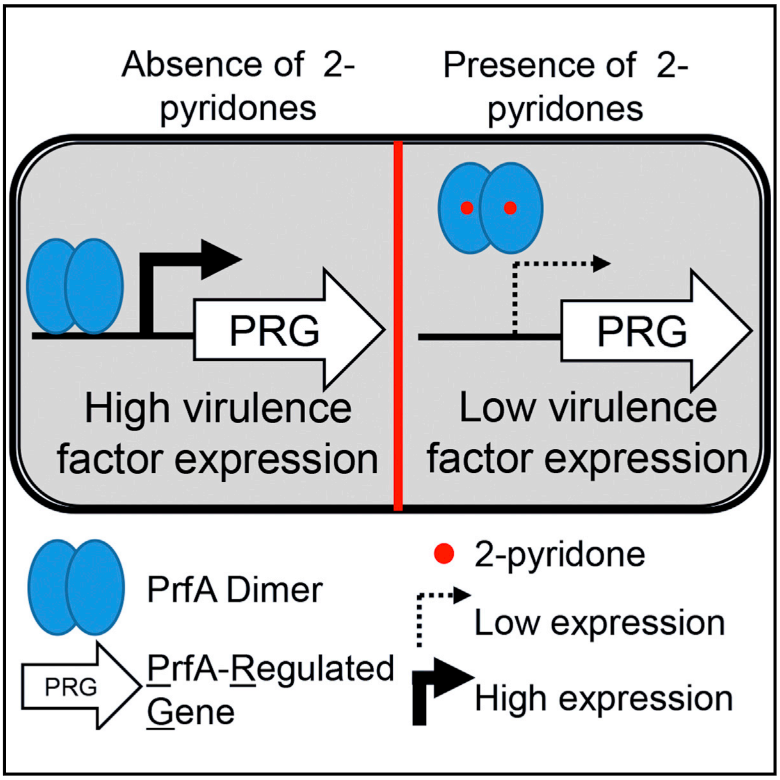

Authors

James A.D. Good,
Christopher Andersson,
Sabine Hansen, ...,
A. Elisabeth Sauer-Eriksson,
Fredrik Almqvist, Jörgen Johansson

Correspondence

elisabeth.sauer-eriksson@umu.se
(A.E.S.-E.),
fredrik.almqvist@umu.se (F.A.),
jorgen.johansson@umu.se (J.J.)

In Brief

In this study, Good et al. identify ring-fused 2-pyridones which attenuate *Listeria monocytogenes* infectivity and reduce the expression of key virulence genes by binding to the central virulence regulator PrfA.

Highlights

- Inhibitors of *L. monocytogenes* infectivity reduce virulence gene expression
- Binding of inhibitor to the PrfA regulator reduces affinity for its DNA motif
- First crystal structure of a Crp family regulator with an inhibitor
- Provides rationale for screening with Crp family transcriptional regulators

Good et al., 2016, Cell Chemical Biology *23*, 404–414
March 17, 2016 ©2016 The Authors
http://dx.doi.org/10.1016/j.chembiol.2016.02.013

Please cite this article as: Good et al., Attenuating *Listeria monocytogenes* Virulence by Targeting the Regulatory Protein PrfA, Cell Chemical Biology (2016), http://dx.doi.org/10.1016/j.chembiol.2016.02.013

Attenuating *Listeria monocytogenes* Virulence by Targeting the Regulatory Protein PrfA

James A.D. Good,[1,2,5] Christopher Andersson,[2,3,4,5] Sabine Hansen,[2,3,4,5] Jessica Wall,[2,3,4,5] K. Syam Krishnan,[1,2,5,6] Afshan Begum,[1,2] Christin Grundström,[1,2] Moritz S. Niemiec,[1] Karolis Vaitkevicius,[2,3,4] Erik Chorell,[1,2] Pernilla Wittung-Stafshede,[1,7] Uwe H. Sauer,[1,2] A. Elisabeth Sauer-Eriksson,[1,2,*] Fredrik Almqvist,[1,2,*] and Jörgen Johansson[2,3,4,*]

[1]Department of Chemistry
[2]Umeå Centre for Microbial Research (UCMR)
[3]Department of Molecular Biology
[4]Molecular Infection Medicine, Sweden (MIMS)
Umeå University, 901 87 Umeå, Sweden
[5]Co-first author
[6]Present address: Department of Chemistry, Mannam Memorial NSS College, Kottiyam, Kollam, Kerala 691571, India
[7]Present address: Department of Biology and Bioengineering, Chalmers University of Technology, 41296 Gothenburg, Sweden
*Correspondence: elisabeth.sauer-eriksson@umu.se (A.E.S.-E.), fredrik.almqvist@umu.se (F.A.), jorgen.johansson@umu.se (J.J.)

http://dx.doi.org/10.1016/j.chembiol.2016.02.013

This is an open access article under the CC BY license (http://creativecommons.org/licenses/by/4.0/).

SUMMARY

The transcriptional activator PrfA, a member of the Crp/Fnr family, controls the expression of some key virulence factors necessary for infection by the human bacterial pathogen *Listeria monocytogenes*. Phenotypic screening identified ring-fused 2-pyridone molecules that at low micromolar concentrations attenuate *L. monocytogenes* cellular uptake by reducing the expression of virulence genes. These inhibitors bind the transcriptional regulator PrfA and decrease its affinity for the consensus DNA-binding site. Structural characterization of this interaction revealed that one of the ring-fused 2-pyridones, compound 1, binds at two separate sites on the protein: one within a hydrophobic pocket or tunnel, located between the C- and N-terminal domains of PrfA, and the second in the vicinity of the DNA-binding helix-turn-helix motif. At both sites the compound interacts with residues important for PrfA activation and helix-turn-helix formation. Ring-fused 2-pyridones represent a new class of chemical probes for studying virulence in *L. monocytogenes*.

INTRODUCTION

In light of increasing antibiotic resistance, novel therapies are required to potentiate or succeed our current selection of therapeutic options (Davies and Davies, 2010). An alternative to classical antibiotics are drugs inhibiting the virulence of pathogenic bacteria. The first step in establishing this as a viable and effective therapeutic strategy is to understand how the virulence of pathogenic bacteria can be controlled (Allen et al., 2014; Clatworthy et al., 2007; Rasko and Sperandio, 2010). The Gram-positive bacterium *Listeria monocytogenes* is a saprophyte responsible for the severe disease listeriosis in humans upon ingestion (Freitag et al., 2009; Vázquez-Boland et al., 2001). Its ability to grow at low temperatures, in high-salt and low-oxygen conditions, makes *L. monocytogenes* one of the most problematic foodborne pathogens. Although the incidence rate is low, *L. monocytogenes* is capable of crossing key protective barriers within the body (e.g., intestinal, placental, and blood-brain) and causing severe diseases (e.g., bacteremia, meningitis, and meningoencephalitis) (Drevets and Bronze, 2008). Pregnant, immunocompromised, and other at-risk patients are vulnerable to invasive listeriosis, and the high mortality rates within these subpopulations (~20%–40%) are a stark demonstration of the clinical difficulty in dealing with these infections (Drevets and Bronze, 2008; Hamon et al., 2006; Jackson et al., 2010; Vázquez-Boland et al., 2001).

The virulence factors governing host invasion and infection by *L. monocytogenes* have been well elucidated (Vázquez-Boland et al., 2001). The bacterium adheres to and enters both phagocytic and non-phagocytic cells, by using specific adhesins (e.g., InlA and InlB), depending on the cell type. With the aid of listeriolysin O (LLO) and a phospholipase (PlcA), the bacterium lyses the phagosome and proceeds to replicate intracellularly (Schnupf and Portnoy, 2007). Once in the cytoplasm, the bacterium uses the ActA protein to recruit the Arp2/3 complex facilitating the formation of an actin comet-tail, by which *L. monocytogenes* is able to move to an adjacent cell without exposing itself to the extracellular environment. ActA has also been shown to play a role during bacterial attachment and uptake into epithelial cells (Garcia-Del Portillo and Pucciarelli, 2012; Suarez et al., 2001). Within the next cell, the bacterium degrades the double-membrane vacuole and perpetuates the infection cycle (Hamon et al., 2006; Portnoy et al., 2002). The expression of the majority of virulence genes required for these processes is regulated by PrfA, a transcriptional activator from the Crp/Fnr family of regulators (Scortti et al., 2007). Members of this family bind as homodimers to consensus DNA sequences found in the promoter region of regulated genes (Won et al., 2009). PrfA positively regulates the expression of the above

and other Listerial virulence factors (Freitag et al., 2009; Scortti et al., 2007), and a Δ*prfA* strain is avirulent (Andersson et al., 2015; Chakraborty et al., 1992; Freitag et al., 1993; Gripenland et al., 2014). Structurally, each monomer of PrfA comprises an N-terminal eight-stranded β-barrel domain connected by an α helix linker to a C-terminal α/β domain (Eiting et al., 2005; Vega et al., 2004). The C-terminal region contains the winged helix-turn-helix (HTH) motif responsible for binding to consensus promoter sequences (Eiting et al., 2005; Vega et al., 2004). While most Crp family members require a small molecule cofactor for DNA binding (e.g., cAMP for Crp in *Escherichia coli*) (Won et al., 2009), PrfA is capable of binding to its DNA consensus sequences with low affinity even in the absence of a cofactor (Eiting et al., 2005). Nonetheless, the activity of PrfA is known to increase under inducible conditions, and this has led to the hypothesis that the intercellular mechanism of activation may be regulated by a host-derived or host-regulated cofactor (Freitag et al., 2009; Scortti et al., 2007). Recently, it was suggested that PrfA activation follows a two-step process, where PrfA needs to be in a reduced form for DNA binding, followed by interaction of PrfA with reduced glutathione for transcriptional activity at targets genes (Reniere et al., 2015). Structural evidence toward an allosteric mode of activation for PrfA in vivo has been provided by the crystal structure of the constitutively active PrfA mutant $PrfA_{G145S}$ (Eiting et al., 2005; Ripio et al., 1997; Vega et al., 2004). This amino acid substitution repositions the HTH motif in an ordered and exposed "active" conformation with increased DNA-binding affinity, in contrast to $PrfA_{WT}$ where the HTH motif remains flexible and partially disordered (Eiting et al., 2005).

We previously developed ring-fused 2-pyridone scaffolds which contain a peptidomimetic backbone (Svensson et al., 2001). Originally designed to mimic interactions between subunits in the chaperone-usher pathway responsible for pilus assembly in uropathogenic bacteria, we subsequently developed inhibitors with discrete substitution patterns from this scaffold, which both interact with structures important for *E. coli* adhesion to eukaryotic cells (Cegelski et al., 2009; Emtenäs et al., 2002; Pinkner et al., 2006) and had broader impact on virulence regulation (Greene et al., 2014). We were therefore interested to investigate whether 2-pyridones could affect *L. monocytogenes* virulence-associated phenotypes. In this study, we have conducted a phenotypic screen and identified several ring-fused 2-pyridones that attenuate *L. monocytogenes* uptake into epithelial cells and decrease virulence gene expression. We describe how these inhibitors interact directly with the transcriptional regulator PrfA and weaken its DNA-binding capacity. Furthermore, we provide the first structural detail of a Crp family protein with a bound inhibitor by presenting the crystal structure of PrfA in complex with one 2-pyridone, and propose possible modes of action.

RESULTS

Ring-Fused 2-Pyridones Attenuate *L. monocytogenes* Uptake

Using flow cytometry, we performed an infection screen based on HeLa cells infected with GFP-carrying *L. monocytogenes*. The putative virulence-inhibiting ability of ring-fused 2-pyridones from our in-house collection was assessed, and several close analogs that significantly reduced the relative infection at 100 μM were identified (Figures S1A and S1B). At a concentration of 10 μM, two 1-naphthyl derivatives effectively reduced *L. monocytogenes* uptake by HeLa cells by 80%–90% (C10 [**1**] and KSK 67 [**2**]), whereas a related 3-quinoline analog, KSK 29 (**3**), was less effective (Figure S1B). Viable count experiments verified these flow cytometry results, with compounds **1** or **2** reducing the level of *L. monocytogenes* uptake relative to untreated controls at 10 and 1 μM (Figure 1A). Furthermore, when we performed a time-course experiment monitoring the infection dynamics more closely, we observed an inability of *L. monocytogenes* to replicate within Caco-2 cells after treatment with compound **2**, which displayed the largest efficiency in the uptake experiment (Figure 1B, left panel). Although the generation time of *L. monocytogenes* was slightly longer in presence of compounds **1** or **2** at 100 μM compared with the DMSO control (45, 50, and 43 min, respectively), it is unlikely that this would be sufficient to explain the dramatic decrease in infectivity observed with these compounds at 100 μM and at lower concentrations (Figures S1C and 1). The generation time in the presence of compound **3** was unaffected compared with the DMSO control (43 min, Figure S1C).

Ring-Fused 2-Pyridones Reduce Virulence Factor Expression in *L. monocytogenes*

To understand how the compounds attenuated the infectivity of *L. monocytogenes*, the expression of virulence genes was analyzed in bacteria grown with or without 100 μM of **1**, **2**, or **3**. We initially investigated the expression of the *hly* gene which encodes the hemolysin LLO, and found it was downregulated by **1** or **2** compared with the untreated control, but not affected by compound **3** (Figure S2). Together with most virulence genes in *L. monocytogenes, hly* is positively regulated by the transcriptional activator PrfA (Freitag et al., 2009; Scortti et al., 2007), therefore we examined whether the expression of other PrfA-regulated genes was affected by treatment with compounds **1** and **2**. A reduction in the levels of the virulence genes *hly, actA*, and *plcA* transcripts could be observed after treatment with compounds **1** and **2** (but not **3**), whereas the expression of *inlA* and *inlB* was unaffected (Figure S2), possibly because basal expression of the latter are also controlled by other regulatory factors, such as the stress sigma factor σ^B (Stritzker et al., 2005). We next investigated whether the virulence protein levels of the two major virulence factors, LLO and ActA, were affected by compound treatment. Of the 2-pyridones tested, compounds **1** and **2** displayed similar properties. At 100 μM of **1** or **2**, expression of LLO and ActA was abolished, without a concomitant effect on PrfA protein levels or on the expression of the non-PrfA-regulated virulence factor P60 (Figures 2A and 2B). The reduction in LLO and ActA expression following treatment with **1** and **2** was dose dependent, with decreased levels down to ≤3.3 μM (Figures 2A and 2B). In contrast, compound **3** did not reduce LLO or ActA expression even at the highest concentration tested of 100 μM, in agreement with the lack of efficacy of **3** in abrogating *hly* or *actA* gene expression (Figures 2C and S2). Across all concentrations of **1**, **2**, or **3**, PrfA expression remained unchanged, indicating that the virulence attenuation of **1** and **2** was not mediated through reduced PrfA protein levels (Figures 2A–2C). Once a host cell is invaded by

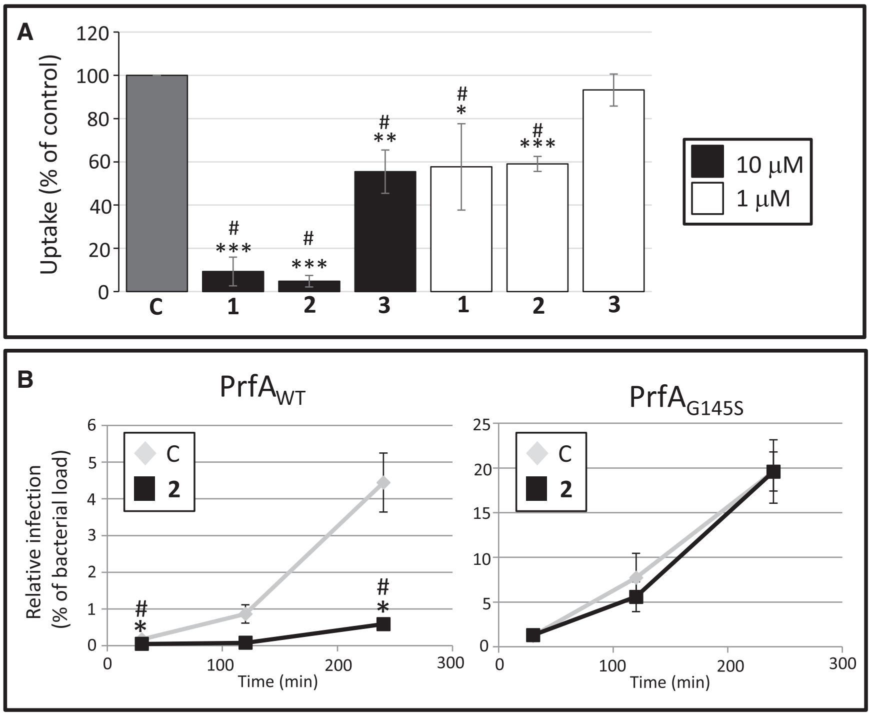

Figure 1. Ring-Fused 2-Pyridones Inhibit *L. monocytogenes* Uptake by Cultured Cells

(A) Relative uptake (%) compared with control as determined by viable count measurements. *L. monocytogenes* strain EGDe (WT) treated with compounds **1–3** at the indicated concentrations (10 μM, black bars; 1 μM, white bars) was allowed to infect HeLa cells for 1.5 hr. All samples were correlated to the DMSO control (C, gray bar), which was arbitrarily set at 100%. Error bars show SDs. Significance was tested using Student's t test (two-tailed, significant differences are shown by asterisks; *$p < 0.05$; **$p < 0.01$, and ***$p < 0.001$) and Dunnett's test (significant differences are shown by #). See also Figure S1.

(B) Time-course infection dynamics of *L. monocytogenes* harboring $PrfA_{WT}$ or $PrfA_{G145S}$ after treatment with compound **2**. Caco-2 cells were infected with *L. monocytogenes* strains carrying $PrfA_{WT}$ (left panels) or $PrfA_{G145S}$ (right panels) on the chromosome in the presence of DMSO (C) or compound **2** (50 μM). The amount of intracellular bacteria was measured using viable counts at indicated time points post infection and divided by the bacterial load used in the infection (prior to antibiotic treatment) arbitrarily set as % (n = 3). It should be noted that infection of Caco-2 cells with a strain harboring $PrfA_{G145S}$ is more effective than with a strain carrying $PrfA_{WT}$. Error bars show SDs. Significance was tested using Student's t test (two-tailed, significant differences are shown by asterisks; *$p < 0.05$) and Dunnett's test (significant differences to the control are shown by #).

L. monocytogenes, ActA expression is massively induced in a PrfA-dependent manner (Freitag et al., 2009; Scortti et al., 2007). We therefore analyzed whether ActA expression was affected at different time points when 100 μM of compound **2** was added 30 min post infection. The induction of intracellular ActA levels observed in the DMSO control 4 hr post infection, was weakened in compound-treated cells, although not completely abolished (Figure 2D). This indicates that the effectiveness of the compounds decreased post infection. We next examined whether ring-fused 2-pyridones could also reduce virulence factor expression in other *L. monocytogenes* strain backgrounds or serotypes. Addition of compound **1** effectively reduced LLO expression in *L. monocytogenes* serotypes associated with sporadic cases (10403S [serotype 1/2a] and LO28 [serotype 1/2c]) as well as a serotype associated with epidemic outbreaks (F2365 [serotype 4b]), whereas the levels of PrfA were unaffected in these strains (Figure 3A). In addition, compound **2** effectively inhibited uptake of the F2365 strain into Caco-2 cells (Figure 3B).

Virulence Regulation by the Mutant $PrfA_{G145S}$ Is Not Affected by Ring-Fused 2-Pyridones

The reduced levels of the virulence factors LLO and ActA, but not of PrfA (Figures 2A and 2B), suggested that **1** and **2** directly affected the activity of PrfA. Introducing a G145S amino acid substitution in PrfA generates a constitutively active mutant that contains a stabilized HTH DNA-binding motif, as opposed to the structurally undefined HTH present in $PrfA_{WT}$ (Eiting et al., 2005; Ripio et al., 1997). Examining the effect of the compounds on $PrfA_{G145S}$ in comparison with $PrfA_{WT}$ would indicate whether **1** and **2** directly inhibited the PrfA activation process, or if their effects were mediated after formation of the HTH DNA-binding motif. To test this, a *prfA*$_{G145S}$ allele was introduced at its native site on the chromosome, such that $PrfA_{G145S}$ would be produced, and this strain was treated with **1**, **2**, or **3** at 100 μM. In the $PrfA_{G145S}$-expressing strain, virulence gene expression as well as virulence protein levels remained essentially unaltered in the presence of the compounds (Figures 4 and S2). Once again, the levels of PrfA and P60 proteins did not vary considerably after treatment with the different compounds. We next challenged whether this mutation could also overcome the inhibitory effects of these compounds in a time-course cellular infection assay, with the most effective compound from the cellular uptake experiments, compound **2**. While *L. monocytogenes* containing $PrfA_{WT}$ was unable to replicate within Caco-2 cells in presence of **2** at 100 μM (Figure 1B, left panel), a strain expressing $PrfA_{G145S}$ overcame the inhibitory effects and established an infection comparable with the untreated control (Figure 1B, right panel). Collectively, these data strongly indicated that the 2-pyridones **1** and **2** reduced *L. monocytogenes* virulence by attenuating PrfA activity, and furthermore suggested that this process occurred prior to formation of the DNA-binding HTH motif, since the $PrfA_{G145S}$ protein carrying the stabilized HTH motif was not affected by the compounds.

Ring-Fused 2-Pyridones Directly Bind to PrfA In Vitro

We next determined whether **1** and **2** directly bound to PrfA via isothermal titration calorimetry (ITC). This in vitro method, with purified PrfA protein, allows measurement of the thermodynamic

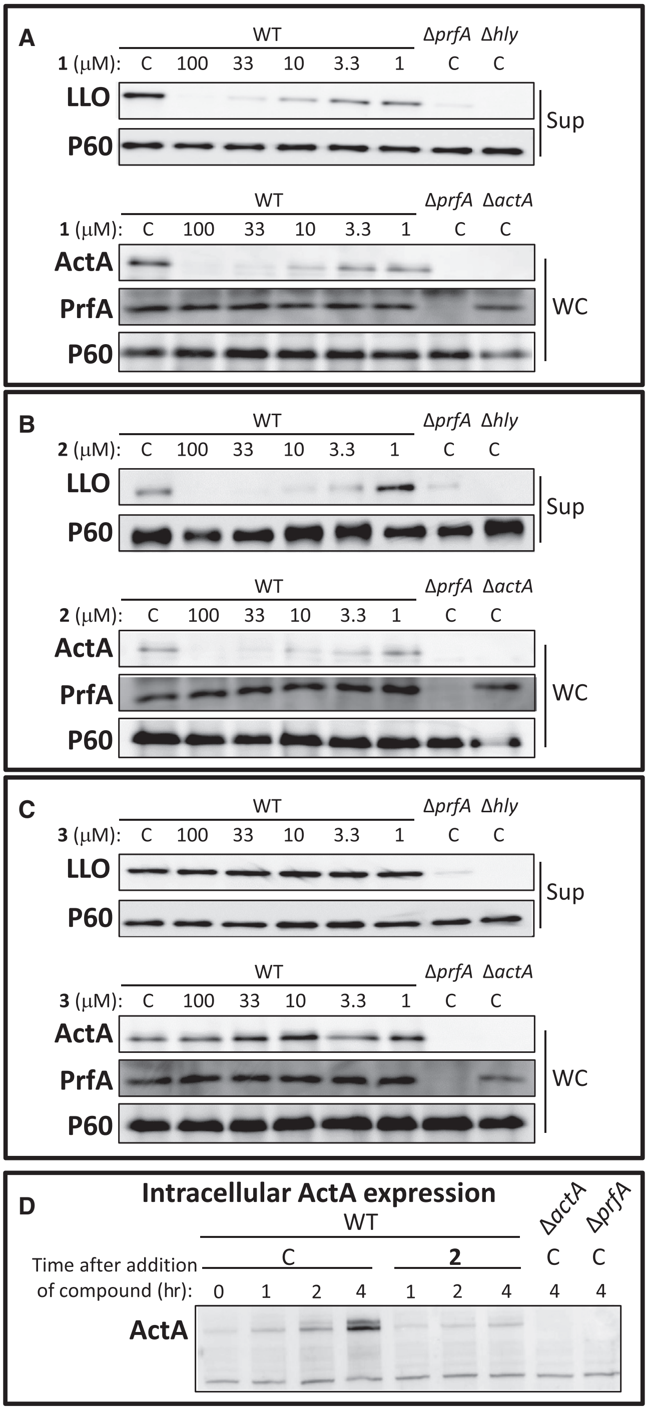

Figure 2. Compounds 1 and 2 Reduce Virulence Factor Expression in *L. monocytogenes*

(A–C) Protein extracts were isolated from indicated *L. monocytogenes* strain in the absence (C, equivalent volume of DMSO) or presence of **1** (A), **2** (B), or **3** (C) at the indicated concentration, and the specified proteins (ActA, LLO, PrfA, or P60 (control)) were detected by western blot using specific antibodies. Upper panels show secreted fractions (Sup) of indicated samples; lower panels show whole cells fractions (WC) of indicated samples. The images are a representative of three individual experiments. (D) Compound **2** abrogates intracellular induction of ActA expression. Caco-2 cells were infected with the indicated bacteria and treated 30 min post infection with 100 μM of **2** or the equivalent volume of DMSO (C). Total cellular protein was extracted at indicated time points after addition of compound and subjected to SDS-PAGE. The levels of ActA protein were visualized by western blot using a specific antibody. The images are a representative of three individual experiments.

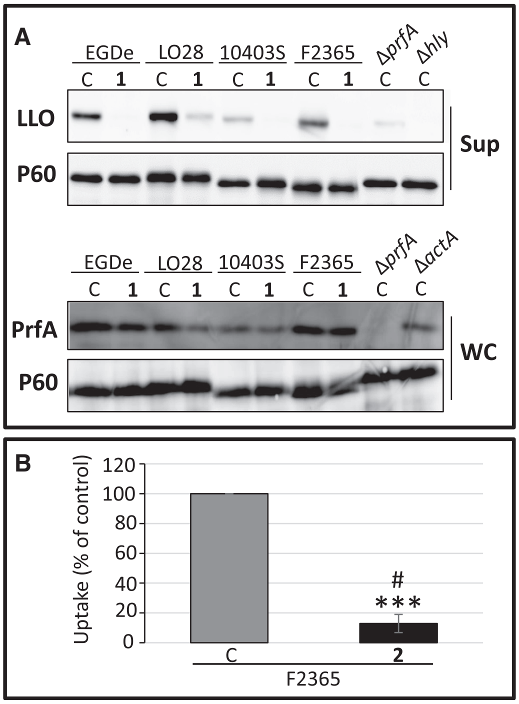

Figure 3. Compounds 1 and 2 Reduce Virulence Factor Expression and Uptake of Multiple Serotypes of *L. monocytogenes*

(A) Compound **1** reduces LLO expression in different *L. monocytogenes* strain backgrounds. Protein extracts were isolated from indicated *L. monocytogenes* strain in the absence (C, equivalent volume DMSO) or presence of 100 μM of **1** and the specified proteins (LLO PrfA, or P60 (control)) were detected by western blot using specific antibodies. Upper panels show secreted fractions (Sup) of indicated samples; lower panels show whole-cell fractions (WC) of indicated samples.

(B) Compound **2** inhibits the uptake of an *L. monocytogenes* strain of serovar 4b. *L. monocytogenes* strain F2365 was allowed to infect Caco-2 cells for 2 hr with **2** (50 μM). All samples were correlated to the DMSO-treated control (C, gray bar) which was arbitrarily set at 100%. Error bars show SDs. Significance was tested using Student's t test (two-tailed, significant differences are shown by asterisks; ***$p < 0.001$) and Dunnett's test (significant differences to the control were shown by #).

parameters of binding from the heat development that occurs upon ligand-protein interactions (Ladbury et al., 2010). The data presented in Figure 5A show that binding of **1** to $PrfA_{WT}$ occurs with negative enthalpy in a 1 to 1 stoichiometry per monomer of homodimeric PrfA. From analysis of the integrated heat peaks as a function of ligand-to-protein ratio using a 1 to 1 binding model, a dissociation constant (K_D) value for **1** binding to PrfA of ≈1 μM was determined (Table 1). Compound **2**, and unexpectedly **3**, also exhibited micromolar affinities for $PrfA_{WT}$ and interacted in 1 to 1 stoichiometry (Table 1 and Figure S3). We examined the binding of compounds **1–3** to the constitutively active PrfA mutant $PrfA_{G145S}$ and found that all three ligands bound to $PrfA_{G145S}$ with comparable affinity for **2** and reduced affinity for **1** and **3** (Table 1). These data suggested that the

Please cite this article as: Good et al., Attenuating *Listeria monocytogenes* Virulence by Targeting the Regulatory Protein PrfA, Cell Chemical Biology (2016), http://dx.doi.org/10.1016/j.chembiol.2016.02.013

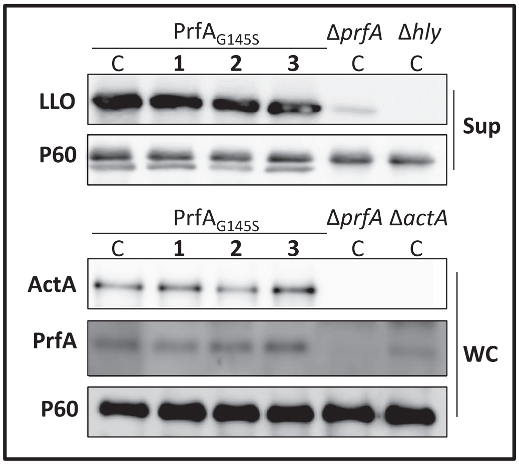

Figure 4. Compounds 1, 2, and 3 Cannot Inhibit LLO and ActA Expression in a Strain Expressing a Constitutively active PrfA Protein PrfA$_{G145S}$

The indicated strains were grown with **1–3** at 100 μM or DMSO (C). Upper panels show secreted fractions (Sup) of indicated samples; lower panels show whole-cell fractions (WC) of indicated samples. The levels of LLO, ActA, PrfA, and P60 (control) proteins are visualized by western blot using specific antibodies. The images are a representative of three individual experiments. See also Figure S2.

compounds do not bind at the DNA-binding HTH motif, since this region differs in conformation between the mutant (folded) versus the wild-type (WT) (partially disordered) (Eiting et al., 2005).

Compounds 1 and 2 Inhibit the DNA Binding of PrfA$_{WT}$, but Not PrfA$_{G145S}$

To further characterize the binding of **1–3** with PrfA in vitro, and analyze if such binding affects the DNA-binding properties of PrfA, we performed surface plasmon resonance (SPR) experiments. In these in vitro experiments, the ability of PrfA (WT or G145S), pre-incubated with **1**–**3**, to bind the *hly*-promoter DNA was analyzed. Compounds **1** and **2**, which reduced infectivity and the expression of key virulence factors in the cellular assays, both effectively reduced the binding of PrfA$_{WT}$ to *hly*-DNA at low micromolar levels via SPR (half maximal inhibitory concentration [IC$_{50}$] ≈ 6–7 μM; Figure 5B). The quinolone-containing compound **3**, which had only a slight effect in the cellular assays and did not attenuate virulence factor expression, could only inhibit the binding of PrfA$_{WT}$ to *hly*-DNA at higher concentrations (IC$_{50}$ ≈ 30 μM), indicating it was less effective at preventing the PrfA-*hly* interaction (Figure 5B). Furthermore, all the compounds had minimal impact upon the binding of PrfA$_{G145S}$ to *hly*-DNA at concentrations up to 100 μM (Figure 5C). Taken together, we conclude that while **1–3** bind to the constitutively active mutant PrfA$_{G145S}$, this has no significant impact on its ability to bind DNA.

Crystal Structure of PrfA$_{WT}$-1 Complex

To understand the structural basis for inhibition, we determined the crystal structure of the PrfA$_{WT}$-**1** complex at a resolution of 2.25 Å (Table 2). The PrfA$_{WT}$-**1** complex was co-crystallized in the space group P2$_1$ where the asymmetric unit contains one biological dimer: monomers A and B (Figure 6A). Binding of **1** to PrfA$_{WT}$ was confirmed at two sites, AI in monomer A and BII in monomer B, from difference Fourier electron density maps (Figures 6B, 6C, and S4).

The PrfA$_{WT}$-**1** dimer structure is similar to the previously determined structures of PrfA$_{WT}$ (Eiting et al., 2005) (Figures 6A and S5). Each monomer consists of an N-terminal domain (residues 1–108) and a C-terminal DNA-binding domain (residues 138–237) linked by a long α helix (αC, residues 109–137). Both the N- and C-terminal domains constitute an α/β fold. Hydrophobic interactions between symmetry-related αC helices and loops β6-β7 stabilize the dimer interface (Figure 6A). Two α helices in the C-terminal domain, αE (residues 170–178) and αF (residues 183–195) constitute the helices of the typical HTH motif present in many prokaryotic transcription factors. In PrfA$_{WT}$, parts of the first helix and the connecting turn of the PrfA HTH motif were not defined by electron density and have been assumed to be flexible (Eiting et al., 2005). This was also the case for the PrfA$_{WT}$-**1** complex structure determined here.

Compound **1** binds PrfA at two sites referred to as AI and BII. Site AI is found at the so-called interdomain tunnel in monomer A, situated between the N- and C-terminal domains (Figure 6B). The interdomain tunnel is positioned between the C-terminal helices αH and αI and the secondary structural motifs αC, αD, and β5 (Eiting et al., 2005). Compound **1** binds with high occupancy at site AI, but no ligand bound to the tunnel in monomer B (i.e., to the BI site). At binding site AI the carboxylate is in close proximity with the charged ε-amino groups of Lys64 and Lys122, indicating the potential for both electrostatic interactions and a network of hydrogen bonding, which in the case of Lys122 are mediated through the bulk solvent (Figure 6D). The naphthyl ring occupies a hydrophobic part of the tunnel bounded by the peptide backbones of Tyr62, Gln146, Ile149, and Leu150, and the cyclopropyl motif is surrounded between the peptide backbone of Gln123 and the phenyl rings of Tyr63, Phe67, and Tyr126, respectively. We reason that the peptidomimetic bicyclic 2-pyridone backbone provides the key recognition motif for binding to PrfA at the AI site, with the carboxylate forming electrostatic interactions with residues known to affect the activation of PrfA (Lys64 and Lys122) (Xayarath et al., 2011), and the naphthyl motif providing a lipophilic anchor into the interdomain tunnel. The second high-occupancy binding site for compound **1**, i.e., the BII site, is found at a hydrophobic pocket in the vicinity of the HTH motif in monomer B (Figure 6C). No ligand bound to the corresponding site in monomer A (i.e., to the AII site). The BII site is positioned in a pocket formed by the αC and αD helices and β5 strand from monomer B and the C-terminal end of the αC helix from monomer A (Figure 6C). At site BII, the carboxylate group of compound **1** forms hydrogen bonds to the main chain nitrogen atom of B-Tyr62, and to the hydroxyl group of B-Tyr126 and the 2-pyridone carbonyl hydrogen bonds with the side chain amino group B-Gln61. The naphthyl group of compound **1** at site BII is sandwiched between the side chains of four phenylalanines: Phe131 and Phe134 from both monomers in the dimer (Figure 6E). The charged lysine of B-130 is also sufficiently close (~4.8 Å) for electrostatic interactions with the carboxylate group to affect the free energy of binding.

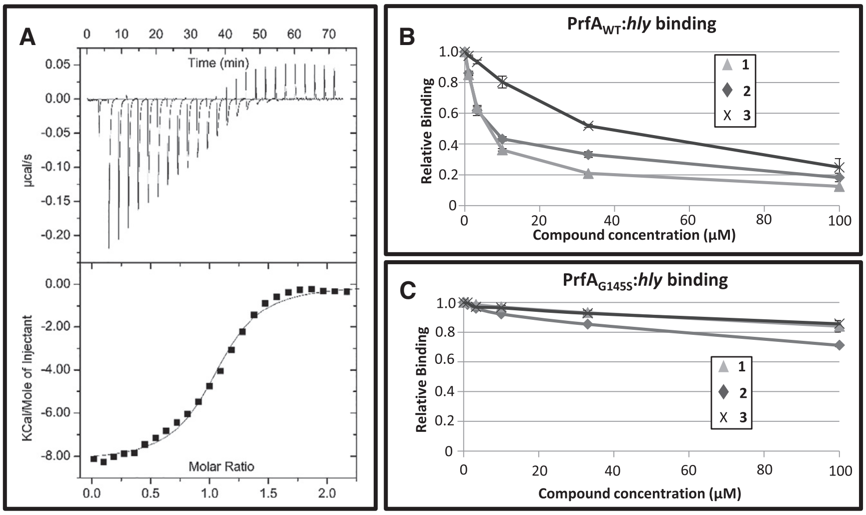

Figure 5. Ring-Fused 2-Pyridones Bind to PrfA and Decrease the Affinity of $PrfA_{WT}$, but Not $PrfA_{G145S}$, for *hly*-DNA

(A) ITC titration of **1** to $PrfA_{WT}$. Upper panel, heat pulses versus injections; lower panel, integrated heat signals versus molar ratio of **1** to $PrfA_{WT}$.

(B) and (C) SPR plots of relative binding affinity of $PrfA_{WT}$ and $PrfA_{G145S}$ with **1–3** to *hly*-DNA. $PrfA_{WT}$ (B) or $PrfA_{G145S}$ (C) was incubated with the indicated amounts of **1**, **2**, or **3**. The samples were analyzed by SPR and the relative binding capacity plotted. All compounds were compared with DMSO, which was set to 100% (n = 3). Data represents the mean values with SDs.

DISCUSSION

In this work, we identified ring-fused 2-pyridone inhibitors by phenotypic screening which effectively attenuated *L. monocytogenes* uptake into cultured cells at low micromolar concentrations. Compounds **1** and **2**, which were investigated in most detail, exhibited similar reductions in virulence and infectivity across all assays. A structurally discrete third analog, **3**, consistently failed to attenuate uptake although still binding PrfA in vitro, which may be attributable to lower intrinsic efficacy (Figure 5B) and less optimal physicochemical properties than **1** and **2**. Collectively, the results indicate that **1** and **2** act by binding to and preventing the activation of PrfA, the central transcriptional regulator of virulence in *L. monocytogenes*. The ring-fused 2-pyridone molecules bind to purified PrfA with K_D values in the low micromolar range (Table 1 and Figure 5). Consequently, this lowers the affinity of the protein for its DNA consensus sequences within the promoter regions of virulence genes (Figure 5B). This reduced DNA-binding affinity in turn abolishes the expression of key virulence factors, which reduces uptake in epithelial cells (Figures 1, 2, and 3; Figures S1 and S2).

Our functional and structural studies support a model where **1** and related inhibitors act either by preventing the structural rearrangements needed for activation of PrfA through stabilization of the HTH motif, or alternatively by preventing the concerted reorganization of the monomers that move the DNA-binding domains into positions compatible with DNA binding (Levy et al., 2008; Schultz et al., 1991). Previous structural studies on PrfA and the constitutively active $PrfA_{G145S}$ mutant revealed that rearrangements at the dimer interface enable the stabilization of the HTH motif in $PrfA_{G145S}$ into a structurally defined conformation with increased affinity to DNA (Eiting et al., 2005). Comparison between the two structures suggested that for DNA binding to occur, helix αC needs to straighten leading to rearrangements of β4-β5. Phe134, positioned on αC, flips over to fill the void created between helices αC and αD. Furthermore, Tyr62 positioned at β5 moves to fill the same void and to bring the side chains of Ile57 and Asn59 into direct contact with the now structurally defined first helix in the HTH motif (Eiting et al., 2005). Compound **1** binds to site AI, a hydrophobic interdomain tunnel previously suggested to constitute a plausible cofactor binding site (Eiting et al., 2005) and to site BII, a hydrophobic pocket in the vicinity of the HTH motif (Figures 6, S6A, and S6B). We reason that inhibitor binding at AI could prevent conformational changes of the PrfA protein, and thus block the inducible activation of PrfA. In support, the structural data demonstrate electrostatic interactions between residues Lys64 and Lys122 with the carboxylate moiety of inhibitor **1**, and van der Waal contacts between the aliphatic backbone of Tyr62 and the naphthyl ring of **1** (Figure 6D). Both Lys64 and Lys122 have previously been implicated as being involved in PrfA activity: K64Q or K122Q mutants exhibited reduced ActA expression, weakened capability to bind *hly*-DNA, and attenuated virulence (Xayarath et al., 2011). At site BII compound **1** binds in the vicinity of the flexible HTH motif (Figures S6B and S6C). At this site the naphthyl group of compound **1** sterically prevents restructuring of residues Phe131 and Phe134 into positions needed for formation of an HTH motif compatible with DNA binding (Figure S6D) (Levy et al., 2008; Schultz et al., 1991). One interesting observation from the in vitro experiments was the binding of **1–3** to both $PrfA_{WT}$ and $PrfA_{G145S}$ determined by ITC, despite the lack of efficacy against strains harboring the constitutively active G145S mutant (Table 1). Since the $PrfA_{G145S}$ mutant that has structurally defined HTH motifs can still bind to its consensus DNA when in complex with **1** (Figure 5C), one possibility is that compound **1** only binds at the AI site in the $PrfA_{G145S}$ mutant. Binding of **1** in the tunnel only of $PrfA_{G145S}$ needs, however, to be structurally verified in future, and the similar binding efficacies of **1–3** in vitro suggest that additional complexities may emerge in the in vivo mechanism of PrfA inhibition.

It has previously been shown that ActA is important for bacterial uptake into epithelial cells including Caco-2 and Hela (Suarez et al., 2001). ActA has also been suggested to promote the initial contact between the bacteria and the cell by binding Heparan sulfate proteoglycan receptor, thereby allowing a stable contact between InlA or InlB with E-cadherin and Met, respectively (Garcia-Del Portillo and Pucciarelli, 2012). Thus, our results showing a decreased uptake of bacteria in the presence of compounds **1** and **2** is consistent with a reduced expression of ActA (Figures 1 and 2). Furthermore, while compounds **1** and **2** were effective at attenuating virulence when added prior to infection, only high concentrations of **2** reduced ActA expression post infection

Table 1. Binding Parameters of 1–3 to $PrfA_{WT}$ and $PrfA_{G145S}$ as Determined by ITC

Protein	Compound	K_D (M)	ΔH_A (kcal/mol)	ΔS_A (cal/mol·K)
$PrfA_{WT}$	**1**	1.0 (±0.2) × 10^{-6}	−8.4 ± 0.1	−0.8 ± 0.1
	**2**	6.7 × 10^{-6}	−6.5	2.8
	**3**	1.6 (±0.2) × 10^{-6}	−8.4 ± 0.2	−1.5 ± 0.9
$PrfA_{G145S}$	**1**	7.75 × 10^{-6}	−6.2	2.6
	**2**	5.4 × 10^{-6}	−4.1	10.4
	**3**	5.0 (±1.8) × 10^{-6}	−6.2 ± 1.0	3.4 ± 4.1

ΔH_A, enthalpy of association and ΔS_A, entropy of association. Determined at 293 K. In all cases, 1 to 1 stoichiometry per PrfA monomer was observed. Errors refer to independent repetitions (2–3) of experiments. Fitting errors not shown. See also Figures 5 and S3.

Table 2. Data Collection and Refinement Statistics

	$PrfA_{WT}$-**1**
Data Collection	
Space group	P21
Cell dimensions	
a, b, c (Å)	56.34, 80.97, 62.34
α, β, γ (°)	90.00, 112.54, 90.00
Resolution (Å)	57.58–2.25 (2.33–2.25)[a]
R_{merge}	0.058 (0.985)
R_{pim}	0.041 (0.698)
Wilson B factor (Å^2)	60.3
<I/σI>	12.1 (1.6)
Completeness (%)	99.2 (97.9)
Redundancy	5.6 (5.5)
CC1/2[b]	2.25 Å
Refinement	
Resolution (Å)	57.58–2.25 (2.33–2.25)
Number of reflections	24,400 (2,375)
R_{work}/R_{free}	0.203/0.254 (0.370/0.400)
Number of atoms	
Protein	3,738
Ligand	54
Water	22
B factors (Å^2)	
Protein	
Monomer A	86.6
Monomer B	83.6
Ligands	
**1** (AI)	65.5
**1** (BII)	70.1
Water	61.2
Root-mean-square deviation	
Bond lengths (Å)	0.016
Bond angles (°)	1.40
Ramachandran (%)	
Favored	98.0
Outliers	0.0
Clash score	3.21

[a]Data collected from one crystal.
[b]Suggested resolution cut-off (AIMLESS, see Supplemental Experimental Procedures). Values in parentheses are for highest-resolution shell.

(Figure 2D, 100 versus 10 μM in Figure 1A). A reduced accessibility of the compounds for PrfA after bacterial internalization or increased compound turn-over may have contributed to this, or the compounds may act more effectively at the step prior to PrfA activation (Figure 2D). The mechanism of intracellular PrfA activation has been postulated to involve a small molecule cofactor (Deshayes et al., 2012) that induces the ordering of the HTH motif evident in the $PrfA_{G145S}$ structure, thus enabling high-affinity interactions with consensus DNA sequences (Eiting et al., 2005). Recently it was shown that PrfA is more capable of initiating virulence gene expression if it is in a reduced state and bound to reduced glutathione (Reniere et al., 2015). Post infection, the inhibitory compounds are likely in competition with a cofactor, and PrfA activation may already have occurred, giving rise to a stable HTH motif. Consistent with this scenario is the reduced efficacy of compounds post infection (Figure 2D) and loss of efficacy against strains bearing the constitutively active $PrfA_{G145S}$ (Figure 4). In addition, both $PrfA_{WT}$- and $PrfA_{G145S}$-expressing strains were able to replicate efficiently in J774.1 macrophage-like cells after treatment with **2** (Figure S7), in line with the finding that maximal PrfA activity is not required for *L. monocytogenes* replication in bone marrow-derived macrophages (Reniere et al., 2015). Although our body of evidence clearly shows that the 2-pyridones interact with and inactivate PrfA, we do not completely rule out the possibility that they might also affect additional components contributing to *L. monocytogenes* virulence.

Other Crp/Fnr family members, such as Crp in *E. coli*, utilize cAMP binding for activation (Won et al., 2009). While the cAMP-binding site is topologically conserved within the N-terminal domain of PrfA, the residues involved in cAMP binding are not (Eiting et al., 2005). The crystal structure of the $PrfA_{WT}$-**1** complex shows that **1** does not bind at the conserved Crp cAMP-binding site (Figure S5B), nor to a solvent accessible cavity in the N-terminus of PrfA, recently proposed as a potential cofactor binding site (Deshayes et al., 2012). The described inhibitors are therefore unlikely to compete with a cofactor binding at those sites. The interdomain tunnel where **1** binds at site AI has previously been proposed as a possible effector site (Eiting et al., 2005), and our findings provide additional support for the importance of this region within an allosteric model of activation for PrfA. Further studies can clarify the relationship between glutathione as a substrate for activation and the discovered inhibitors.

Compound **1** came from a collection of molecules designed to block pilus assembly in *E. coli* through disrupting the chaperone-usher pathway (Emtenäs et al., 2002; Svensson et al., 2001). When used at a concentration of 3.6 mM, **1** blocked pilus biogenesis in *E. coli*, which raises the question of whether such effects may have been mediated through inhibition of Crp (Pinkner et al., 2006). The broad applicability of compounds based on **1** and **2** to inhibit other Crp/Fnr family members requires further investigation; however, several factors point against the favorability of

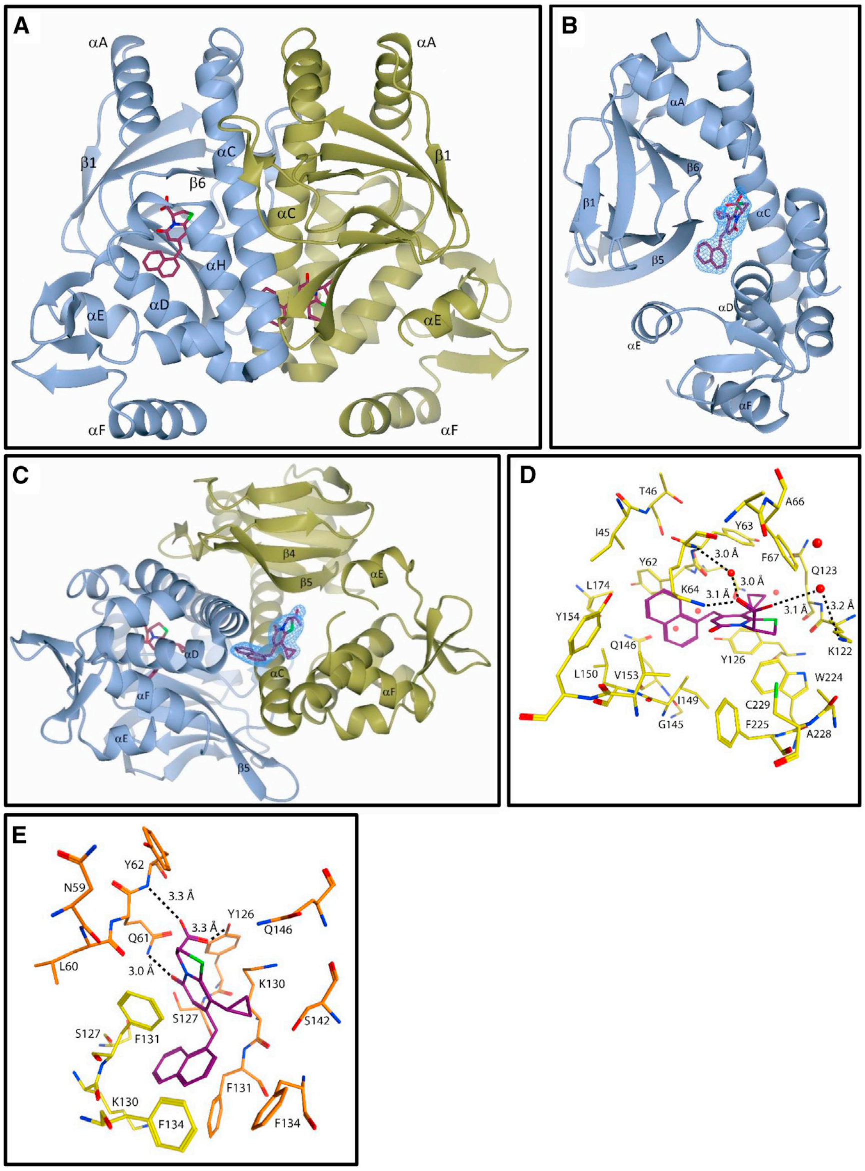

Figure 6. Structure of the $PrfA_{WT}$-1 Complex

(A) The $PrfA_{WT}$ homodimer showing the binding site of **1**. The monomeric units A and B of $PrfA_{WT}$ are colored in blue and gold, respectively. Two ligands bound to each monomer at two different sites, are shown as sticks.

(B and C) Quality of the electron density for compound **1** bound (A) to monomer A (site AI) and (C) to monomer B (site BII). The σ^A-weighted $(2m|F_o| - D|F_c|)$ electron density calculated from the refined PrfA-**1** complex is contoured at the root-mean-square deviation value of the map and shown in blue over compound **1** only.

(D) and (E) Key local structural features and amino acids in proximity to **1** at site AI (D) and BII (E). The ligand is colored by atom type: purple (C), green (S), blue (N), and red (O) and protein residues are colored by atom type: monomer A (C) yellow and monomer B (C) orange, green (S), blue (N), and red (O). Hydrogen bonds are indicated with dashed lines.

these compounds functioning as broad spectrum Crp/Fnr family inhibitors. At concentrations much higher than applied here, **1** had no effect in *E. coli* on the Crp-regulated pili expression (at 800 μM) (Cegelski et al., 2009; Chorell et al., 2010). At the protein level, PrfA differs structurally from Crp by the presence of three extra C-terminal helices (αG-αI) at the AI site (Eiting et al., 2005) and the BII site with its HTH motif is too compact to allow binding of **1** in Crp. Finally, at the bacterial level, the Gram-negative pathogen *E. coli* and Gram-positive *L. monocytogenes* are topologically distinct, which can have a profound impact on uptake and accessibility for small molecule inhibitors. The present work, however, provides a compelling proof-of-concept for investigating small molecule inhibitors of Crp family members as a means to disrupt the virulence of pathogenic bacteria.

Antivirulence drugs could provide a powerful, and potentially complementary treatment option to conventional antibiotics. Inhibitors of virulence in pathogenic bacteria, rather than inhibitors of viability, are now emerging, but only a few examples have been reported where the key regulator of virulence has been directly inactivated and none have provided structural details of the regulator-compound interaction (Garrity-Ryan et al., 2010; Hung et al., 2005; Shakhnovich et al., 2007; Sun et al., 2011; Yang et al., 2013). In the present work, we have identified a class of ring-fused 2-pyridones which attenuate *L. monocytogenes* virulence through inhibiting PrfA activation. These inhibitors represent excellent tool compounds for studying the virulence of this potentially fatal pathogen. Furthermore, the elucidated crystal structure of PrfA in complex with compound **1** enables improved ligand design, and provides a compelling rationale for the further investigation of Crp family members with small molecule inhibitors. Translating this research into a clinical application requires more effective inhibitors and in vivo proof-of-concept, but these findings represent the first steps in this process of identifying new therapeutic opportunities to treat infectious diseases.

SIGNIFICANCE

With increasing antibiotic resistance, new therapeutic strategies are needed to control bacterial infections, and virulence inhibition may afford opportunities for effective control. In this article, we have identified ring-fused 2-pyridones that attenuate the virulence of the Gram-positive bacterial pathogen *Listeria monocytogenes* by decreasing expression of central virulence genes. We identify the virulence regulator PrfA as the target of the compounds, and show how these inhibitors interact directly with PrfA to

reduce its DNA-binding capacity. The identified inhibitors are a new set of tool compounds for improving our understanding of *L. monocytogenes* virulence. We present the first crystal structure of a Crp/Fnr family member in complex with an inhibitor, and this affords new opportunities for designing ligands targeting this widespread family of bacterial transcriptional regulators.

EXPERIMENTAL PROCEDURES

Chemistry

All tested compounds were of ≥95% purity as determined by liquid chromatography-mass spectrometry. Compound **1** was synthesized as described previously (Chorell et al., 2010). Compounds **2–5** were synthesized by Suzuki-Miyaura cross-coupling with the chloromethyl ester **6** and subsequently hydrolyzed (Figure S8) (Chorell et al., 2010; Sellstedt et al., 2012). Detailed procedures and characterization are provided in the Supplemental Experimental Procedures.

Cell Lines, Bacterial Strains, and Growth Conditions

L. monocytogenes strains (EGDe, Mackaness, 1964; EGDe Δ*actA*, Tiensuu et al., 2013; EGDe Δ*prfA*, Toledo-Arana et al., 2009; EGDe Δ*hly*, Tiensuu et al., 2013; LO28, Vicente et al., 1989; 10403S, Myers et al., 1993; and F2365, Nelson et al., 2004) were grown in brain heart infusion (BHI) medium (Fluka) at 37°C with aeration (180 rpm), unless otherwise stated. For growth rate determination, samples were withdrawn every 30 min and optical density at 600 nm (OD_{600}) was measured. When required, erythromycin was used at a final concentration of 10 μg/ml. *E. coli* (DH5α; Bethesda Research Laboratories, 1986) was grown in Luria-Bertani medium at 37°C with agitation, unless otherwise stated. Antibiotics were, where appropriate, supplemented to final concentrations of 34 μg/ml for chloramphenicol and 100 μg/ml for kanamycin. HeLa cells were maintained in RPMI 1640 medium, whereas Caco-2 and J774 cells were maintained in DMEM, both supplemented with GlutaMAX Supplement (Gibco, Life Technologies) and 10% fetal bovine serum (Gibco, Life Technologies) at 37°C in a 5% CO_2 atmosphere.

Infection

L. monocytogenes EGDe, grown to an OD_{600} of 1.0, was pelleted, washed, and diluted in RPMI. DMSO or compound dissolved in DMSO was then added to the bacteria, resulting in a final concentration of 0.5% DMSO (v/v). The bacterial suspensions were added to the mammalian cells at a multiplicity of infection of approximately 10 and incubated for 1 hr at 37°C, before the cells were washed in PBS and incubated for 1.5 hr in cell medium supplemented with 50 μg/ml gentamycin. The cells were washed in PBS and subsequently lysed with PBS supplemented with 1% Triton X-100. The amount of intracellular bacteria was quantified by viable count of the cell lysate. For flow cytometry: after infection as above, the cells were washed in PBS and incubated for 7 hr in RPMI supplemented with 50 μg/ml gentamycin and subsequently analyzed using the Guava easyCyte (Millipore) flow cytometer. For intracellular protein levels, see Supplemental Experimental Procedures.

Western Blot

Overnight cultures of *L. monocytogenes* were diluted to an OD_{600} of 0.025 in BHI supplemented with DMSO, or the compounds dissolved in DMSO, resulting in a final concentration of 0.1% DMSO (v/v). The cultures were grown at 37°C to an OD_{600} of 1.0 before centrifugation (4,500 rpm, 10 min, 4°C). The supernatants and bacterial pellets were collected and subjected to SDS-PAGE and western blotting using specific antibodies. A detailed procedure is provided in the Supplemental Experimental Procedures.

Surface Plasmon Resonance

SPR experiments was conducted in a Biacore X100 (GE Healthcare), and performed mainly as described previously (Deshayes et al., 2012). In brief, biotinylated oligonucleotides (Promo40plcA-P14/Promo40plcAREV) containing the PrfA box of the *plcA* promoter were immobilized on one flow cell of an SA Chip (GE Healthcare). Assays were performed at 37°C. Protein samples were diluted in HBS-EP+ (GE Healthcare) to a final concentration of 200 nM, and compounds at different concentrations were added, resulting in a final DMSO concentration of 0.5%. The samples were injected with a flow rate of 10 μl/min. HBS-EP+ was used as running buffer. Measurements were done with a contact time of 200 s and a dissociation time of 120 s. This was followed by regeneration solution (0.1% SDS, 3 mM EDTA) with a contact time of 60 s. A flow cell without any immobilized DNA was used for reference subtraction. Each run was repeated three times.

Isothermal Titration Calorimetry

ITC experiments were performed using a MicroCal AutoiTC200 (GE Healthcare). In a typical run, 25 automated injections of 1.65 μl with 170-s breaks between injections were made at 25°C with 600 rpm stirring speed on low feedback mode. The protein concentrations in the sample cell were varied between 25 and 100 μM, while the compound concentration in the syringe was varied from 300 to 800 μM. The buffer for both protein and compound solutions was the same as in the final step of the purification. The compounds were dissolved in DMSO, and then diluted with buffer. DMSO content in the final compound solutions did not exceed 0.5% (v/v). Data integration, fitting, and evaluation were performed using the software Origin 7 with the ITC200 plugin provided by MicroCal/GE Healthcare.

Protein:Compound Crystallization and Data Collection

PrfA in complex with **1** was co-crystallized by the hanging-drop vapor-diffusion technique at 18°C. Crystals (0.1 × 0.4 × 0.02 mm^3) grew in 5 days when the protein solution (3.5 mg/ml PrfA, 0.75 mM **1**, 200 mM sodium chloride, 20 mM NaP [pH 6.5]) was mixed with an equal volume of mother liquor containing 20% PEG-4000, 16% isopropanol, and 100 mM sodium citrate (pH 5.5). Before data collection, the crystals were transferred to a cryo-protectant solution including 16% (v/v) glycerol in the precipitant solution. The crystals were flash-cooled to 100 K using a Cryostream 700 cooler (Oxford Cryosystems) and stored in liquid nitrogen. Diffraction data were collected at 100 K at the ESRF beamline ID29 (X-ray wavelength = 0.914 Å). The structure was solved with molecular-replacement methods. Data collection and refinement statistics are shown in Table 2. Details of the structure determination are provided in the Supplemental Information. The atomic coordinates and structure factors have been deposited (PDB: 5F1R) in the Research Collaboratory for Structural Bioinformatics (RCSB), Rutgers University, New Brunswick, NJ (http://www.rcsb.org/).

SUPPLEMENTAL INFORMATION

Supplemental Information includes Supplemental Experimental Procedures and eight figures and can be found with this article online at http://dx.doi.org/10.1016/j.chembiol.2016.02.013.

AUTHOR CONTRIBUTIONS

J.J., F.A., E.S.A., C.A., S.B., S.H., J.G., and P.W.S. wrote the manuscript. K.S.K., E.C., and J.G. synthesized and characterized the molecules. A.B., C.G., U.H.S., and E.S.E. determined the crystal structure and interpreted the data with J.J., F.A., and J.G. C.A., S.H., and J.W. performed western blot analysis and cell-infection experiments. J.W. and K.V. performed northern blot experiments. S.H. determined the hemolytic activity and growth rate. K.V. performed preliminary experiments. M.S.N. performed ITC and analyzed the data with P.W.S. C.A. performed and analyzed the SPR experiments. F.A. and J.J. conceived and initiated the project. All authors read and edited the manuscript.

ACKNOWLEDGMENTS

We are thankful to Johnny Mikaelsson for technical assistance. This project was funded by the Swedish Research Council (J.J., F.A., P.W.S., and E.S.E.), ERC starting grant no 260764–RNAntibiotics (J.J.), the Knut and Alice Wallenberg Foundation (P.W.S., E.S.A., J.J., and F.A.), the Göran Gustafsson Foundation (P.W.S. and F.A.), and the Swedish Foundation for Strategic Research (F.A.). K.S.K. thanks the JC Kempe Foundation for funding of his postdoctoral scholarship. We thank Drs. P. Cossart and C. O'Byrne for critically reading the manuscript. F.A. and J.J. are co-founders and co-owners of QureTech Bio AB.

Received: March 13, 2015
Revised: January 27, 2016
Accepted: February 19, 2016
Published: March 17, 2016

REFERENCES

Allen, R.C., Popat, R., Diggle, S.P., and Brown, S.P. (2014). Targeting virulence: can we make evolution-proof drugs? Nat. Rev. Microbiol. *12*, 300–308.

Andersson, C., Gripenland, J., and Johansson, J. (2015). Using the chicken embryo to assess virulence of *Listeria monocytogenes* and to model other microbial infections. Nat. Protoc. *10*, 1155–1164.

Bethesda Research Laboratories. (1986). BRL pUC host: *E. coli* DH5α competent cells. Focus *8*, 9.

Cegelski, L., Pinkner, J.S., Hammer, N.D., Cusumano, C.K., Hung, C.S., Chorell, E., Åberg, V., Walker, J.N., Seed, P.C., Almqvist, F., et al. (2009). Small-molecule inhibitors target *Escherichia coli* amyloid biogenesis and biofilm formation. Nat. Chem. Biol. *5*, 913–919.

Chakraborty, T., Leimeister-Wächter, M., Domann, E., Hartl, M., Goebel, W., Nichterlein, T., and Notermans, S. (1992). Coordinate regulation of virulence genes in *Listeria monocytogenes* requires the product of the prfA gene. J. Bacteriol. *174*, 568–574.

Chorell, E., Pinkner, J.S., Phan, G., Edvinsson, S., Buelens, F., Remaut, H., Waksman, G., Hultgren, S.J., and Almqvist, F. (2010). Design and synthesis of C-2 substituted thiazolo and dihydrothiazolo ring-fused 2-pyridones: pilicides with increased antivirulence activity. J. Med. Chem. *53*, 5690–5695.

Clatworthy, A.E., Pierson, E., and Hung, D.T. (2007). Targeting virulence: a new paradigm for antimicrobial therapy. Nat. Chem. Biol. *3*, 541–548.

Davies, J., and Davies, D. (2010). Origins and evolution of antibiotic resistance. Microbiol. Mol. Biol. Rev. *74*, 417–433.

Deshayes, C., Bielecka, M.K., Cain, R.J., Scortti, M., de las Heras, A., Pietras, Z., Luisi, B.F., Nunez Miguel, R., and Vazquez-Boland, J.A. (2012). Allosteric mutants show that PrfA activation is dispensable for vacuole escape but required for efficient spread and *Listeria* survival in vivo. Mol. Microbiol. *85*, 461–477.

Drevets, D.A., and Bronze, M.S. (2008). *Listeria monocytogenes*: epidemiology, human disease, and mechanisms of brain invasion. FEMS Immunol. Med. Microbiol. *53*, 151–165.

Eiting, M., Hageluken, G., Schubert, W.D., and Heinz, D.W. (2005). The mutation G145S in PrfA, a key virulence regulator of *Listeria monocytogenes*, increases DNA-binding affinity by stabilizing the HTH motif. Mol. Microbiol. *56*, 433–446.

Emtenäs, H., Åhlin, K., Pinkner, J.S., Hultgren, S.J., and Almqvist, F. (2002). Design and parallel solid-phase synthesis of ring-fused 2-pyridinones that target pilus biogenesis in pathogenic bacteria. J. Comb. Chem. *4*, 630–639.

Freitag, N.E., Rong, L., and Portnoy, D.A. (1993). Regulation of the prfA transcriptional activator of *Listeria monocytogenes*: multiple promoter elements contribute to intracellular growth and cell-to-cell spread. Infect. Immun. *61*, 2537–2544.

Freitag, N.E., Port, G.C., and Miner, M.D. (2009). *Listeria monocytogenes* - from saprophyte to intracellular pathogen. Nat. Rev. Microbiol. *7*, 623–628.

Garcia-Del Portillo, F., and Pucciarelli, M.G. (2012). Remodeling of the *Listeria monocytogenes* cell wall inside eukaryotic cells. Commun. Integr. Biol. *5*, 160–162.

Garrity-Ryan, L.K., Kim, O.K., Balada-Llasat, J.M., Bartlett, V.J., Verma, A.K., Fisher, M.L., Castillo, C., Songsungthong, W., Tanaka, S.K., Levy, S.B., et al. (2010). Small molecule inhibitors of LcrF, a *Yersinia* pseudotuberculosis transcription factor, attenuate virulence and limit infection in a murine pneumonia model. Infect. Immun. *78*, 4683–4690.

Greene, S.E., Pinkner, J.S., Chorell, E., Dodson, K.W., Shaffer, C.L., Conover, M.S., Livny, J., Hadjifrangiskou, M., Almqvist, F., and Hultgren, S.J. (2014). Pilicide ec240 disrupts virulence circuits in uropathogenic *Escherichia coli*. MBio *5*, e02038.

Gripenland, J., Andersson, C., and Johansson, J. (2014). Exploring the chicken embryo as a possible model for studying *Listeria monocytogenes* pathogenicity. Front. Cell. Infect. Microbiol. *4*, 170.

Hamon, M., Bierne, H., and Cossart, P. (2006). Listeria monocytogenes: a multifaceted model. Nat. Rev. Microbiol. *4*, 423–434.

Hung, D.T., Shakhnovich, E.A., Pierson, E., and Mekalanos, J.J. (2005). Small-molecule inhibitor of *Vibrio cholerae* virulence and intestinal colonization. Science *310*, 670–674.

Jackson, K.A., Iwamoto, M., and Swerdlow, D. (2010). Pregnancy-associated listeriosis. Epidemiol. Infect. *138*, 1503–1509.

Ladbury, J.E., Klebe, G., and Freire, E. (2010). Adding calorimetric data to decision making in lead discovery: a hot tip. Nat. Rev. Drug Discov. *9*, 23–27.

Levy, C., Pike, K., Heyes, D.J., Joyce, M.G., Gabor, K., Smidt, H., van der Oost, J., and Leys, D. (2008). Molecular basis of halorespiration control by CprK, a CRP-FNR type transcriptional regulator. Mol. Microbiol. *70*, 151–167.

Mackaness, G.B. (1964). The immunological basis of acquired cellular resistance. J. Exp. Med. *120*, 105–120.

Myers, E.R., Dallmier, A.W., and Martin, S.E. (1993). Sodium chloride, potassium chloride, and virulence in *Listeria monocytogenes*. Appl. Environ. Microbiol. *59*, 2082–2086.

Nelson, K.E., Fouts, D.E., Mongodin, E.F., Ravel, J., DeBoy, R.T., Kolonay, J.F., Rasko, D.A., Angiuoli, S.V., Gill, S.R., Paulsen, I.T., et al. (2004). Whole genome comparisons of serotype 4b and 1/2a strains of the food-borne pathogen *Listeria monocytogenes* reveal new insights into the core genome components of this species. Nucleic Acids Res. *32*, 2386–2395.

Pinkner, J.S., Remaut, H., Buelens, F., Miller, E., Aberg, V., Pemberton, N., Hedenstrom, M., Larsson, A., Seed, P., Waksman, G., et al. (2006). Rationally designed small compounds inhibit pilus biogenesis in uropathogenic bacteria. Proc. Natl. Acad. Sci. USA *103*, 17897–17902.

Portnoy, D.A., Auerbuch, V., and Glomski, I.J. (2002). The cell biology of *Listeria monocytogenes* infection: the intersection of bacterial pathogenesis and cell-mediated immunity. J. Cell Biol. *158*, 409–414.

Rasko, D.A., and Sperandio, V. (2010). Anti-virulence strategies to combat bacteria-mediated disease. Nat. Rev. Drug Discov. *9*, 117–128.

Reniere, M.L., Whiteley, A.T., Hamilton, K.L., John, S.M., Lauer, P., Brennan, R.G., and Portnoy, D.A. (2015). Glutathione activates virulence gene expression of an intracellular pathogen. Nature *517*, 170–173.

Ripio, M.T., Dominguez-Bernal, G., Lara, M., Suarez, M., and Vazquez-Boland, J.A. (1997). A Gly145Ser substitution in the transcriptional activator PrfA causes constitutive overexpression of virulence factors in *Listeria monocytogenes*. J. Bacteriol. *179*, 1533–1540.

Schnupf, P., and Portnoy, D.A. (2007). Listeriolysin O: a phagosome-specific lysin. Microbes Infect. *9*, 1176–1187.

Schultz, S., Shields, G., and Steitz, T. (1991). Crystal structure of a CAP-DNA complex: the DNA is bent by 90 degrees. Science *253*, 1001–1007.

Scortti, M., Monzo, H.J., Lacharme-Lora, L., Lewis, D.A., and Vazquez-Boland, J.A. (2007). The PrfA virulence regulon. Microbes Infect. *9*, 1196–1207.

Sellstedt, M., Krishna Prasad, G., Syam Krishnan, K., and Almqvist, F. (2012). Directed diversity-oriented synthesis. Ring-fused 5- to 10-membered rings from a common peptidomimetic 2-pyridone precursor. Tetrahedron Lett. *53*, 6022–6024.

Shakhnovich, E.A., Hung, D.T., Pierson, E., Lee, K., and Mekalanos, J.J. (2007). Virstatin inhibits dimerization of the transcriptional activator ToxT. Proc. Natl. Acad. Sci. USA *104*, 2372–2377.

Stritzker, J., Schoen, C., and Goebel, W. (2005). Enhanced synthesis of internalin A in aro mutants of *Listeria monocytogenes* indicates posttranscriptional control of the inlAB mRNA. J. Bacteriol. *187*, 2836–2845.

Suarez, M., Gonzalez-Zorn, B., Vega, Y., Chico-Calero, I., and Vazquez-Boland, J.A. (2001). A role for ActA in epithelial cell invasion by *Listeria monocytogenes*. Cell Microbiol. *3*, 853–864.

Sun, F., Zhou, L., Zhao, B.C., Deng, X., Cho, H., Yi, C., Jian, X., Song, C.X., Luan, C.H., Bae, T., et al. (2011). Targeting MgrA-mediated virulence regulation in *Staphylococcus aureus*. Chem. Biol. *18*, 1032–1041.

Svensson, A., Larsson, A., Emtenäs, H., Hedenström, M., Fex, T., Hultgren, S.J., Pinkner, J.S., Almqvist, F., and Kihlberg, J. (2001). Design and evaluation of pilicides: potential novel antibacterial agents directed against uropathogenic *Escherichia coli*. ChemBioChem *2*, 915–918.

Tiensuu, T., Andersson, C., Ryden, P., and Johansson, J. (2013). Cycles of light and dark co-ordinate reversible colony differentiation in *Listeria monocytogenes*. Mol. Microbiol. *87*, 909–924.

Toledo-Arana, A., Dussurget, O., Nikitas, G., Sesto, N., Guet-Revillet, H., Balestrino, D., Loh, E., Gripenland, J., Tiensuu, T., Vaitkevicius, K., et al. (2009). The *Listeria* transcriptional landscape from saprophytism to virulence. Nature *459*, 950–956.

Vázquez-Boland, J.A., Kuhn, M., Berche, P., Chakraborty, T., Domınguez-Bernal, G., Goebel, W., González-Zorn, B., Wehland, J., and Kreft, J. (2001). *Listeria* pathogenesis and molecular virulence determinants. Clin. Microbiol. Rev. *14*, 584–640.

Vega, Y., Rauch, M., Banfield, M.J., Ermolaeva, S., Scortti, M., Goebel, W., and Vazquez-Boland, J.A. (2004). New *Listeria monocytogenes* prfA* mutants, transcriptional properties of PrfA* proteins and structure-function of the virulence regulator PrfA. Mol. Microbiol. *52*, 1553–1565.

Vicente, M.F., Mengaud, J., Chenevert, J., Perez-Diaz, J.C., Geoffroy, C., Baquero, F., Cossart, P., and Berche, P. (1989). Reacquisition of virulence of haemolysin-negative *Listeria monocytogenes* mutants by complementation with a plasmid carrying the hlyA gene. Acta Microbiol. Hung. *36*, 199–203.

Won, H.S., Lee, Y.S., Lee, S.H., and Lee, B.J. (2009). Structural overview on the allosteric activation of cyclic AMP receptor protein. Biochim. Biophys. Acta *1794*, 1299–1308.

Xayarath, B., Volz, K.W., Smart, J.I., and Freitag, N.E. (2011). Probing the role of protein surface charge in the activation of PrfA, the central regulator of *Listeria monocytogenes* pathogenesis. PLoS One *6*, e23502.

Yang, J., Hocking, D.M., Cheng, C., Dogovski, C., Perugini, M.A., Holien, J.K., Parker, M.W., Hartland, E.L., Tauschek, M., and Robins-Browne, R.M. (2013). Disarming bacterial virulence through chemical inhibition of the DNA binding domain of an AraC-like transcriptional activator protein. J. Biol. Chem. *288*, 31115–31126.

Cell Chemical Biology, Volume *23*

Supplemental Information

Attenuating *Listeria monocytogenes* Virulence by Targeting the Regulatory Protein PrfA

James A.D. Good, Christopher Andersson, Sabine Hansen, Jessica Wall, K. Syam Krishnan, Afshan Begum, Christin Grundström, Moritz S. Niemiec, Karolis Vaitkevicius, Erik Chorell, Pernilla Wittung-Stafshede, Uwe H. Sauer, A. Elisabeth Sauer-Eriksson, Fredrik Almqvist, and Jörgen Johansson

Supplemental Information for:

Attenuating *Listeria monocytogenes* virulence by targeting the regulatory protein PrfA

James A. D. Good[a,b,1], Christopher Andersson[b,c,d,1], Sabine Hansen[b,c,d,1], Jessica Wall[b,c,d,1], K. Syam Krishnan[a,b,1], Afshan Begum[a,b], Christin Grundström[a,b], Moritz S. Niemiec[a], Karolis Vaitkevicius[b,c,d], Erik Chorell[a,b], Pernilla Wittung-Stafshede[a], Uwe H. Sauer[a,b], A. Elisabeth Sauer–Eriksson[a,b,2], Fredrik Almqvist[a,b,2] and Jörgen Johansson[b,c,d,2]

[a]Department of Chemistry, [b]Umeå Centre for Microbial Research (UCMR), [c]Department of Molecular Biology, [d]Molecular Infection Medicine, Sweden (MIMS),

Umeå University, 901 87 Umeå, Sweden.

[1]These authors contributed equally

[2]Corresponding authors: Jörgen Johansson: +46907852535, E-mail: jorgen.johansson@umu.se; Fredrik Almqvist: +46907866925, E-mail: Fredrik.almqvist@umu.se; Elisabeth Sauer-Eriksson: +46907865923, E-mail: Elisabeth.sauer-eriksson@umu.se

Present address: K.S.K: Department of Chemistry, Mannam Memorial NSS College, Kottiyam, Kollam, Kerala, India-691571; P.W.S.: Department of Biology and Bioengineering, Chalmers University of Technology, 41296 Gothenburg, Sweden.

Figure S1

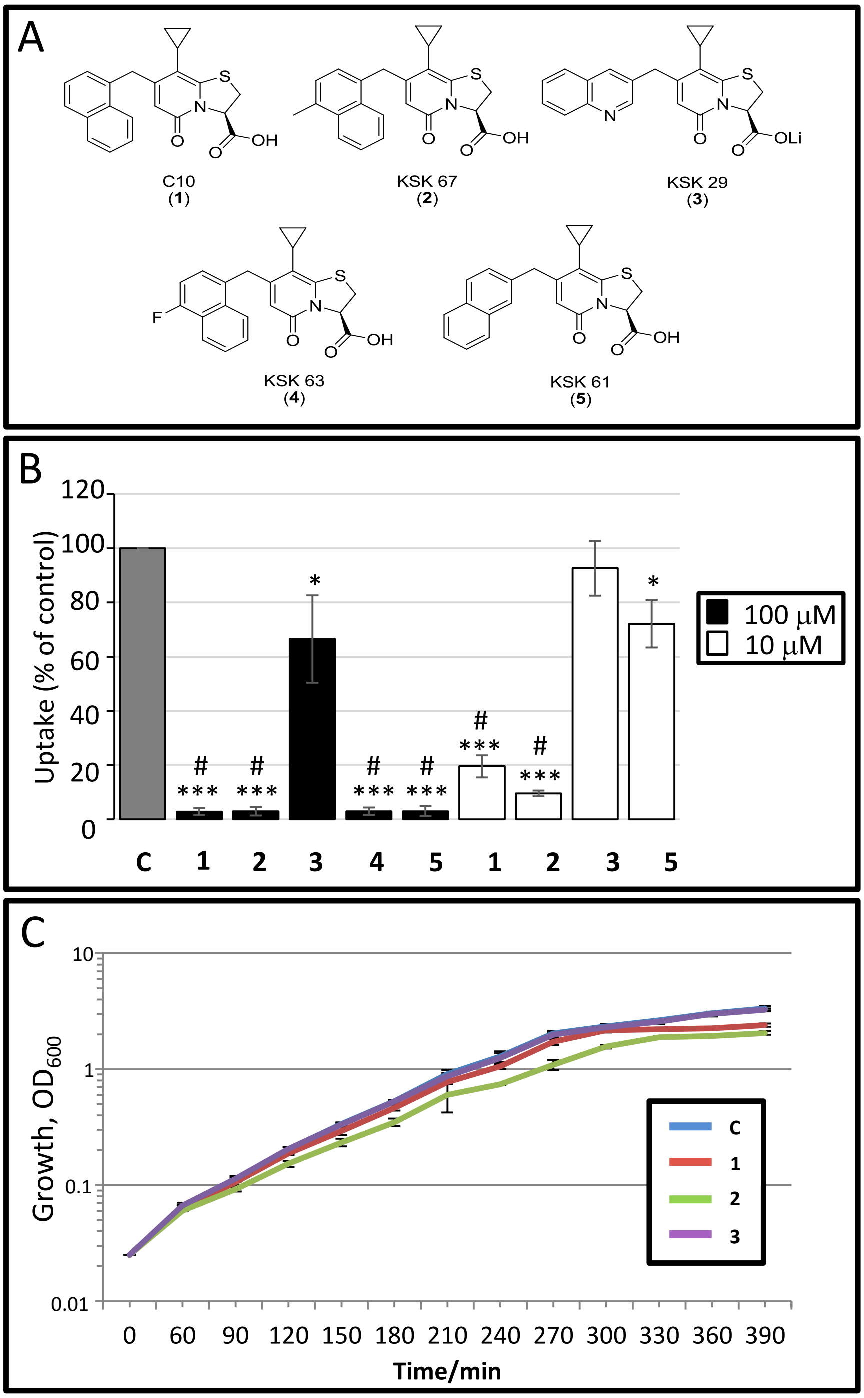

Figure S1. Infection inhibition and effect on growth by ring-fused 2-pyridines related to Figure 1. (A) Structures of bicyclic 2-pyridones used in this study. (B) Effect of bicyclic 2-pyridones on *L. monocytogenes* infectivity. GFP-expressing *L. monocytogenes* treated with compound at the indicated concentrations (100 μM, black bars; 10 μM, white bars) was allowed to infect HeLa cells for 7 hours, before analysis with a flow cytometer. The relative infection is calculated compared to the control (C; DMSO treated *L. monocytogenes*) (100%, grey bars). Values represent the mean ± standard deviation. n = 3. Significance was tested using Student's t-test (two-tailed - significant differences are shown by asterisks; * $p<0.05$ and *** $p<0.001$) and Dunnett's test (significant differences to the control are shown by #). (C) Growth of *L. monocytogenes* EGDe in brain heart infusion (BHI) medium + 0.1% DMSO (C - solvent); BHI + 100 μM **1**; **2** or **3**, respectively. Growth (n=4) was plotted as growth (OD_{600}) versus time (minutes). Error bars show standard deviation. The difference in growth between strains treated with compounds **1** or **2** compared with DMSO is statistically significant according to Student's t-test.

Figure S2

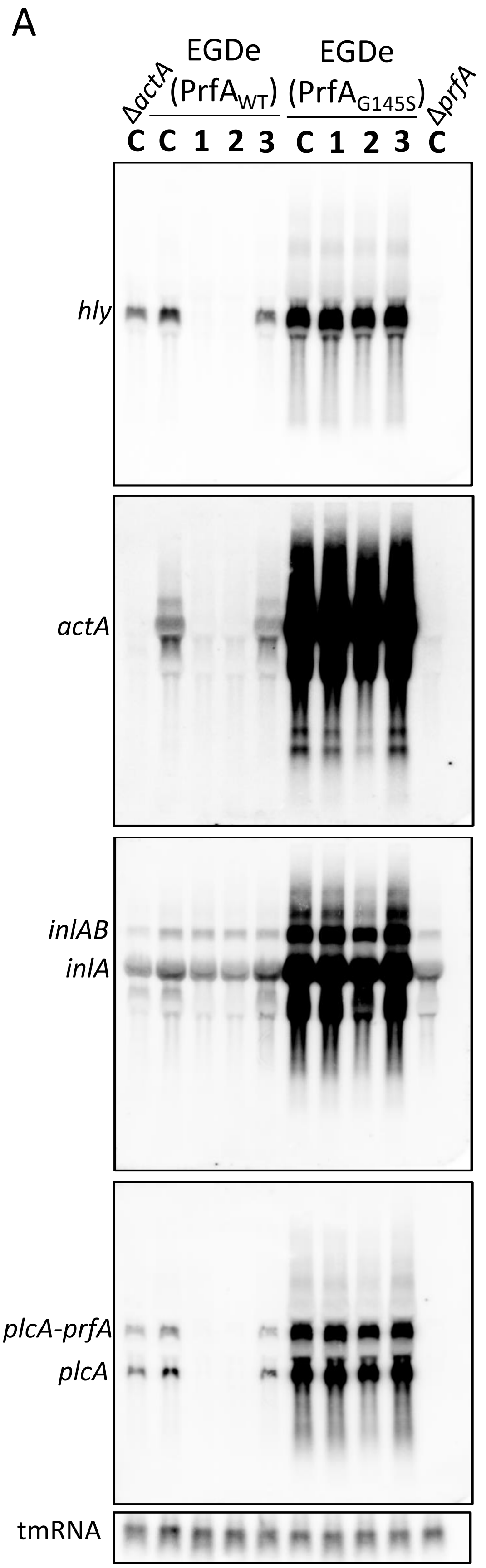

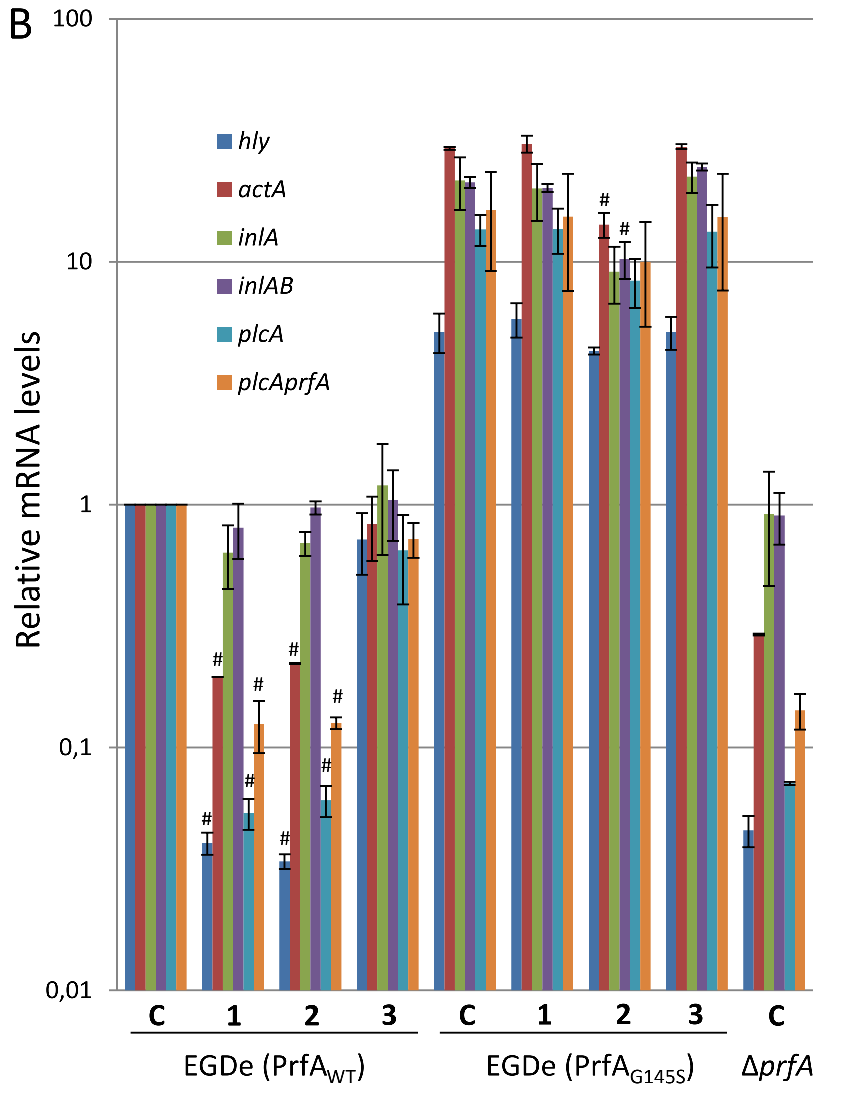

Figure S2. Effect of compounds on *L. monocytogenes* virulence gene expression related to Figure 2. (A) Total RNA was isolated from the indicated strains (Δ*actA*; EGDe (PrfA$_{WT}$); EGDe (PrfA$_{G145S}$) and Δ*prfA*, respectively) grown in BHI medium supplemented with DMSO (C) or compounds **1, 2** or **3** at 100 µM. Northern blot analysis was performed and membranes were hybridized with *hly*, *actA*, *inlA*, *plcA* or tmRNA specific DNA probes. Specific products are indicated at the left of the blots. (B) Relative levels of specific mRNA products identified in (a). All values were divided by the value of the wild-type strain (EGDe (PrfA$_{WT}$)) treated with DMSO, which was arbitrarily set to 1.0. Error bars show standard deviation. Significance was tested using Dunnett's test (significant differences to the control are shown by #). Values for samples from EGDe (PrfA$_{WT}$) treated strains were compared with the value of sample (EGDe (PrfA$_{WT}$) treated with DMSO, whereas samples from EGDe (PrfA$_{G145S}$) treated strains were compared with the value of sample (EGDe (PrfA$_{G145S}$) treated with DMSO.

Figure S3

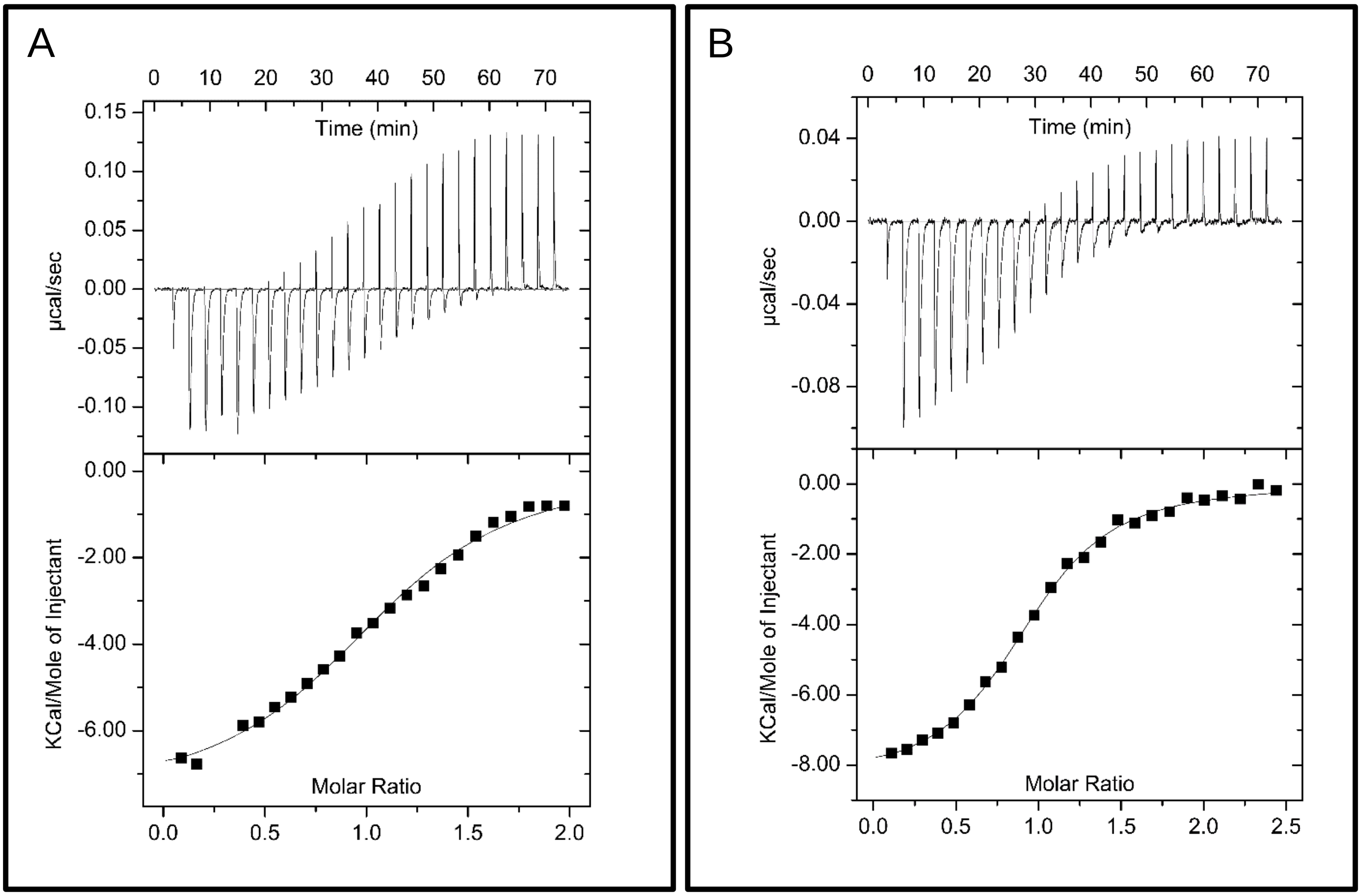

Figure S3. Isothermal titration calorimetry of compounds 2 (A) and 3 (B) to PrfA$_{WT}$, related to Figure 5. In each case small volumes of compound were injected to a solution of PrfA in the cell. The upper parts of the graphs show the measured heat after every injection, while the lower parts show integrated heat, normalized by amount and after background subtraction, as a function of molar ratio of compound to PrfA. Least-square fitting yields thermodynamic parameters that are given in Table 1.

Figure S4

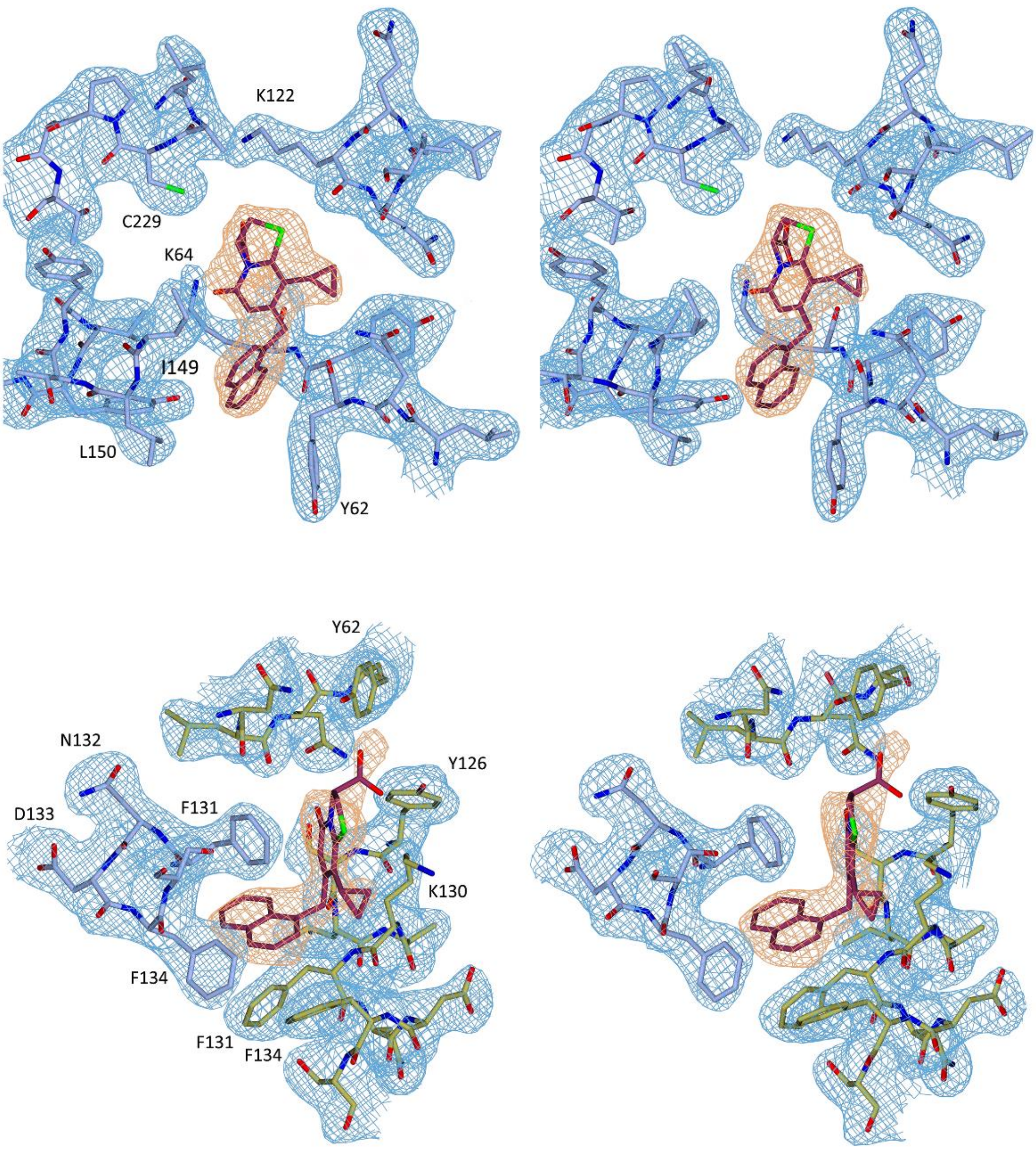

Figure S4. Validation of ligand binding, related to Figure 6. Related to Experimental Procedures. Upper panel: Stereo image showing the electron density quality of **1** bound to monomer A at site AI of the PrfA dimer. Lower panel: Stereo image showing the electron density quality of **1** bound to monomer B at site BII of the PrfA dimer. The σ^A-weighted (2m|Fo|-D|Fc|) electron density map calculated with phases from the refined structure is contoured at one root-mean-square deviation (RMSD) value of the map, and shown in blue. The σ^A-weighted (m|Fo|-D|Fc|) electron density, contoured at 3 times the RMSD value is shown in orange. To avoid model bias compound **1** was excluded from the coordinate file that was subjected to one round of refinement before calculation of the |Fo|-|Fc| electron density map. Shown is the electron density covering **1** and selected residues of the binding sites including amino acids A-Leu60-Lys64, A-Leu120-Val124, A-Ile149-Gly155 and A-Leu227-Thr232 at site AI and amino acids A-Phe131-Ser135, B-Asn59-Tyr62, and B-Tyr126-Ser135 at site BII. In both binding sites compound 1 is well defined in the non-biased (m|Fo|-D|Fc|) electron density map.

Figure S5

A

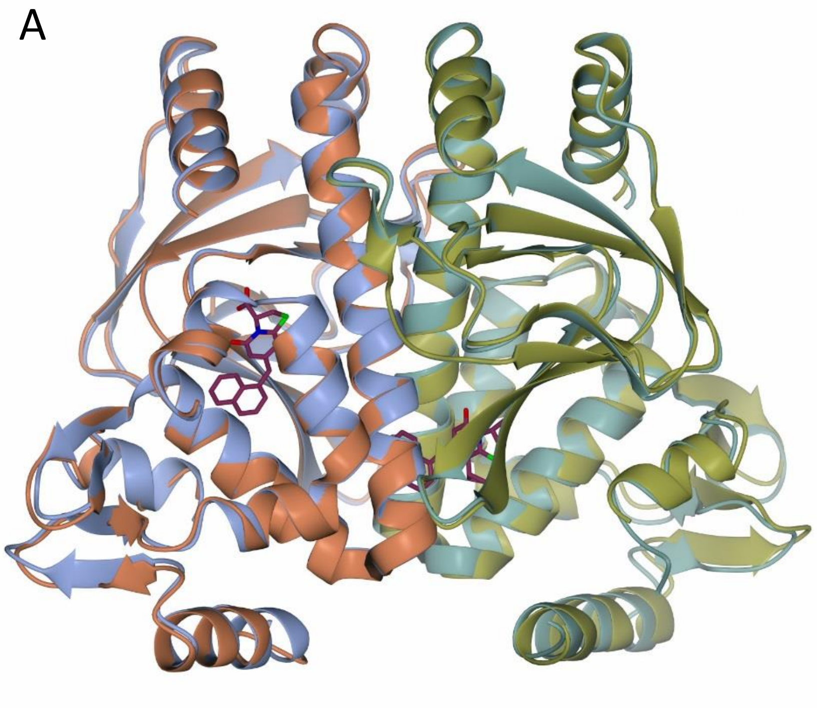

Figure S5. PrfA$_{WT}$ and PrfA$_{WT}$:1 complex structures are similar and compound 1 and cAMP occupy different binding sites, related to Figure 6. (A) Superposition of monomer A of the PrfA$_{WT}$:**1** complex structure (monomer A, blue; monomer B, gold) onto monomer A of the PrfA$_{WT}$ structure (monomer A, orange; monomer B, sea-green, PDB entry 2beo, (Eiting et al., 2005)) shows that their 3D structures are similar. Superimposition of the two structures results in an RMSD of 0.9-1.0 Å. This demonstrates that binding of **1** to PrfA$_{WT}$ has a negligible effect on the overall structure of the dimer. The ligands are colored by atom type: purple (C), green (S), blue (N) and red (O). (B) Superposition of the PrfA:1 (blue and green) and Crp:cAMP complexes (gray, pdb-code 1g6n, (Passner et al., 2000)) based on monomer A only. The closest distances between cAMP and compound 1 at sites AI and BII are ~4 Å and ~5 Å, respectively.

B

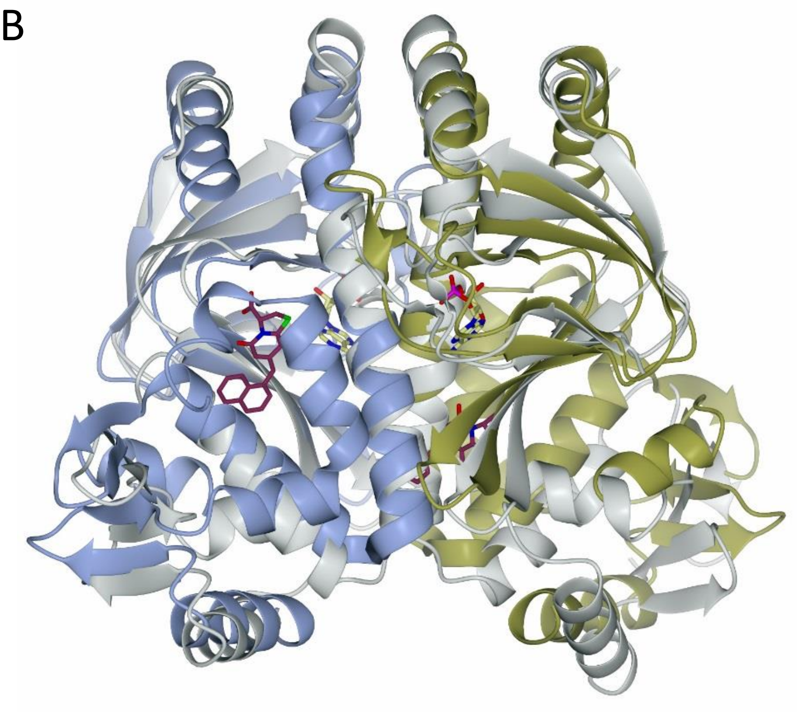

Figure S6

A

B

C

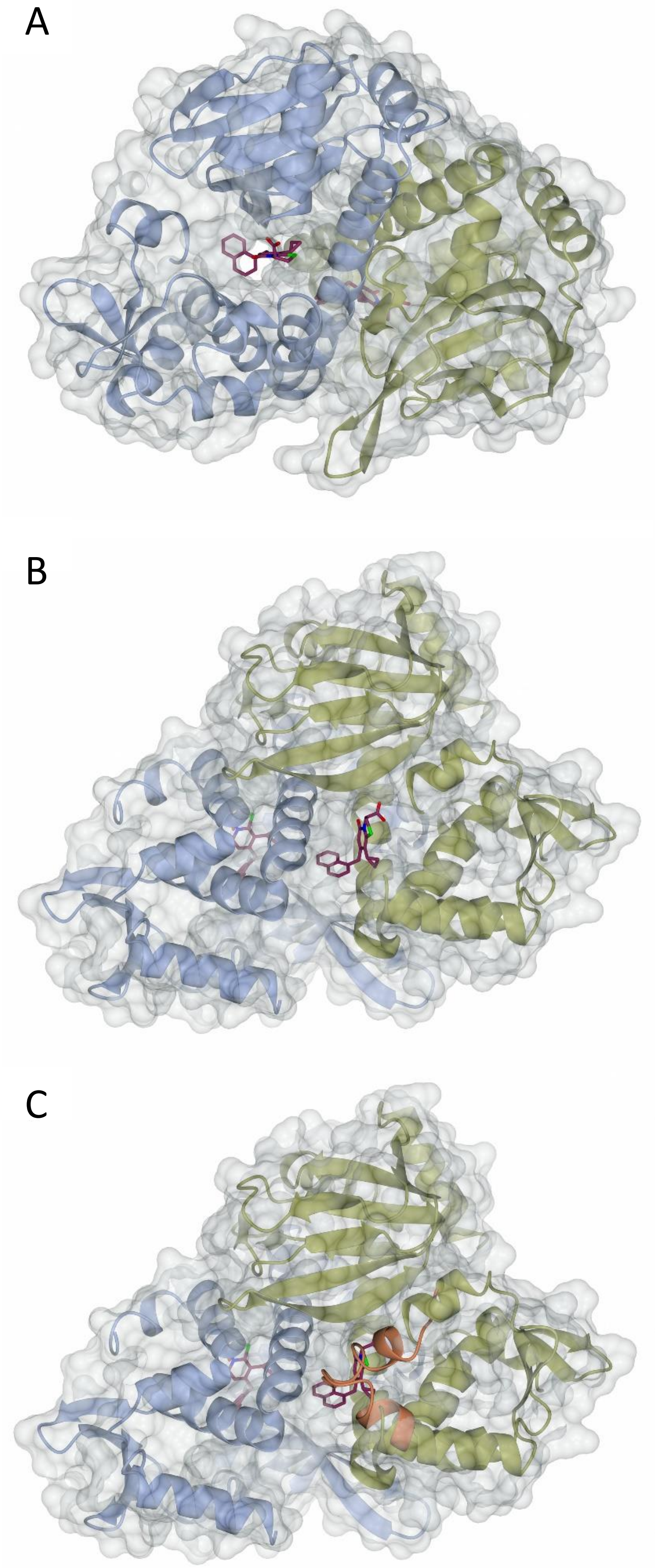

D

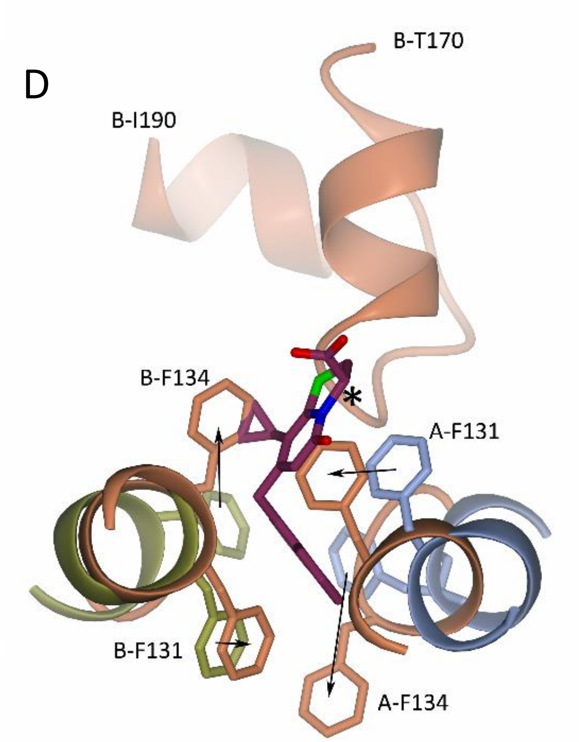

Figure S6. Compound 1 binds at two areas of PrfA, related to Figure 6. (A) Binding within the interdomain tunnel in between helices αC, αD, αH, αI and β-strand β5 (site AI). (B) Binding of **1** at a pocket in between helices αC, αD, and β5 and in vicinity of the in the wild-type structure not folded HTH motif (site BII). (C) The same figure as in b including the HTH motif (residues 168-190, orange ribbon) from the superimposed $PrfA_{G145S}$ structure (pdb code 2bgc). (D) Close-up view of the BII site. Superimposed residues from the $PrfA_{G145S}$ structure are shown in orange. The naphthyl group of compound 1 is sandwiched between residues Phe131 and Phe134 from both monomers. This position of the naphthyl group prevents conformational changes needed for HTH formation. Furthermore, residues in the turn of the HTH motif, highlighted here with *, are positioned in close vicinity, 2.5-3.0 Å, from the 2-pyridone ring.

Figure S7

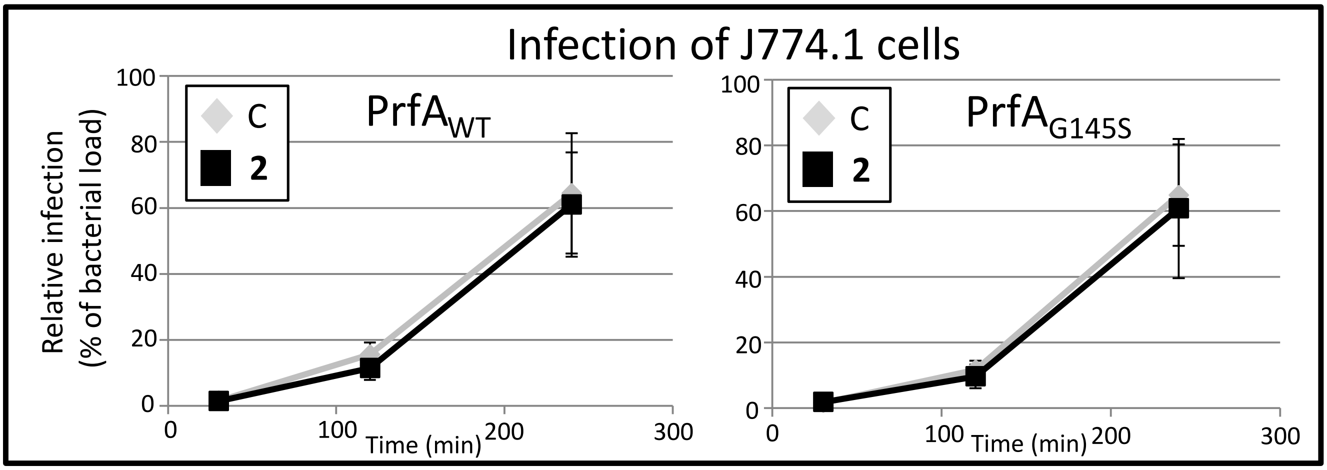

Figure S7. Time-course infection dynamics of *L. monocytogenes* harboring PrfA$_{WT}$ or PrfA$_{G145}$ after treatment with compound 2, related to Figure 1. J774.1 cells were infected with *L. monocytogenes* strains carrying PrfA$_{WT}$ (left panel) or PrfA$_{G145S}$ (right panel) in the presence of DMSO (C) or compound **2** (50 µM). The amount of intracellular bacteria were measured using viable count at indicated time-points post infection (p.i.) and divided by the bacterial load used in the infection (prior to antibiotic treatment) and set as %. n=3. Significance was tested using Student's t-test and Dunnett's test (No difference was significant.)

Figure S8

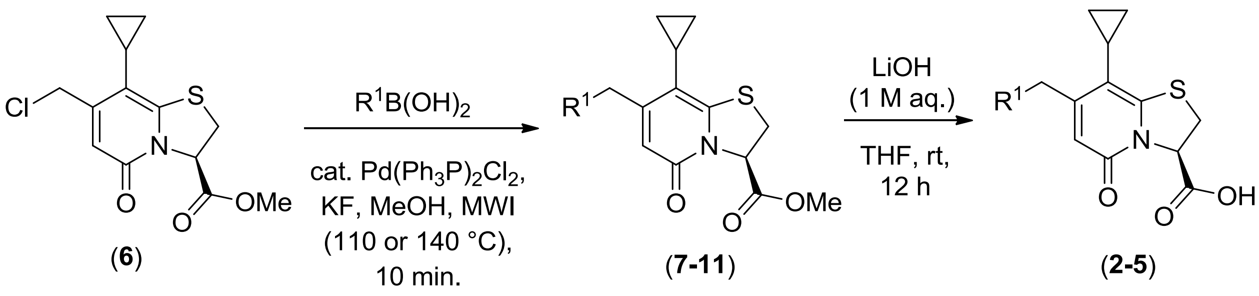

(**6**) (**7-11**) (**2-5**)

Figure S8. Synthesis of R-7 substituted thiazolino 2-pyridone derivatives, related to Figure 1.

Supplemental Experimental Procedures

Chemistry

General

All reagents and solvents were used as received from commercial suppliers. TLC was performed on aluminum backed silica gel plates (median pore size 60 Å) and detected with UV light at 254 nm. Column chromatography was performed using silica gel with average particle diameter 50 µM (range 40-65 µM, pore diameter 53 Å) and eluents are given in brackets. Optical rotation was measured with a polarimeter at 25 °C at 589 nm. IR spectra were recorded on a spectrometer equipped with an ATR device. ^{1}H and ^{13}C NMR spectra were recorded on a 400 MHz spectrometer at 298 K and calibrated by using the residual peak of the solvent as the internal standard ($CDCl_3$: $\delta_H = 7.26$ ppm; $\delta_C = 77.16$ ppm; DMSO-d_6: $\delta_H = 2.50$ ppm; $\delta_C = 39.50$ ppm). HRMS was performed by using a mass spectrometer with ESI-TOF (ESI+); sodium formate was used as the calibration chemical. HPLC purifications were performed on a system equipped with a 250 × 21.5 mm Nucleodur® C18 HTEC (particle size 5 µM) column using a flow rate of 20 mL/min and detection at 220 nm. Compound **1** was synthesized as described previously (Chorell et al., 2010).

General procedure for Suzuki-Miyaura cross-couplings

The chloromethyl derivative **6** (1.0 eq.), boronic acid (2.5 eq.), $Pd(Ph_3P)_2Cl_2$ (0.1 eq.) and KF (2.5 eq.) were dissolved in MeOH (10 mL) and heated by microwave irradiation (MWI) at 110 or 140 °C for 10 min. The reaction mixture was diluted with saturated aqueous $NaHCO_3$ and extracted with ethyl acetate. The organic layer was successively washed with water and brine,

dried (Na_2SO_4) and concentrated under reduced pressure. Purification by flash chromatography (SiO_2; EtOAc/heptane; 30-100%) afforded the cross-coupled product.

General methyl ester hydrolysis procedure

Methyl carboxylates **7-10** were hydrolysed by adaption of a previously reported procedure (Chorell et al., 2010). LiOH (1 M aqueous) was added drop wise to a stirred solution of methyl ester substituted 2-pyridone in THF (30 mL/mmol) at 0 °C. The solution was allowed to attain room temperature and was stirred for 12 h. The mixture was diluted with CH_2Cl_2 and acidified with aqueous HCl (1 M) to *circa* pH 1. The separated organic layer was dried (Na_2SO_4), and concentrated under reduced pressure. The residue was triturated with diethyl ether (3×) and lyophilized (H_2O:MeCN; ~ 8:2) to afford the carboxylic acid.

Methyl (3*R*)-8-cyclopropyl-7-[(4-methylnaphthalen-1-yl)methyl]-5-oxo-2,3-dihydro-5*H*-[1,3]thiazolo[3,2-*a*]pyridine-3-carboxylate (7). Following the general procedure with **6** (138 mg, 0.46 mmol) and 4-methyl-1-naphthaleneboronic acid (128 mg, 0.69 mmol) and MWI at 140 °C gave **7** (153 mg, 82%) as a white solid. ^{1}H NMR ($CDCl_3$, 400 MHz) δ = 8.01 (d, 1H, *J* = 7.6 Hz), 7.77 (d, 1H, *J* = 7.9 Hz), 7.59-7.52 (m, 2H), 7.33 (d, 1H, *J* = 7.2 Hz), 7.25 (d, 1H, *J* = 7.1 Hz), 6.00 (s, 1H), 5.54 (dd, 2H, J = 2.2, 8.6 Hz), 4.42 (d, 1H, *J* = 17.2 Hz), 4.32 (d, 1H, *J* = 17.2 Hz), 3.94 (s, 3H), 3.62 (dd, 2H, J = 8.6, 11.6), 3.50 (dd, 1H, *J* = 2.2, 11.6 Hz), 2.71 (s, 3H), 1.45 -1.35 (m, 1H), 0.97-0.87 (m, 2H), 0.70-0.61 (m, 2H); ^{13}C NMR ($CDCl_3$, 100 MHz) δ = 170.6, 160.1, 156.9, 148.0, 133.7, 133.1, 133.0, 132.2, 127.6, 126.4, 126.3, 126.2, 125.4, 125.1, 113.5, 112.6, 62.8, 55.3, 39.2, 31.5, 20.3, 11.3, 8.0, 7.7. HRMS (ESI+) (*m/z*): $[M]^+$ calcd. for $C_{24}H_{24}NO_3S^+$, 406.1471; found, 406.1460.

Methyl (3*R*)-8-cyclopropyl-5-oxo-7-(quinolin-3-ylmethyl)-2,3-dihydro-5*H*-[1,3]thiazolo[3,2-*a*]pyridine-3-carboxylate (8). The chloromethyl derivative **6** (175 mg, 0.58 mmol), 3-quinolineboronic acid pinacol ester (298 mg, 1.17 mmol), $PdCl_2(PPh_3)_2$ (21 mg, 0.03 mmol) and KF (68 mg, 1.17 mmol) were dissolved in anhydrous MeOH/MeCN (3.5 mL; 1:5) and heated by MWI at 110 °C for 10 min. The reaction mixture was quenched with saturated aqueous $NaHCO_3$ solution (10 mL) and extracted with EtOAc (3 × 10 mL). The combined organic extracts were washed successively with saturated aqueous $NaHCO_3$ solution, water (2×) and brine (10 mL each), dried (Na_2SO_4) and concentrated under reduced pressure. Purification by flash chromatography (SiO_2; MeOH/CH_2Cl_2; 0-20%) afforded the product as a pale yellow solid (174 mg, 76%). ^{1}H NMR ($CDCl_3$, 400 MHz) δ = 8.79 (d, 1H, *J* = 2.0 Hz), 8.11 (s, 1H), 8.02 (d, 1H, *J* = 8.0 Hz), 7.98 (d, 1H, *J* = 8.2 Hz), 7.72 (t, 1 H, *J* = 7.4 Hz), 7.58 (t, 1H, *J* = 7.4 Hz), 6.01 (s, 1H), 5.55-5.50 (m, 1H), 4.18 (d, 1H, *J* = 15.6 Hz), 4.12 (d, 1H, *J* = 15.6 Hz), 3.52-3.47 (m, 2H), 1.43-1.33 (m, 1H), 0.95-0.87 (m, 2H), 0.63-0.51 (m, 2H). ^{13}C NMR ($CDCl_3$, 100 MHz) δ = 170.3, 160.9, 154.3, 152.8, 149.9, 146.8, 135.6, 132.9, 129.9, 129.3, 128.2, 128.1, 127.2, 115.2, 111.9, 66.6, 56.1, 39.1, 31.6, 11.4, 8.4, 7.9. HRMS (ESI+) (*m/z*): $[M]^+$ calcd. for $C_{22}H_{21}N_2O_3S^+$, 393.1267; found, 393.1260.

Methyl (3*R*)-8-cyclopropyl-7-[(4-fluoronaphthalen-1-yl)methyl]-5-oxo-2,3-dihydro-5*H*-[1,3]thiazolo[3,2-*a*]pyridine-3-carboxylate (9). Following the general procedure, **6** (540 mg, 1.8 mmol, MWI at 140 °C) gave **9** (540 mg, 73%) as pale yellow solid. ^{1}H NMR ($CDCl_3$, 400 MHz) δ = 8.12-8.09 (m, 1H), 7.94-7.91 (m, 1H), 7.68-7.64 (m, 2H), 7.39-7.34 (m, 2H), 5.99 (s, 1H), 5.56 (dd, 1H, *J* = 2.6, 9.2 Hz), 4.44 (d, 1H, *J* = 17.6 Hz), 4.34 (d, 1H, *J* = 17.6 Hz), 3.95 (s, 3H), 3.74 (dd, 1H, *J* = 9.2, 11.6 Hz), 3.53 (dd, 1H, *J* = 2.6, 11.6 Hz), 1.49 -1.41 (m, 1H),

0.92-0.87 (m, 2H), 0.74-0.64 (m, 2H). ^{13}C NMR ($CDCl_3$, 100 MHz) δ = 169.9, 159.8, 157.3 (d, J_{CF} = 248 Hz), 155.9, 148.3, 132.8 (d, J_{CF} = 5.2 Hz), 130.9 (d, J_{CF} = 4 Hz), 127.8, 127.4, 126.5, 124.6, 123.0 (d, J_{CF} = 16.3 Hz), 120.5 (d, J_{CF} = 5 Hz), 113.6, 111.9, 109.2 (d, J_{CF} = 20 Hz), 63.3, 55.9, 39.0, 31.9, 10.7, 7.8, 7.3. HRMS (ESI+) (*m/z*): $[M]^+$ calcd. for $C_{23}H_{21}FNO_3S^+$, 410.1221; found, 410.1204.

Methyl (3*R*)-8-cyclopropyl-7-(naphthalen-2-ylmethyl)-5-oxo-2,3-dihydro-5*H*-[1,3]thiazolo[3,2-*a*]pyridine-3-carboxylate (10). Following the general procedure, **6** (360 mg, 1.2 mmol, MWI at 110 °C) gave **10** (358 mg, 76%) as pale yellow solid. ^{1}H NMR ($CDCl_3$, 400 MHz) δ = 8.11-8.08 (m, 1H), 7.98-7.95 (m, 1H), 7.69-7.65 (m, 2H), 7.53 (s, 1H), 7.37-7.32 (m, 2H), 6.06 (s, 1H), 5.56 (dd, 1H, *J* = 2.2, 9.2 Hz), 4.44 (d, 1H, *J* = 17.4 Hz), 4.33 (d, 1H, *J* = 17.4 Hz), 3.89 (s, 3H), 3.61 (dd, 1H, *J* = 9.2, 11.6 Hz), 3.46 (dd, 1H, *J* = 2.2, 11.6 Hz), 1.46 -1.37 (m, 1H), 0.93-0.80 (m, 2H), 0.77-0.59 (m, 2H). ^{13}C NMR ($CDCl_3$, 100 MHz) δ = 168.7, 161.3, 157.5, 157.0, 148.4, 133.7, 133.0, 132.1, 129.1, 128.8, 127.5, 127.1, 119.2, 115.7, 113.4, 107.8, 62.7, 55.3, 39.3, 31.9, 11.2, 7.9, 7.7. HRMS (ESI+) (*m/z*): $[M]^+$ calcd. for $C_{23}H_{22}NO_3S^+$, 392.1315; found, 392.1304.

(3*R*)-8-Cyclopropyl-7-(4-methyl-naphthalen-1-ylmethyl)-5-oxo-2,3-dihydro-5*H*-thiazolo[3,2-*a*]pyridine-3-carboxylic acid (2). Following the general procedure for hydrolysis with **7** (45 mg, 0.11 mmol) gave **2** (33 mg, 76%) as a white solid. $[\alpha]_D$ **=** -16 (*c* = 0.5, DMSO). IR (υ cm^{-1}) (neat) 1725, 1619, 1487, 1391, 1169. ^{1}H NMR (DMSO-d_6, 400 MHz) δ = 8.05 (d, 1H, *J* = 7.6 Hz), 7.86 (d, 1H, *J* = 8.0 Hz), 7.58-7.50 (m, 2H), 7.33 (d, 1H, *J* = 7.2 Hz), 7.25 (d, 1H, *J* = 7.2 Hz), 5.21-5.19 (m, 2H), 4.43 (d, 1H, *J* = 17.2 Hz), 4.33 (d, 1H, *J* = 17.2 Hz), 3.68-3.62 (m, 1H), 3.50 (d, 1H, *J* = 11.2 Hz), 2.66 (s, 3H), 1.72 -1.67 (m, 1H), 0.93-

0.85 (m, 2H), 0.74-0.70 (m, 1H), 0.65-0.60 (m, 1H). ^{13}C NMR (DMSO-d_6, 100 MHz) δ = 170.1, 160.4, 157.1, 148.4, 133.7, 133.1, 133.0, 132.1, 127.8, 126.8, 126.5, 126.2, 125.3, 125.1, 113.9, 112.4, 62.8, 35.8, 31.7, 19.6, 11.2, 7.9, 7.7. HRMS (ESI+) *m/z*: $[M+Na]^+$ calcd. for $C_{23}H_{21}NO_3S$, 414.1140; found, 414.1126.

Lithium (3*R*)-8-cyclopropyl-7-(isoquinolin-3-ylmethyl)-5-oxo-2,3-dihydro-5*H*-[1,3]thiazolo[3,2-*a*]pyridine-3-carboxylate (3). Aqueous LiOH (0.1 M, 0.71 mL, 0.071 mmol) was added drop wise to a stirred solution of methyl ester **8** (28 mg, 0.071 mmol) in THF (3 mL) at 0 °C. The solution was allowed to attain room temperature and stirred for 12 h, concentrated and purified by HPLC [mobile phase: MeCN/H_2O, (10-60 % for 30 min); t_R = 11.98 min]. Subsequent lypholization of the collected fractions (H_2O:MeCN; ~ 8:2) gave the lithium carboxylate **3** (20 mg, 73 %) as a pale yellow solid. $[\alpha]_D$ = -3 (*c* = 0.1, MeOH). IR (υ cm^{-1}) (neat) 1624, 1557, 1487, 1397, 1291, 1175. ^{1}H NMR (DMSO-d_6, 400 MHz) δ = 8.81 (d, 1H, *J* = 2.0 Hz), 8.15 (s, 1H), 8.00 (d, 1H, *J* = 8.4 Hz), 7.94 (d, 1H, *J* = 8.0 Hz), 7.72 (t, 1 H, *J* = 7.4 Hz), 7.58 (t, 1H, *J* = 7.4 Hz), 5.70 (s, 1H), 5.05-5.02 (m, 1H), 4.19 (d, 1H, *J* = 15.6 Hz), 4.13 (d, 1H, *J* = 15.6 Hz), 3.52 (d, 2H, *J* = 4.8 Hz), 1.40 -1.33 (m, 1H), 0.95-0.88 (m, 1H), 0.83-0.78 (m, 1H), 0.63-0.51 (m, 2H). ^{13}C NMR (DMSO-d_6, 100 MHz) δ = 169.1, 160.8, 154.3, 152.6, 149.9, 146.9, 135.4, 132.7, 129.5, 129.1, 128.2, 128.1, 127.2, 114.7, 111.2, 66.1, 35.9, 33.6, 11.3, 8.3, 7.8. HRMS (ESI+) (*m/z*): $[M-Li]^+$ calcd. for $C_{21}H_{19}N_2O_3S^+$, 379.1111; found, 379.1141.

(3*R*)-8-Cyclopropyl-7-(4-fluoro-naphthalen-1-ylmethyl)-5-oxo-2,3-dihydro-5*H*-thiazolo[3,2-*a*]pyridine-3-carboxylic acid (4). Following the general procedure for hydrolysis with **9** (250 mg, 0.61 mmol) gave **4** (202 mg, 84%) as a white solid. $[\alpha]_D$ = -5.8 (*c*

= 0.5, $CHCl_3$:MeOH (9:1)). IR (υ cm^{-1}) (neat) 1726, 1618, 1487, 1423, 1392, 1201, 1171. ^{1}H NMR (DMSO-d_6, 400 MHz) δ = 8.13-8.10 (m, 1H), 7.95-7.92 (m, 1H), 7.67-7.64 (m, 2H), 7.37-7.31 (m, 2H), 5.25 (d, 1H, *J* = 8.8 Hz), 5.23 (s, 1H), 4.46 (d, 1H, *J* = 17.6 Hz), 4.37 (d, 1H, *J* = 17.6 Hz), 3.72 (dd, 1H, *J* = 9.2, 11.6 Hz), 3.51 (d, 1H, *J* = 11.6 Hz), 1.74 -1.67 (m, 1H), 0.93-0.86 (m, 2H), 0.77-0.71 (m, 1H), 0.66-0.60 (m, 1H). ^{13}C NMR (DMSO-d_6, 100 MHz) δ = 169.7, 159.9, 157.1 (d, J_{CF} = 248 Hz), 155.6, 148.3, 132.7 (d, J_{CF} = 5 Hz), 130.9 (d, J_{CF} = 4 Hz), 127.5, 127.3, 126.5, 124.5, 123.1 (d, J_{CF} = 16 Hz), 120.4 (d, J_{CF} = 5 Hz), 113.3, 111.5, 109.2 (d, J_{CF} = 20 Hz), 63.3, 34.8, 31.7, 10.7, 7.4, 7.1. HRMS (ESI+) *m/z*: $[M+Na]^+$ calcd. for $C_{22}H_{18}FNO_3S$, 418.0889; found, 418.0870.

(3*R*)-8-cyclopropyl-7-(naphthalen-2-ylmethyl)-5-oxo-2,3-dihydro-5*H*-[1,3]thiazolo[3,2-*a*]pyridine-3-carboxylic acid (5). Following the general procedure for hydrolysis with **10** (60 mg, 0.15 mmol) gave **5** (53 mg, 92%) as a white solid. $[\alpha]_D$ **=** -17.5 (*c* = 0.2, DMSO). IR (υ cm^{-1}) (neat) = 1702, 1616, 1486, 1174. ^{1}H NMR (DMSO, 400 MHz) δ = 8.13-8.10 (m, 1H), 7.95-7.92 (m, 1H), 7.67-7.64 (m, 2H), 7.54 (s, 1H), 7.37-7.31 (m, 2H), 5.25 (d, 1H, *J* = 8.8 Hz), 5.23 (s, 1H), 4.46 (d, 1H, *J* = 17.6 Hz), 4.37 (d, 1H, *J* = 17.6 Hz), 3.72 (dd, 1H, *J* = 9.2, 11.6 Hz), 3.51 (d, 1H, *J* = 11.6 Hz), 1.74 -1.67 (m, 1H), 0.93-0.86 (m, 2H), 0.66-0.60 (m, 2H); ^{13}C NMR (DMSO, 100 MHz) δ = 169.7, 160.1, 157.0, 156.6, 148.5, 133.6, 133.1, 129.1, 128.7, 127.3, 126.8, 118.8, 114.3, 111.8, 107.8, 62.5, 55.4, 38.3, 31.6, 10.9, 7.9, 7.8. HRMS (ESI+) (*m/z*): $[M+H]^+$ calcd. for $C_{22}H_{20}NO_3S^+$, 378.1158; found, 378.1169.

Molecular Biology and Biochemistry

Site directed mutagenesis for PrfA G145S substitution and allelic replacement

10 ng of plasmid pLis35 (Sheehan et al., 1996) was amplified by PCR with a Phusion DNA polymerase (Thermo Scientific) using primers PrfA-G145S-F and PrfA-G145-R (Eiting et al., 2005) and digested overnight with 10U DpnI (Thermo Scientific) according to manufacturer's instructions. *E. coli* strain DH5α was transformed with a resulting reaction mix, transformants selected for plasmid DNA purification. Correct sequence of *prfA* gene carrying the G145S substitution was verified by sequencing the insert in the resulting plasmid pKVA609. The *prfA* G145S allele DNA fragment was excised from pKVA609 with PstI endonuclease, blunted using Mung Bean Nuclease (New England Biolabs) and ligated into SmaI site of the pMAD vector to construct the allelic replacement plasmid pKVA973. The *prfA* allele replacement with the *prfA** was performed using pKVA973 plasmid in *L. monocytogenes* strains EGDe and Δ*lmo0866* as described previously (Arnaud et al., 2004).

Protein Purification

pET28a (*prfA*)(Sheehan et al., 1996) was introduced into *E. coli* BL21(DE3) by heat-shocking. Transformants were grown in LB supplemented with kanamycin and chloramphenicol. At an OD_{600} of 0.8 the PrfA expression was induced by isopropyl β-D-1-thiogalactopyranoside (IPTG) for 3 h, and the cells were subsequently lysed by sonication. PrfA was extracted by Ni-NTA Superflow FF (Qiagen), followed by thrombin (GE-Healthcare) cleavage and dialyzed against buffer (200 mM NaCl, 10 mM Tris pH 7.5, 1 mM dithiothreitol (DTT)) at 4°C overnight. The thrombin cleaved protein had an N-terminal tail of residues GSHMASMTGGQQMGRGS followed by residues Glu7-N237. The protein was purified by MonoS 5/5 ion-exchange (GE-Healthcare). The column was eluted with a lineal gradient of 200-1000 mM NaCl, and the pure protein eluted at ~250 mM NaCl, 10 mM Tris pH 7.5, 1 mM DTT. The peak fractions of $PrfA_{WT}$ were pooled and concentrated using a Centriprep-10 centrifugal concentrator (Millipore) to a final concentration of 3.5 mg ml^{-1}.

Western blot

Overnight cultures of *L. monocytogenes* were diluted to an OD_{600} of 0.025 in BHI supplemented with DMSO, or the ring-fused 2-pyridone compounds dissolved in DMSO, resulting in a final concentration of 0.1% DMSO. The cultures were grown at 37 °C to an OD_{600} of 1.0. The supernatants and bacterial pellets were collected for protein precipitation and treated as follows:

LLO and P60 Western blotting detection: 1 ml of the culture supernatant was filtered through 0.22 µm PVDF filter (Millipore) and subjected to trichloroacetic acid (TCA) precipitation. 1/100 vol 2% sodium deoxycholate (Sigma Aldrich) was added to the samples and incubated at RT for 10 min. One fourth volume of ice cold 50% TCA was added to the samples and incubated on ice for 1 h. The samples were spun down (10 min, 18,000 rcf) and the precipitate washed in 80% ice cold acetone. The dried protein pellets were suspended in 1 × sample buffer (Laemmli, 1970) and subjected to SDS-PAGE and Western blotting using rabbit anti-LLO (#ab43018, Abcam) and HRP conjugated goat-anti rabbit secondary antibodies (#as09602, Agrisera) or anti-P60 (BioSite P6017) and HRP conjugated rabbit- anti mouse secondary antibodies (Dako P0260).

ActA, PrfA and P60 Western blotting detection: The cultures were added an equal volume of 1:1 EtOH:Ac and frozen at -20°C o/n. Subsequently the samples were centrifuged and the bacterial pellet lysed in lysisbuffer (20mM Tris pH 8.0, 50mM EDTA pH 8.0, 20% sucrose) added lysozyme and DNase. The samples were heated at 37°C for 1 hour and subjected to SDS-PAGE and Western blotting using anti-ActA and anti-PrfA R79IS4b (kindly provided by Pascale Cossart, Institute Pasteur, Paris, France) and HRP conjugated secondary antibodies (#as09602, Agrisera) or anti-p60 (BioSite P6017) and HRP conjugated rabbit- anti mouse secondary antibodies (Dako P0260).

ActA western blotting detection for intracellular bacteria: The collected bacterial pellets were resuspended in 100µl 1x sample buffer (Laemmli, 1970) and lysed by incubation at 98 °C for 20 min. 10µL of each sample was subjected to SDS-PAG and Western blotting using anti-ActA and HRP conjugated secondary antibodies (#as09602, Agrisera).

Antibody production

Production of antibodies against ActA was conducted by Agrisera. The antibodies were raised in rabbits, against a peptide mix consisting of Y21T(Kocks et al., 1993) and (NH_2-) CKDAGKWVRDKIDENPE (-$CONH_2$). The rabbits were immunized four times over a four month period, the first with FCA and the subsequent with FIA. The antisera was recovered 2 weeks after the last immunization.

Infection for determination of intracellular ActA levels

Caco-2 cells were seeded into 24 well plates (Corning #356408) and allowed to grow until confluent. Indicated bacterial strains were grown to OD_{600} = 1, added to CaCo-2 cells at a MOI of 10, centrifuged for 10 min at 800 g and allowed to infect for 30 minutes. After washing, 1ml DMEM supplemented with 100µM of **2** and 50µg/ml of gentamicin was added. For extraction of intracellular bacteria, the cells were first washed in PBS, and then lysed using PBS supplemented with 1% Triton X-100. The cell lysate was transferred to Eppendorf tubes, and centrifuged at 20238 g for 1 min. The supernatant was removed and the pellets stored at -20 °C before western blot analysis.

Hemolytic activity assay

Goat erythrocytes (Agrisera) were diluted to 10% in PBS pH 7.4. Overnight cultures of *L. monocytogenes* were diluted to an OD_{600} of 0.005 in BHI adjusted to pH 5.5 with HCl, supplemented with 0.3 M NaCl and DMSO or compounds dissolved in DMSO, resulting in a final concentration of 0.1% DMSO. The cultures were subsequently grown at 37 °C to an OD_{600} of 1.0. The supernatants were filtered through 0.22 µm PVDF filters (Millipore) and aliquoted in 96 well plates in triplicate. 10% erythrocyte solution was subsequently added to a final concentration of 5%. The samples were stored at 37 °C for 3 h, spun down and 100 µl of the supernatant measured at an optical density at 541 nm using a TECAN Infinite M200 plate reader. Samples were done with four replicates.

Northern blot

Northern blotting was performed as described previously (Tiensuu et al., 2013). In brief, 20 µg of RNA was separated on an agarose gel (1.2% agarose, 1 × HEPES buffer (20 mM HEPES, 5 mM NaAc, 1 mM EDTA, adjusted to pH 7), 7.3% formaldehyde). The gel was run in 1× HEPES buffer at 100 V for 4 h and the RNA was transferred to a Hybond–N membrane (Amersham/GE-lifesciences) by capillary transfer in 20 × SSC buffer. The membranes were cross-linked by UV-light, pre-hybridized at 60 °C in Rapid hyb buffer (Amersham/GE-lifesciences) for about 2 h and then hybridized with DNA probes at 60 °C overnight. Membranes were washed (0.5% SDS, 2 × SSC, room temperature for 15 min followed by 0.5% SDS, 0.1 × SSC 60 °C for 15 min), exposed in a phosphorimager cassette and developed using the Typhoon FLA9500 Scanner (HE Lifesciences). The probes were created by amplifying genomic *L. monocytogenes* EGDe DNA by PCR using primers BM1/BM2 for *hly*; actA-qF/actA-qR for *actA*; inlA-U/inlD for *inlA* and *inlAB*; plcA-U/plcA-D for *plcA* and *plcA-prfA* and tmRNA-U/tmRNA-D for

tmRNA. Sequences of primers are found below**.** Probes were subsequently labeled with α-32P dATP (PerkinElmer) using Megaprime DNA labelling system (Amersham/GE-lifesciences), according to the manufacturer's instructions.

Surface plasmon resonance (SPR)

SPR experiments was conducted in a Biacore X100 (Biacore/GE Healthcare), and performed mainly as described previously (Deshayes et al., 2012). In brief, biotinylated oligonucleotides (Promo40plcA-P14/Promo40plcAREV), containing the PrfA box of the *plcA* promoter, were immobilized on 1 flow-cell of a SA Chip (Biacore/GE-Healthcare). Assays were performed at 37 °C. Protein samples were diluted in HBS-EP+ (Biacore/GE-Healthcare) to a final concentration of 200 nM, and compounds at different concentrations were added, resulting in a final DMSO concentration of 0.5%. The samples were injected with a flow rate of 10 µL/min. HBS-EP+ was used as running buffer. Measurements were done with a contact time of 200 s, and a dissociation time of 120 s. This was followed by regeneration solution (0.1% SDS, 3 mM EDTA) with a contact time of 60 s. The flow-cell without any immobilized DNA was used for reference subtraction. Each run was repeated three times.

Isothermal titration calorimetry (ITC)

ITC experiments were performed using a MicroCal AutoiTC200. In a typical run, 25 automated injections of 1.65 µl with 170 s breaks in between injections were made at 25 °C with 600 rpm stirring speed on low feedback mode. The protein concentrations in the cell were varied between 25 and 100 µM while the compound concentration in the syringe was varied from 300 to 800 µM. The buffer for both protein and compound solutions was the same as in the final

step of the purification. The compounds were dissolved in DMSO, and then diluted with buffer. DMSO content in the final compound solutions did not exceed 0.5% (*v/v*). Data integration, fitting and evaluation were performed using the software Origin™ 7 with the ITC200 plugin provided by MicroCal/GE Healthcare.

Crystallography

Crystallization

Using 5' NcoI and 3' Acc651 restriction sites, the PrfA gene was cloned into a modified pET24d plasmid containing an upstream, in frame coding region for a 6-His tag and a Tobacco etch virus (TEV) protease cleavage site. The construct was transformed into the expression strain *E. coli* BL21 (DE3) pLysS (Novagen). The PrfA protein was expressed in *E. coli* as described above (Protein purification). Following cell lysis, the protein was extracted from the soluble fraction by Ni-NTA Superflow FF (Qiagen), followed by TEV protease cleavage and was dialyzed at 4°C overnight against buffer (200 mM NaCl, 20 mM NaP (NaH_2PO_4+Na_2HPO_4) buffer pH 7.1, and 1 mM dithiothreitol (DTT)). The TEV cleaved protein contains two non-native N-terminal residues GA followed by the native residues M1-N237. The protein was purified by Ni-NTA Superflow FF (Qiagen) followed by MonoS 10/10 ion-exchange (GE-Healthcare) and Superdex 75 16/60 size exclusion chromatography (GE-Healthcare). The MonoS column was eluted with a linear salt gradient between 100-1000 mM NaCl. The pure protein eluted at about 200 mM NaCl, 20 mM NaP, pH 6.5. Size exclusion chromatography was performed with the same buffer (200 mM NaCl and 20 mM NaP buffer pH 6.5). The peak fractions of $PrfA_{WT}$ were pooled and concentrated using a Centriprep-10 centrifugal concentrator (Millipore) to a final concentration of 3.5 mg ml^{-1}.

PrfA was co-crystallized with complex **1** (5 mol excess) using the hanging-drop vapor-diffusion technique at 18 °C. Crystals (0.1 × 0.4 × 0.04 mm^3) grew in 5 days after 1 μl of the protein solution (3.5 mg ml^{-1} PrfA, 200 mM NaCl, 20 mM NaP buffer, pH 6.5) was mixed with an equal volume of precipitant solution containing 20% PEG-4000, 16% isopropanol, 100 mM Na citrate pH 5.5 and allowed to equilibrate over a 1 ml solution of the precipitant in a Linbro plate (Hampton Research). Before data collection, the crystals were transferred to a cryo-protectant solution including 16 % v/v glycerol in the precipitant solution. The crystals were flash-cooled to 100 K in a cryo-stream (Oxford CryoSystems) and stored in liquid nitrogen. Diffraction data at 100 K were collected at the ESRF beamline ID29 (λ = 0.914 Å). Data collection statistics are shown in Table 2.

Phasing and refinement

Crystals of the *L. monocytogenes* PrfA-**1** complex belong to space group $P2_1$ and contain two protein chains, i.e. one biological dimer, in the asymmetric unit (dimer AB). The data set, collected from a single crystal, was processed with XDS (Kabsch, 1993) and scaled using AIMLESS from the CCP4 software suite (Bailey, 1994). The X-ray model of *L. monocytogenes* PrfA (PDB entry 2beo (Eiting et al., 2005) and X-ray intensity data from 57.0 to 2.25 Å resolution were used in molecular replacement searches with the program PHASER (Bailey, 1994; Mccoy et al., 2007) to recover the phase information. Positions of **1**, not present in the search model, were clearly visible in electron density maps and could be placed by LigandFit at site AI and BII. CC for ligand placements were 0.83 and 0.76, respectively. There is positive electron density at site BI, i.e. the symmetry-related site to AI but in monomer B, however, compound **1** could not be modelled at this site. Map inspection and manual model building was performed with COOT (Emsley and Cowtan, 2004; Emsley et al., 2010). Refinement was performed at 2.25 Å resolution with PHENIX (Adams et al., 2010; Terwilliger, 2004). Five

percent of the observed structure factors were not included in the refinement and were instead used for validation by free *R*-factor calculations. The refined structure comprises residues 2–237 of both monomers, two **1** molecules, and 22 water molecules that are well-defined in the electron density. 98% of all residues were in Ramachandran favored region, and 0% were Ramachandran outliers. Not modeled in the structure are the flexible residues 176-182 (similar to the 2beo structure). Weak electron density is also observed for one loop regions including residues 201-206 in monomer A (B-values > 200 $Å^2$). Overall the C-terminal domain is flexible with high B-values (>100 $Å^2$) for residues in the vicinity of the HTH motif (residues 155-175 and 183-214). Based on all main-chain atoms, the root-mean-square (RMS) deviations are ~0.9-1.0 Å comparing individually monomers A-B in PrfA with the reference model (PDB code 2beo, monomer A). The R_{work} and R_{free} of the final model are 20.3% and 25.4%, respectively. Refinement statistics are summarized in Table 2. Fig. 6a-c and Supplemental Figures S4 – S6 were prepared with CCP4mg (McNicholas et al., 2011). Fig. 6d-e was prepared with MOE (Chemical Computing Group Inc., 2011).

Oligonucleotides used in this work:

Oligonucleotide	Sequence
hly-U	GAAGCAAAGGATGCATCTGC
hly-D	CCATCTTTGTAACCTTTTCTTGG
actA-qF	AGCCCGGTTCCTTCGTTAAG
actA-gR	GGATTACTGGTAGGCTCGGC
inlA-U	GCAATATTAGTATTTGGCAGCG
inlA-D	CTAGATCTGTTTGTGAGACCG
plcA-U	TTTTTTACTTTCCCA

plcA-D	TGTTTTGCTCGTCTT
tmRNA-U	CGGCACTTAATATCTACGAGC
tmRNA-D	CCTCGTTATCAACGTCAAAGCC
PrfA-G145S-F	GGAAGCTTGGCTCTATTTGCTCTCAACTTTTAATCCTGACC
PrfA-G145S-D	GTCAGGATTAAAAGTTGAGAGCAAATAGAGCCAAGCTTCC

Supplemental References

Adams, P.D., Afonine, P.V., Bunkoczi, G., Chen, V.B., Davis, I.W., Echols, N., Headd, J.J., Hung, L.W., Kapral, G.J., Grosse-Kunstleve, R.W., *et al.* (2010). PHENIX: a comprehensive Python-based system for macromolecular structure solution. Acta Crystallogr D *66*, 213-221.

Arnaud, M., Chastanet, A., and Debarbouille, M. (2004). New vector for efficient allelic replacement in naturally nontransformable, low-GC-content, gram-positive bacteria. Applied and environmental microbiology *70*, 6887-6891.

Bailey, S. (1994). The Ccp4 Suite - Programs for Protein Crystallography. Acta Crystallogr D *50*, 760-763.

Chemical Computing Group Inc., S.S.W., Suite #910, Montreal, QC, Canada, H3A 2R7 (2011). Molecular Operating Environment. MOE.

Chorell, E., Pinkner, J.S., Phan, G., Edvinsson, S., Buelens, F., Remaut, H., Waksman, G., Hultgren, S.J., and Almqvist, F. (2010). Design and Synthesis of C-2 Substituted Thiazolo and Dihydrothiazolo Ring-Fused 2-Pyridones: Pilicides with Increased Antivirulence Activity. Journal of Medicinal Chemistry *53*, 5690-5695.

Deshayes, C., Bielecka, M.K., Cain, R.J., Scortti, M., de las Heras, A., Pietras, Z., Luisi, B.F., Nunez Miguel, R., and Vazquez-Boland, J.A. (2012). Allosteric mutants show that PrfA activation is dispensable for vacuole escape but required for efficient spread and Listeria survival in vivo. Mol Microbiol *85*, 461-477.

Eiting, M., Hageluken, G., Schubert, W.D., and Heinz, D.W. (2005). The mutation G145S in PrfA, a key virulence regulator of Listeria monocytogenes, increases DNA-binding affinity by stabilizing the HTH motif. Molecular microbiology *56*, 433-446.

Emsley, P., and Cowtan, K. (2004). Coot: model-building tools for molecular graphics. Acta Crystallogr D *60*, 2126-2132.

Emsley, P., Lohkamp, B., Scott, W.G., and Cowtan, K. (2010). Features and development of Coot. Acta Crystallogr D *66*, 486-501.

Kabsch, W. (1993). Automatic Processing of Rotation Diffraction Data from Crystals of Initially Unknown Symmetry and Cell Constants. J Appl Crystallogr *26*, 795-800.

Kocks, C., Hellio, R., Gounon, P., Ohayon, H., and Cossart, P. (1993). Polarized distribution of Listeria monocytogenes surface protein ActA at the site of directional actin assembly. J Cell Sci *105 (Pt 3)*, 699-710.

Laemmli, U.K. (1970). Cleavage of structural proteins during the assembly of the head of bacteriophage T4. Nature *227*, 680-685.

Mccoy, A.J., Grosse-Kunstleve, R.W., Adams, P.D., Winn, M.D., Storoni, L.C., and Read, R.J. (2007). Phaser crystallographic software. J Appl Crystallogr *40*, 658-674.

McNicholas, S., Potterton, E., Wilson, K.S., and Noble, M.E.M. (2011). Presenting your structures: the CCP4mg molecular-graphics software. Acta Crystallogr D *67*, 386-394.

Passner, J.M., Schultz, S.C., and Steitz, T.A. (2000). Modeling the cAMP-induced Allosteric Transition Using the Crystal Structure of CAP-cAMP at 2.1 Å Resolution. Journal of Molecular Biology *304*, 847-859.

Sheehan, B., Klarsfeld, A., Ebright, R., and Cossart, P. (1996). A single substitution in the putative helix-turn-helix motif of the pleiotropic activator PrfA attenuates Listeria monocytogenes virulence. Molecular microbiology *20*, 785-797.

Terwilliger, T. (2004). SOLVE and RESOLVE: automated structure solution, density modification, and model building. J Synchrotron Radiat *11*, 49-52.

Tiensuu, T., Andersson, C., Ryden, P., and Johansson, J. (2013). Cycles of light and dark co-ordinate reversible colony differentiation in *Listeria monocytogenes*. Molecular microbiology *87*, 909-924.
